# Chimera X Interface to Enhance Understanding in Biochemistry and Immunology

**DOI:** 10.1002/bmb.70025

**Published:** 2025-11-14

**Authors:** Dalpiaz Giovana, Krohn Muriel Schiling, Groehs Eduarda, Anjos André da Silva, Meireles R. Mariana

**Affiliations:** ^1^ Universidade do Vale do Rio dos Sinos (UNISINOS) São Leopoldo Rio Grande do Sul Brazil; ^2^ Universidade do Vale do Taquari (PPGCM‐Univates) Lajeado Rio Grande do Sul Brazil; ^3^ Universidade Federal do Rio Grande do Sul (PPGQ‐UFRGS) Porto Alegre Rio Grande do Sul Brazil

**Keywords:** biochemistry, bioinformatics, immunology, molecular biology, scientific education

## Abstract

Proteins are essential in biological systems, acting in transport, catalysis, and immune defense processes. However, these biomolecules' structural and functional complexity makes teaching and understanding these topics challenging. To address this difficulty, this study aimed to develop tutorials that facilitate learning and teaching about proteins based on structural analysis and molecular visualization through the interactions of the antigen–antibody complex. Thus, the ChimeraX platform was chosen as the central tool due to its intuitive interface and features that allow the manipulation and visualization of three‐dimensional molecules, such as proteins, DNA, and chemical compounds, in .pdb format. The software combines visual and analytical functions, covering from basic to advanced aspects, adapting to the user's level of knowledge. The study presented three practical tutorials: (i) presentation of the tool, (ii) focusing on immunology, and (iii) addressing aspects of biochemistry. These tutorials demonstrated how to use ChimeraX to explore the relationship between protein structure and function, highlighting topics such as molecular interactions and other relevant biochemical processes. In addition, the Tutorials 1 and 2 were validated through the application in a microbiology undergraduate class, followed by a questionnaire and CVI analysis, which confirmed their clarity, relevance, and applicability, reinforcing their role as effective resources for integrating bioinformatics into health‐related courses. Thus, the study contributes to disseminating knowledge methods and tools that enable more dynamic and accessible learning through visual and interactive approaches.

## 1 Introduction

Proteins play fundamental roles in biological systems, such as their essential roles in structural, catalytic, signaling, and regulatory function pathways. Their biological importance is evident, for example, in their participation in transporting molecules, cellular communication, and tissue repair [1]. In addition, immunoglobulin proteins stand out in the immune system, acting against pathogens through a defense system composed of antibodies and other mediators. Their metabolic and structural functions are equally important, mainly due to their catalytic activity, such as that performed by enzymes, which regulate essential metabolic reactions [2].

However, teaching and understanding the field of proteins is a significant challenge; there are different complex aspects involved in their structure determination and functionality. Proteins perform specific interactions and play diverse biological roles, requiring an interdisciplinary approach involving areas such as biochemistry, molecular biology, and immunology [3, 4]. Complex topics require teaching strategies that integrate theory and practice, facilitating an in‐depth understanding of the mechanisms that connect structural aspects to biological functions [5]. These approaches have the potential to increase both scientific knowledge and disseminate interest in these topics [6]. For these purposes, the present study presents tutorials to explore aspects of immunology and biochemistry through molecular visualization and structural analysis, reinforcing the protein roles and the structure–function relation. The tutorials base content explores interactions and bindings of an antigen–antibody complex formation.

The interaction between antigen and antibody is one of the fundamental pillars of understanding the immunological response. Antibodies are proteins responsible for identifying and recognizing foreign agents (antigens) in the body, being crucial for adaptive immunity [7]. In an infection process the lymphocyte‐B produces immunological memories, and in a second contact they could act faster and generate an immunological response by synthesizing antibodies [8]. Structurally, antibodies exhibit two antigen‐binding fragments (Fab), each one consisting of a heavy (VHH) and a light (VHL) chain variable domains (Fv) [9].

In this sense, the antigen–antibody interaction requires high affinity and specificity, which are essential to ensure the effectiveness of the immune response [10]. This efficiency results from a refined interaction influenced by several factors, including the variable regions with complementarity‐determining loops (CDRs), which contain the paratope (specific region of the antibody that recognizes the antigen), playing a determining role in binding to antigens [7]. Additionally, the antigen–antibody complex stability is maintained by several forces, such as electrostatic interactions, hydrogen bonds, hydrophobic interactions, and Van der Waals forces [10, 11].

Regardless of the complexity of molecular interactions, advancements in developing and analyzing three‐dimensional structures of antigens and antibodies were made, and have become an essential resource for understanding these factors [12]. The spatial configuration of these molecules refers to the arrangement of the atoms within these immunological complexes. These structures can be obtained through different techniques, such as X‐ray crystallography (XR) and nuclear magnetic resonance (NMR) [13]. Based on the availability of these structures in databases such as Protein Data Bank [14], it is possible to focus the studies on a deeper understanding of relevant molecules, analyzing their interactions [15].

In this context, structural bioinformatics tools are a valuable resource to highlight biomolecules' three‐dimensional structure, their atoms' distribution, and their physicochemical properties [16] Those in silico analyses are conducted using molecular modeling and simulation tools, providing insights into the dynamics and stability of the analyzed molecules [17, 18].

Among the applied resources there are the visualization platforms, which allow detailed insights of three‐dimensional structures, facilitating the analysis and revealing information that is not readily available from other sources [19, 20]. These platforms aid in uncovering hidden information about biomolecules such as protein, RNA, and DNA, playing a fundamental role in the teaching and scientific dissemination process, making complex molecular concepts more accessible and magnifying the molecules invisible to the human eye [21].

Thus, the present study aims to (1) increase the understanding of concepts related to antibody–antigen interactions from immunological, biochemical, molecular, and structural perspectives; (2) promote a bioinformatic approach that can be applied to other biological problems and/or understanding; (3) produce tutorials using the Chimera X interface to facilitate visual interpretations and guide teachers; and (4) validate the use of this type of material in education.

## 2 Presenting Chimera X Tool: A Graphical Interface for Atomic Coordinate Visualization and Analysis

Chimera X is a graphical interface designed for the visualization and analysis of atomic coordinate files, which can be obtained from databases such as the Protein Data Bank [14], or through structural bioinformatics techniques such as molecular modeling [22]. These files are typically recognized by their .pdb format, which encodes detailed information about the molecule. One of the most crucial pieces of information in these files for visualization purposes is the position of each atom in a three‐dimensional coordinate system along the *X*, *Y*, and *Z* axes.

**FIGURE 1:**
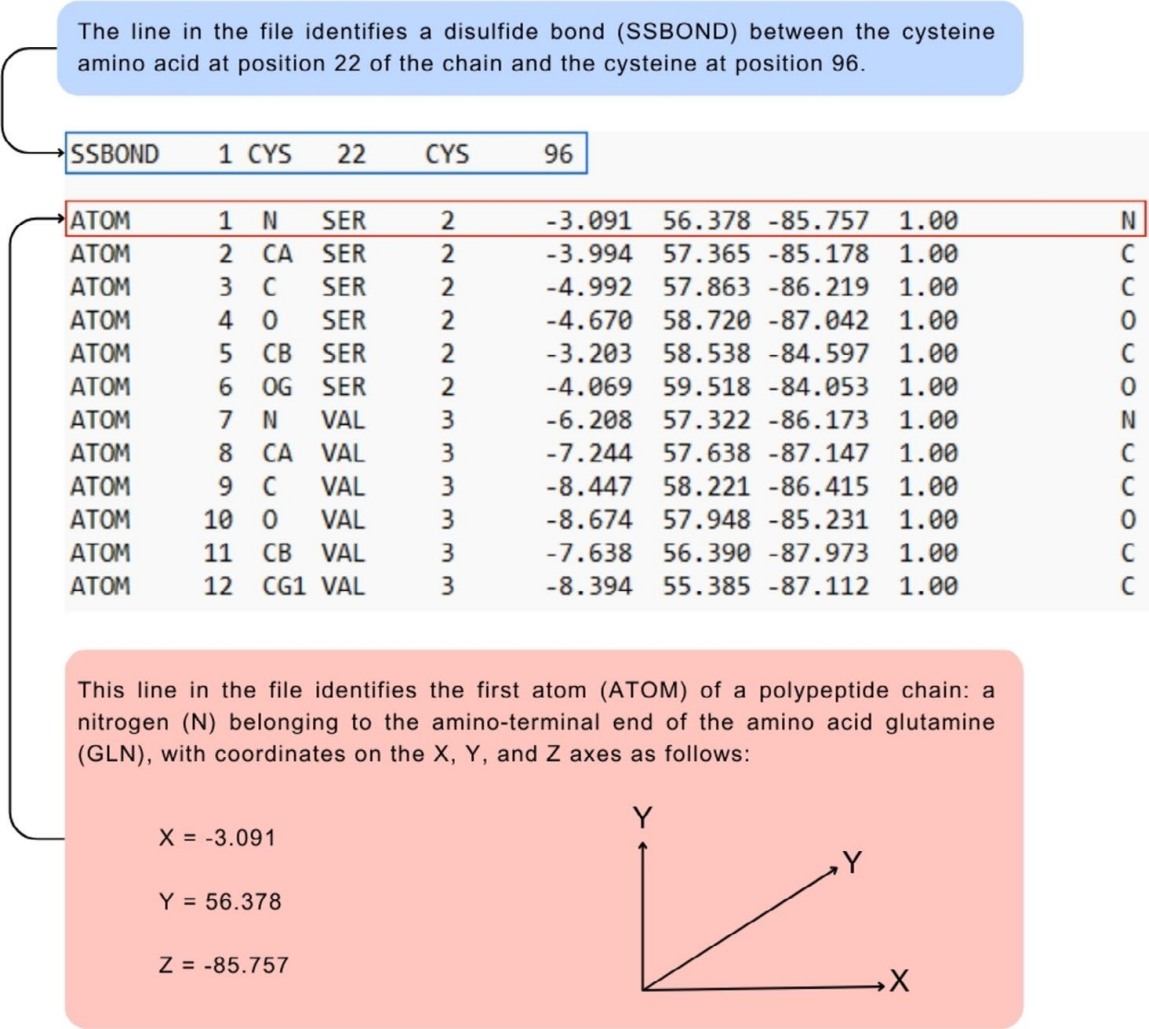
A model of an atomic coordinate file that can be implemented in the Chimera X interface for three‐dimensional representation. A partial screenshot of one file containing protein amino acids specifications presents a disulfide bond and the first 12 atoms. Each line is explained in detail to represent atoms' positions of the amino acid residues in a protein.

The visualization software processes the data from these files and spatially arranges the atoms, or even chains of atoms, into a three‐dimensional model [23]. For instance, each atom of every amino acid in the polypeptide chain is identified in a protein (consisting of a chain of amino acids extending from the N‐terminal to the C‐terminal region). The *X*, *Y*, and *Z* coordinates specified in the file describe the atoms' position [24]. Figure 1 presents a model of an atomic coordinate file with its data decoded and a schematic illustrating how atomic positions can be interpreted within three‐dimensional space by the Chimera X interface.

**FIGURE 2:**
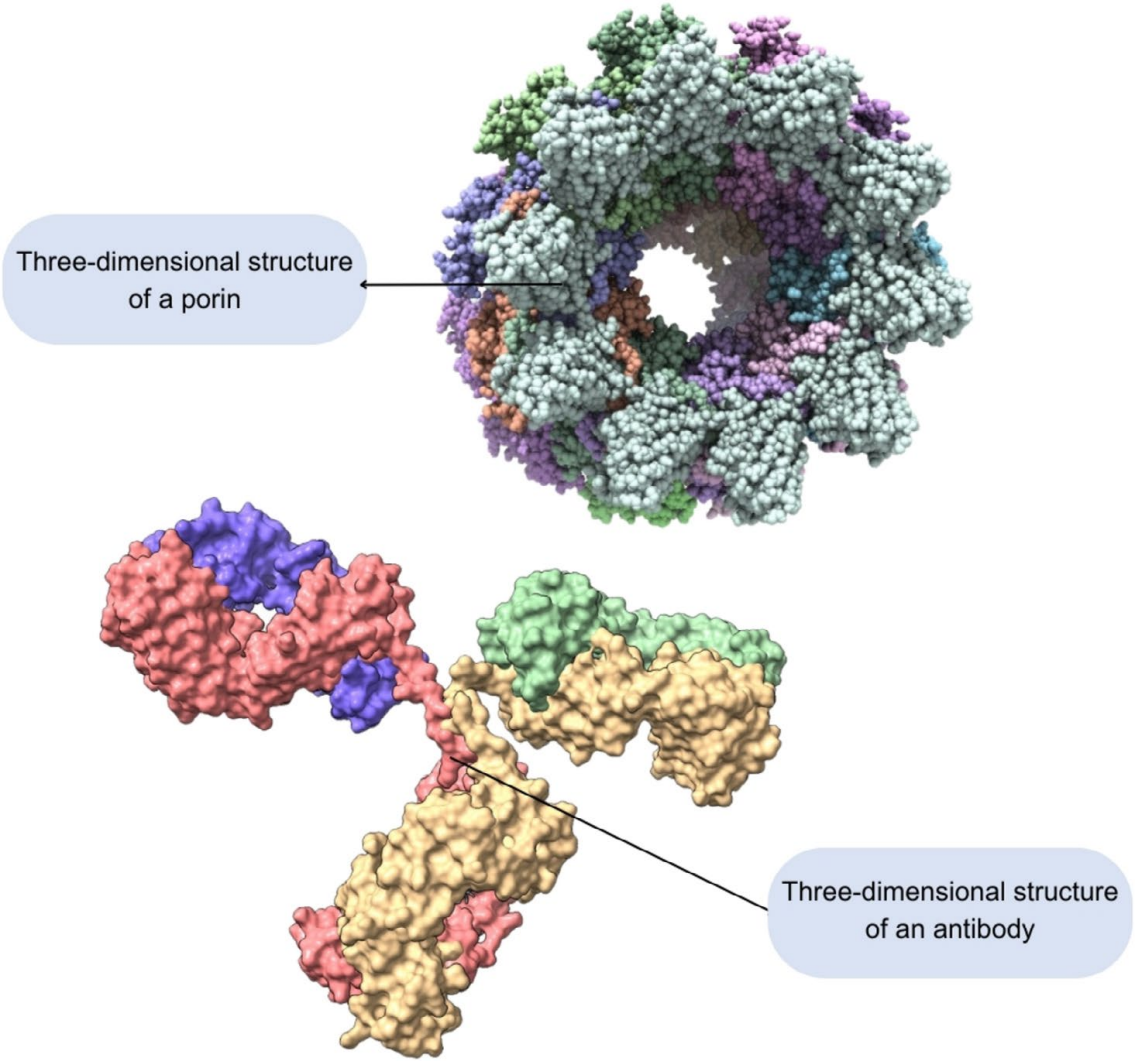
Three‐dimensional visualization of a membrane protein and an antibody using Chimera X. The figure shows the structure of these proteins, which is related to their biological functions: the passage of molecules into and out of the cell through the pore of the porin protein and the antigen‐binding fragments at the ends of the antibody.

The three‐dimensional structures of proteins provide crucial information regarding their function, cellular location, and affinity with other molecules [25]. For example Figure 2 depicts the typical structure of an antibody (*Y*‐shaped), which is related to its antigen interaction function; also a porin, one membrane protein presenting a circular conformation related to its function of selective passage of molecules into and out of the cell. The representations of both proteins were obtained through visualization using ChimeraX and their conformational structures are related to the biological functions.

Implementing coordinate files (.pdb) in Chimera X allows access to information not easily elucidated by other techniques. Among these are the spatial distribution of amino acids in the active site of a protein, as well as the formation and disruption of hydrogen bonds [26, 27]. It is important to note that Chimera X is not the only software capable of visualizing these structures; other platforms, such as PyMol and BIOVIA Discovery Studio, also offer this functionality [28]. Furthermore, Chimera X is a more intuitive version of the Chimera platform, which is available in other formats and can be downloaded from the following web address: https://www.cgl.ucsf.edu/chimerax/download.html [29].

**FIGURE 3:**
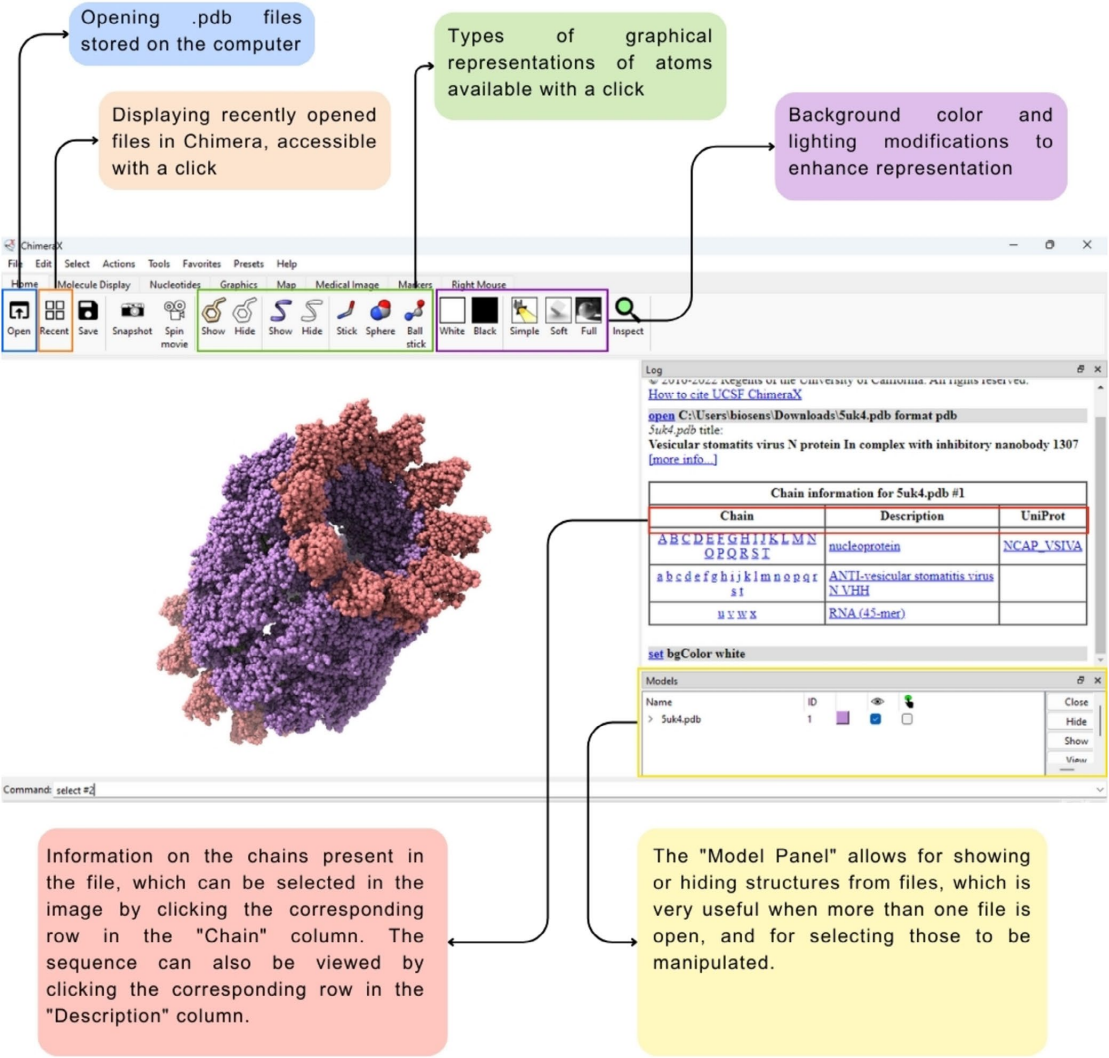
Visualization of some functionalities in Chimera X, including displaying recent structures, chain selection, sequence viewing, and model manipulation via the Model Panel [30]. *Source*: Author.

After downloading Chimera X, an icon will be generated on your desktop, providing easy access to the application's various functionalities. A distinctive feature of Chimera X is that upon opening the program, it displays the most recently used structures, which can be accessed with a simple double‐click of the mouse. When opening a structure, all its chains are listed on the right‐hand screen, allowing selection through the “Chain” column and viewing their sequences by clicking on the corresponding row in the “Description” column. From the sequence, specific amino acids can be selected using the mouse, without using the program's command line. Additionally, the “Model Panel” facilitates visualization and selection of models, also enabling movement activation (rotation and dragging of the structure) with a simple checking of the column [30]. Some of these functionalities are highlighted in Figure 3.

## 3 Methodology

Three tutorials were elaborated to increase the application of the Chimera X interface as an in silico tool in protein visualization and analysis, seeking a better understanding of the molecular aspects that consider the relation among structure and biological functions. The tutorials emphasized bullet points in immunology and biochemistry, using the interface to advance knowledge in these fields. A short explanation of each tutorial is given in the records below.

Aiming to better comprehend the different analyses that could be applied in the interface, all tutorials consider the same protein models of the Protein Data Bank, considering the biological reaction of the gp120 protein with the B12 antibody. The glycoprotein gp120 integrates the viral envelope of HIV, and the B12 antibody can neutralize its activity.

### 3.1 Tutorial 1. Introduction to Chimera X, a Tool for Visualization of the Three‐Dimensional Structure of Proteins (Supporting Information 1)

*AIMS: Promote an overview of the main commands applied in Chimera X, considering the proposed goals of the study, so that the executor can perform some important analyses using the tool*.

*Bullet points*:

- *Comprehension of the Chimera X software functions and possibilities*;
- *Software installation process*;
- *Obtention and understanding of the tridimensional structures in the Protein Data Bank*;
- *Loading and visualizing protein models in the interface*;
- *Saving and exporting results*.

### 3.2 Tutorial 2. Structural Aspects in Immunological Antigen–Antibody Interactions (Supporting Information 2)

*AIMS: Allow a visual examination of antibody domains and antigen–antibody interactions, understanding the function of the antibody chain and how both structures are organized in a three‐dimensional environment*.

*Bullet points*:

- *Comprehension of antibodies' tridimensional structures*;
- *Antigen–antibody complex interaction aspects*;
- *Intermolecular forces' role in stabilizing antigen–antibody complex*;
- *Epitope and Paratope concepts*;
- *Secondary and Tertiary conformational structures*;
- *Personalizing visualization in the Chimera X interface aiming for better recognition of structural aspects*.

### 3.3 Tutorial 3. Structural Aspects in Biochemistry (Supporting Information 3)

*AIMS: Understand the distribution of physicochemical properties by considering the tridimensional structure of proteins and infer the properties' effects in the binding process*.

*Bullet points*:

- *Amino acids' side chains determine their physicochemical properties and the effect on a protein chain*;
- *Understanding the main properties applied in silico analysis*;
- *Orientation of how to analyze the electrostatic potential and hydrophobicity property using the Chimera X interface*.

### 3.4 Validating the Application of This Type of Material Through Applying Tutorials 1 and 2 in a Classroom

Considering the elaborated tutorials and aiming to establish the advantages and usability of the produced material, Tutorials 1—“Introduction to Chimera X, a tool for visualization of the three‐dimensional structure of proteins”—and 2—“Structural aspects in immunological antigen–antibody interactions*”*—were validated through implementation in an undergraduate microbiology course. These tutorials were selected based on their alignment with the course content, which the students had been studying, in this case, virology, and the time available for the activities. Each tutorial was presented during a 90‐min class on consecutive days, with participation from 30 students enrolled in the Biomedicine, Pharmacy, and Biotechnology Engineering programs. The tutorials were translated into Portuguese to ensure students' accessibility and comprehension. The classes were conducted at a private university in the south of Brazil, with the consent and support of both the university and the course instructors. In addition, all participating students signed an informed consent form prior to their participation.

At the end of the second session, students completed an online questionnaire designed to understand both their learning outcomes and their perceptions of the tutorials. The questionnaire was structured into three blocks: (i) student profile and background information, (ii) questions aimed at evaluating learning outcomes, and (iii) Likert‐scale items focused on assessing the clarity, relevance, and applicability of the material. The complete questionnaire, with all items and response formats, is provided in Supporting Information 4. To participate in the questionnaire, students were required to read and accept an informed consent form, ensuring voluntary participation and ethical use of the collected data. This phase of the study aimed to evaluate student acceptance of the materials and determine whether the tutorials effectively supported their understanding of the virology content.

Subsequently, all other data analyses were performed using RStudio (version 4.4.2). The first analysis involved generating alluvial diagrams to visualize the flow and relationships between the students' characteristics, including their academic program, semester, and previous experience with computational tools. The ggplot2 and ggaluvial packages were employed to construct these diagrams, enabling the identification of distributions and transitions between categorical variables. This graphical approach offered an intuitive representation of the students' profile and provided contextual information to interpret subsequent results.

The evaluation of the students' responses to the tutorials was then conducted. A text mining analysis was applied to the open‐ended questions in order to capture their perceptions and learning outcomes. Preprocessing steps included conversion to lowercase, removal of punctuation, numbers, and stopwords in Portuguese, and normalization of words with similar roots. Word clouds were generated with the wordcloud and RColorBrewer packages, allowing the visualization of the most frequent terms used by the students when describing the tutorials. This representation highlighted the central concepts and the overall knowledge expressed by participants, offering qualitative evidence of comprehension and engagement.

In addition to the qualitative exploration, quantitative analyses were performed based on the percentage of correct answers in the questionnaire. Students' responses were compared to the expected correct outcomes aligned with the tutorial content, enabling an evaluation of learning effectiveness. This step provided measurable indicators of knowledge acquisition, supporting the assessment of whether the tutorials achieved their intended educational objectives.

Finally, the applicability and content validity of the tutorials were assessed using the Content Validity Index (CVI). The analysis was conducted in RStudio following methodological recommendations by Alexandre and Coluci [31] and Grant and Davis [32]. For each item evaluated, the mean and standard deviation were calculated with the dplyr package, and the CVI was derived by dividing the number of ratings above the agreement threshold by the total responses. A CVI ≥ 0.80 was considered satisfactory, reflecting adequate clarity, relevance, and representativeness of the materials.

Based on the results obtained, the necessary adjustments were first incorporated into Tutorials 1 and 2 to refine their clarity and effectiveness. Subsequently, these same adjustments were also integrated into Tutorial 3, ensuring its development already reflected the improvements observed in the earlier materials.

## 4 Results and Discussion

A specific discussion for each topic is presented throughout the tutorials, which were designed as three bioinformatics modules tailored for health‐related courses, with a particular focus on the use of ChimeraX. These tutorials not only guided students through the exploration of structural and functional aspects of biomolecules but also provided meaningful resources to support their academic development. By integrating bioinformatics into the curriculum, students were introduced to practical examples and computational tools that facilitated the understanding of complex biological concepts. Such experiences promote active learning and the development of interdisciplinary skills, while bridging theoretical knowledge with real‐world scientific practice [33].

**FIGURE 4:**
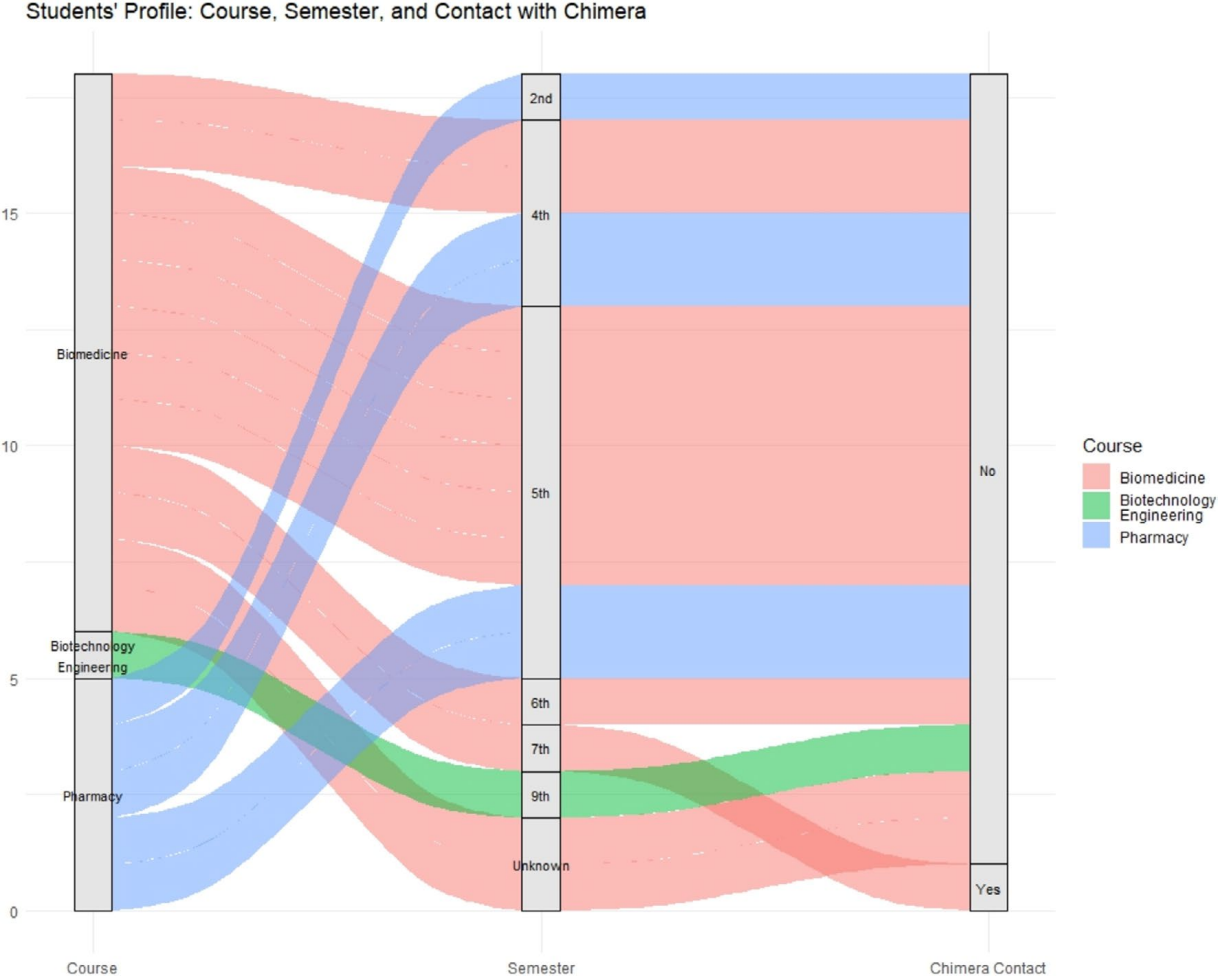
Student's profile: Course, semester, and contact with Chimera X. The alluvial diagram illustrates the distribution of participants across the courses of Biomedicine, Biotechnology, Engineering, and Pharmacy, their respective semesters, and their prior experience with the Chimera software.

The in‐class validation was conducted with a group of 30 students. At the end of the presentation of both tutorials, students were invited to complete a questionnaire voluntarily. A total of 18 students responded to the form. Among these, five were enrolled in the Pharmacy program, 12 in Biomedicine, and one in Biotechnology Engineering, the latter being an exchange student from Mexico. Based on the responses, the majority of participants were in the middle of their academic programs, with 33.33% of them enrolled in the fifth semester of an 8‐semester curriculum (Figure 4).

**FIGURE 5:**
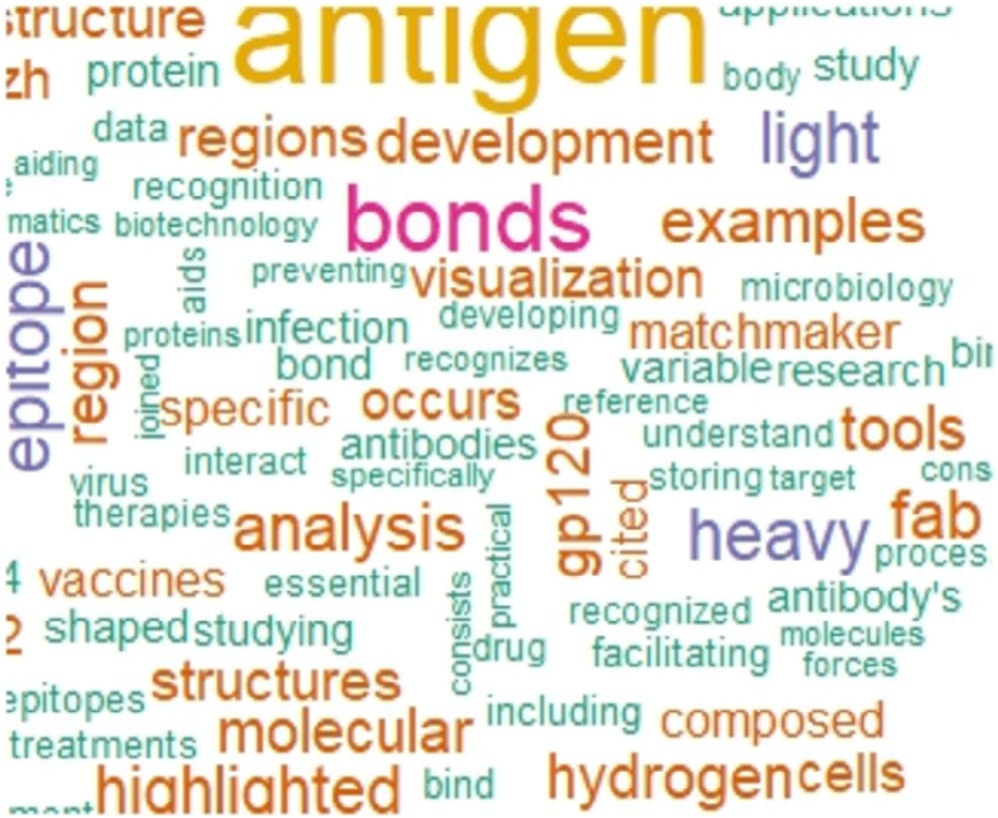
Word cloud and main topics reflecting students' understanding of the tutorial content. Word cloud generated from the analyzed textual content, highlighting the most frequent terms related to antibodies and antigens. The size of each word represents its frequency of occurrence in the dataset.

An analysis was conducted on the main terms mentioned in Part II of the questionnaire, which focused on assessing students' understanding of the tutorials. For the responses related to the tutorial, a word cloud was generated to illustrate overall knowledge on the subject, as shown in Figure 5. In addition, the key topics discussed in the responses were evaluated, with the most frequent terms including “antibody,” “antigen,” “interactions,” and “chains” across topics such as antibody and composition, structural analysis, and interactions. These terms reflect the main aspects covered during the tutorial and indicate that participants developed an understanding of both the conceptual content and the practical interactions between antibodies, antigens, and structural elements. Overall, the analysis suggests that the tutorial successfully engaged students with the core topics and facilitated comprehension of the material.

Table 1 compiles the questions asked about the tutorial, with the expected response and the percentage of correct answers for each question. It can be seen that the questionnaire included questions about the antibody and antigen used, the platform's usability, and the interaction mechanism. The results obtained for each question varied from 61.1% to 94.4%. Therefore, in all cases, at least 60% of the participants were able to answer each question correctly. It is noteworthy that the students had only two 90‐min classes to acquire and apply the knowledge from the tutorials, as this content was not previously explored in the course. Thus, the tutorials favored the acquisition of new knowledge that can already be validated through the questionnaire. The questions that generated the most incorrect answers were related to the matchmaker function and the representation of crystallographic structures. Therefore, these considerations will be used to improve the usability of the tutorial, increasing the explanation of these topics in future applications. The answers with the highest number of correct answers were related to the importance of the Protein Data Bank and the functionalities of Chimera, which is important because it shows that the students were able to understand and capture information related to the platforms covered in the tutorial, which can be applied in future studies.

**TABLE 1:** Summary of tutorial questionnaire questions and student performance.

Question	Expected answer	Percentage of right answers
What is the structure of a monoclonal antibody?	The antibody has a “Y” shaped structure and is composed of light and heavy chains, containing a Fc species‐specific region (expected to have at least one of these pieces of information)	83.3%
Explain the concept of antigen–antibody interaction, and give examples of types of binding	Antigen–antibody interaction is a specific binding between the antibody's paratope and the antigen's epitope, ensuring selective recognition. The resulting antigen–antibody complex is stabilized by interactions such as hydrogen bonds	72.2%
What is the role of glycoprotein gp120 in the HIV infection process?	gp120 is an envelope glycoprotein essential for HIV infection, as it binds to the CD4 receptor on host cells to enable viral entry. Because of this role, it is a key target for drug and vaccine development	83.3%
The structures 1HZH and 2NY7 are representations of which biomolecules in the context of the tutorial, and what are their main chains?	1HZH is a monoclonal antibody (which has light and heavy chains). 2NY7 is the gp120 protein bound to the Fab fragment of the antibody	61.1%
Explain the function of Matchmaker in Chimera X and its usefulness	The tool allows you to structurally align two crystals, using one component as a reference. It's useful for identifying similarities between different proteins	61.1%
How important is the Protein Data Bank?	The Protein Data Bank is essential because it is the main worldwide repository of crystallographic structures, freely available for use in structural bioinformatics studies	94.4%
What is the region of the antibody that interacts with the antigen called? And vice versa	In the antibody, it is called a paratope, and in the antigen, it is called an epitope	72.2%
Name a feature of the Chimera interface that enables the study of interactions	H‐bonds or Matchmaker	94.4%

The CVI analysis was performed to evaluate the clarity, relevance, and applicability of the tutorials. This step was essential to ensure that the educational material developed was not only aligned with the theoretical content but also methodologically adequate for the intended audience. The CVI is considered a robust and widely used indicator in instrument validation studies, as it quantifies the level of agreement among evaluators regarding the adequacy of each item. Values equal to or greater than 0.80 are typically interpreted as satisfactory, indicating acceptable content validity [31, 32].

**FIGURE 6:**
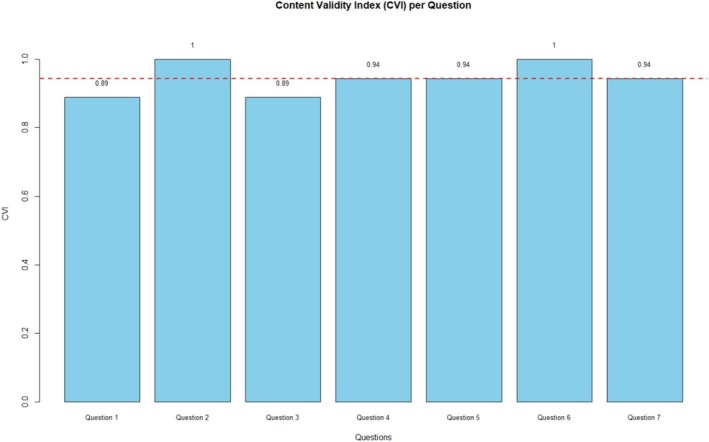
Content Validity Index (CVI) per question. The figure displays the CVI values obtained for the seven questions included in the third block of the questionnaire. The red dashed line indicates the threshold of 0.80, which is considered the minimum acceptable for satisfactory content validity. All items surpassed this cutoff, demonstrating high agreement among evaluators. The global CVI reached 0.94, confirming the strong overall validity of the educational materials.

The results obtained demonstrated high agreement among evaluators, with CVI values ranging from 0.88 to 1.00 across the seven questions analyzed. These questions were part of the third block of the questionnaire, which specifically addressed the evaluation of the tutorials' clarity and applicability, and can be observed in Table 2. Specifically, Questions 2 and 6 reached the maximum agreement score (1.00), while the lowest CVI observed was 0.88 (Questions 1 and 3), still above the threshold for satisfactory validity. The global CVI reached 0.94, confirming the strong consistency and relevance of the tutorials as a whole (Figure 6).

**TABLE 2:** Questions used to assess the clarity, relevance, and applicability of the tutorials.

Question	Evaluated aspect
Question 1	The tutorial contributed to my understanding of the concepts covered in class
Question 2	The language used in the tutorial was clear and accessible
Question 3	The content of the tutorial was relevant to the course
Question 4	The tutorial facilitated the practical application of theoretical concepts
Question 5	The tutorial facilitated the practical application of theoretical concepts
Question 6	The format of the tutorial (e.g., visual, interactive, text) was appropriate for my learning
Question 7	I would recommend the use of this type of tutorial in other classes

These results indicate that the tutorials present adequate clarity and representativeness, with only minor aspects identified for refinement. Supporting Information 5 contains the detailed raw data and the CVI calculated per question, providing transparency regarding the evaluation process and supporting the robustness of the validation procedure.

The tutorials' validation with the students proved that these materials could be effective educational resources, fostering the acquisition of new knowledge and the integration of structural bioinformatics with class content. The positive results in the questionnaire, together with the high CVI values, demonstrate that the material was clear, relevant, and applicable to the students. Although certain aspects, such as the matchmaker function, require further reinforcement, the overall responses indicate that the tutorials successfully combined theoretical and practical elements in a way that stimulated student comprehension. These findings highlight the potential of incorporating bioinformatics tutorials into undergraduate classes, contributing to the training of professionals who will better understand real‐world biological challenges.

## 5 Conclusion

Many studies explore the three‐dimensional structure of proteins to understand their interactions with drugs, the impact of alterations in the amino acid sequence on protein functionality, and changes in important properties due to mutations. Additionally, it is possible to study these structures by examining their stability and modifications over time through molecular dynamics techniques or changes in interactions with other proteins or ligands, such as drugs, through molecular docking. The knowledge linked to structural bioinformatics can be explored through various pathways and themes. This is possible using information from molecular representation interfaces combined with other robust bioinformatics tools [22, 34, 35, 36, 37].

Different challenges could emerge considering the *in silico* methods application and adhesion: the absence of both familiarity with the platforms and knowledge about what is possible to do in those interfaces is just a part of them. The presented aspects lead to sub utilizing those tools and functionalities that could enhance the advances in areas such as immunology, biochemistry, molecular biology, proteomics, and others. Also, for educators to apply these tools it is important to have access to them and highlight how to explore their subjects through those tools [38]. So, starting with an easy‐to‐use platform such as Chimera X interface for the interpretation of biological systems could encourage both educators and students to explore other bioinformatic resources [21].

Step‐by‐step tutorials allow the application of resources without fully comprehending *in silico* scripts, focusing on specific topics and applying the tools in biological contexts. Exploring real cases makes it possible for the students of biological areas to elucidate the application of bioinformatic tools, one of the reasons that the present study presented the Chimera X interface, one easy‐to‐use platform that allows a set of discoveries about the proteins and molecules' structures. From a smooth exploration of bioinformatics, it is also possible to encourage new researchers to apply those tools in their field or explore and create new tools. Even to those that do not have the intent of becoming bioinformaticians, the possibility of visualizing the molecules is interesting and insightful, contributing to different areas.

The analysis of the tutorial assessment revealed that students were able to identify and describe key structural features of proteins, such as antibodies and their interactions with antigens. The word cloud and topic analysis indicated that participants recognized important terms and concepts introduced during the tutorials, reflecting a clear understanding of both conceptual and practical aspects. These findings are consistent with the tutorial evaluation, in which the CVI indicated strong agreement among evaluators regarding the clarity and applicability of the materials. Overall, these results suggest that step‐by‐step guided exercises can effectively support learning, even for students with limited prior experience in bioinformatics.

Due to its user‐friendly interface, practical functionalities, and various forms of molecular visualization, Chimera X has great potential for use in introductory structural bioinformatics learning and more general interpretations of structures in the chemical and biological sciences. Its use by undergraduate educators can enhance students' interest in research activities and facilitate a deeper understanding of molecules and molecular interactions [39].

## Ethics Statement

Ethical review and approval were waived for this study because it involved voluntary participation of students in tutorial validation activities, posed minimal risk, and did not involve the collection of personally identifiable or sensitive data. Nevertheless, all participants provided written informed consent before completing the tutorial evaluation form.

## Conflicts of Interest

The authors declare no conflicts of interest.

## Supporting information


**Supporting Information 1.** Tutorial 1—“Introduction to Chimera X, a tool for visualization of the three‐dimensional structure of proteins.”

**Supporting Information 2.** Tutorial 2—“Chimera X: Structural aspects in immunological antigen‐antibody interactions.”

**Supporting Information 3.** Tutorial 3—“Chimera X: Structural aspects in biochemistry.”

**Supporting Information 4.** Table S1. Structure of the student questionnaire with respective blocks and items.
**Supporting Information 5**. Table S5. Raw data and Content Validity Index (CVI) for each questionnaire item.

## Data Availability

The data that supports the findings of this study are available in the Supporting Information of this article.
