# Supplementary material for: Chimera X Interface to Enhance Understanding in Biochemistry and Immunology
Source: Biochem Mol Biol Educ. 2025 Nov 14;54(1):92–102. doi: 10.1002/bmb.70025 (PMC12877970; doi:10.1002/bmb.70025)
Supplement: Supplementary file 1 — Supporting Information 1. Tutorial 1—“Introduction to Chimera X, a tool for visualization of the three‐dimensional structure of proteins.” [file BMB-54-92-s002.docx]

**TUTORIAL 1. INTRODUCTION TO CHIMERA X, A TOOL FOR VISUALIZATION OF THE THREE-DIMENSIONAL STRUCTURE OF PROTEINS**

DALPIAZ, GIOVANA.¹,

KROHN, MURIEL SCHILING.¹,

ANJOS, ANDRÉ DA SILVA.¹,

MEIRELES, MARIANA R.

¹Universidade do Vale do Rio dos Sinos

**INDEX**

[**1. INTRODUCTION TO CHIMERA X 1**](#_1d853caxytwt)

[**2. INSTALLATION OF CHIMERA X 2**](#_cfzrdrc28o1u)

[**3. SELECTION OF TARGET OF INTEREST 3**](#_lk4kiduoilbk)

[**4. OBTAINING TRIDIMENSIONAL STRUCTURES 4**](#_ptuoxplua7id)

[**5. LOADING STRUCTURES INTO CHIMERA X 6**](#_frenfbozrew7)

[**6. BASIC STRUCTURE VISUALIZATION 7**](#_k4ijo5rrr7o1)

[**7. SAVING AND EXPORTING RESULTS 11**](#_3dvs3kiwejol)

[**8. FINAL CONSIDERATIONS 12**](#_pxgpoixwzagt)

[**REFERENCES 13**](#_bo8xxhwc89hh)

## **INTRODUCTION TO CHIMERA X**

Chimera X is a software for visualizing and analyzing molecular structures developed by the Resource for Biocomputing, Visualization, and Informatics (RBVI) at the University of California, San Francisco (UCSF) (1). Its first version was released in 2002 and has garnered over 620,000 downloads. Its main features include structure analysis and sequence analysis (2), and it is possible to access introductory and training tutorials for the program on the official website (https://www.rbvi.ucsf.edu/chimerax/tutorials.html).

The tool is regarded as versatile, with application across diverse research contexts, owing to its features of being free and portable across different operating systems, graphical resources, and accessibility for users of varying experience levels. Consequently, given its significant applicability in molecule visualization, it is considered an effective and innovative tool for the scientific community (3). Despite widespread adoption within the scientific community, the interface features are still underutilized for educational purposes. However, it is believed that its application has much to contribute to the understanding and abstraction of fundamental concepts in disciplines such as biochemistry, molecular biology, immunology, chemistry, and physics.

## **INSTALLATION OF CHIMERA X**

Chimera X (version 1.7.1) is available for free download for academic, governmental, non-profit, and personal use. Installation requires following specific instructions tailored to your operating system (Windows, MacOS, Linux), as described on the tool's website (https://www.cgl.ucsf.edu/chimerax/download.html). For demonstration purposes, this tutorial will illustrate the installation and usage of a Windows system. Part 1 will demonstrate the process on the tool's website (Figure 1), while Part 2 will cover the process on your computer (Figure 2).

Figure 1 - Chimera X Installation: Part 1


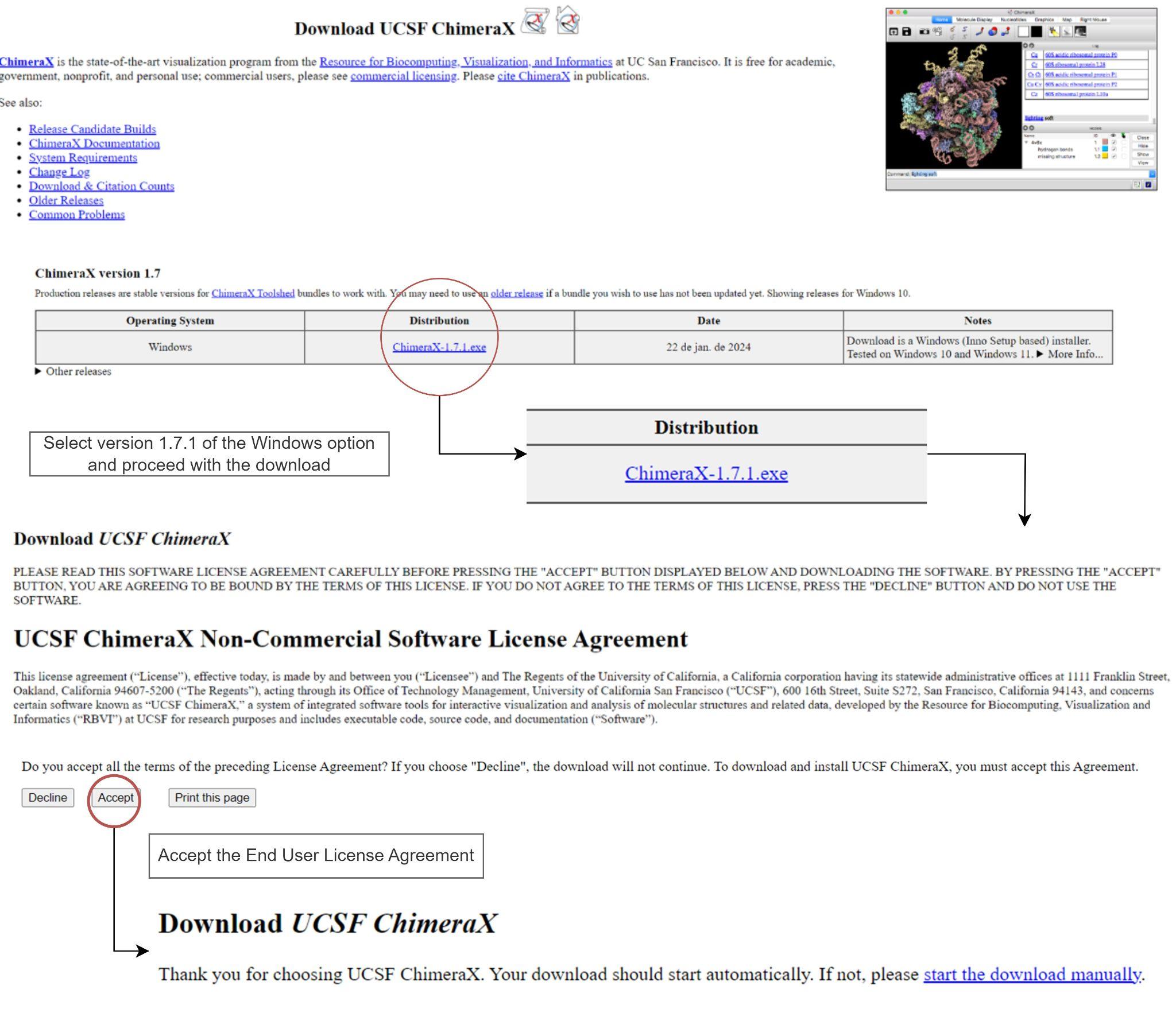


Source: Compiled by the authors

Figure 2 - Chimera X Installation: Part 2


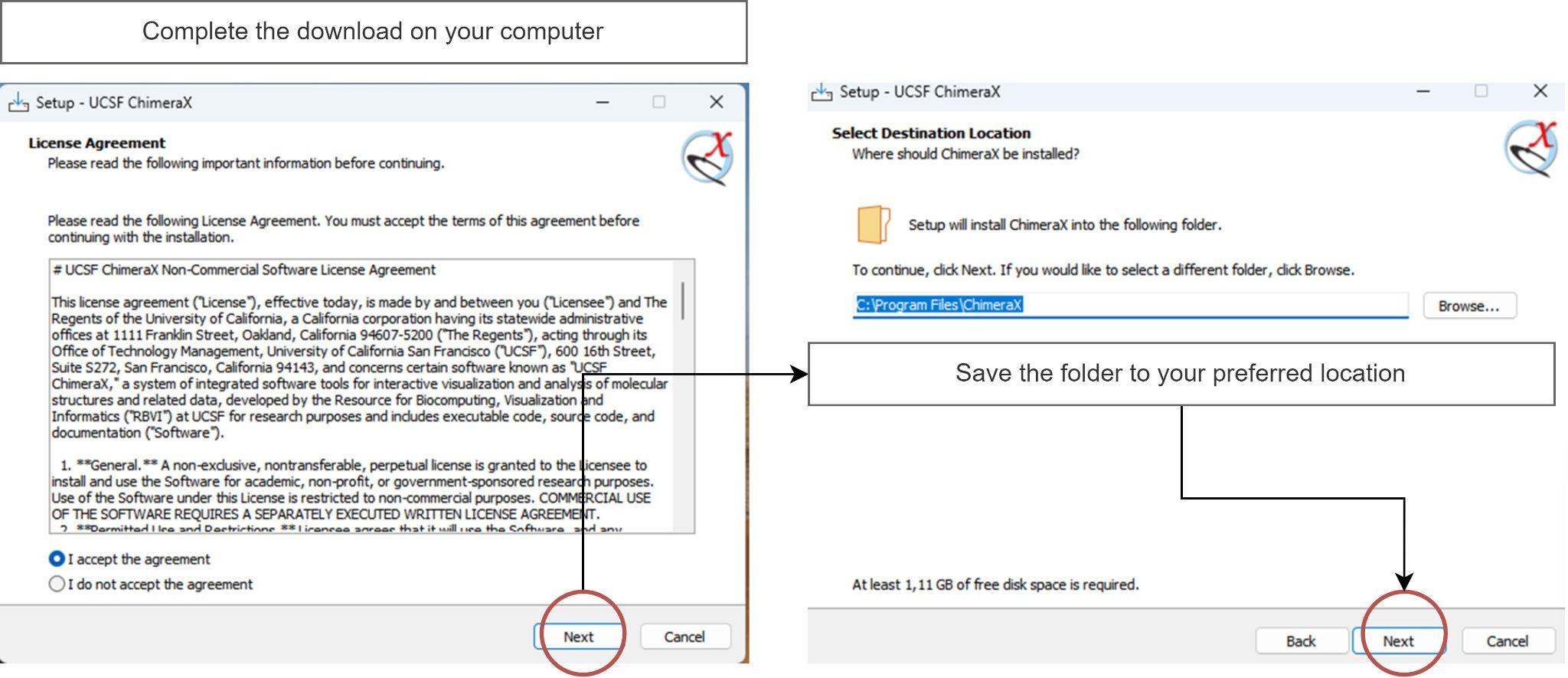


Source: Compiled by the authors

## **SELECTION OF TARGET OF INTEREST**

The first step in studying the molecule of interest is identifying the target for analysis in Chimera X. The selection process involves determining which specific parts of the structure are relevant to your analyses. The target of interest can be a protein, a protein domain, a binding site, or even a specific interaction region between a protein and a ligand (4). For exemplification purposes, this tutorial will utilize an antibody against the gp120 glycoprotein, present in the viral envelope of HIV, as the investigation target. Understanding the relationship between the antibody and the viral protein is relevant to drug development and vaccine research against HIV (5).

The monoclonal antibody B12 binds to the gp120 protein with the ability to neutralize its activity. B12 is known for blocking the CD4 binding site on the surface of gp120, thereby preventing HIV-1 entry into a target cell. Furthermore, it was one of the first neutralizing antibodies discovered, making it a research subject due to its ability to recognize different viral isolates (6). Both the structure of the B12 antibody and the structure of the gp120 protein have been elucidated through protein analysis techniques and stored in a curated database that maintains a repository of protein structures: the Protein Data Bank. The structures of the antibody and the viral protein of interest are stored under the entries 1HZH and 2NY7, respectively, and will be utilized in the proposed tutorials (7,8).

## **OBTAINING TRIDIMENSIONAL STRUCTURES**

To assess the visualization and analysis of the target in Chimera X, it is crucial to select the appropriate input data for the tool. The most commonly used file format is the .pdb, which can be directly obtained from the Protein Data Bank (PDB - https://www.rcsb.org/). PDB files contain detailed information about the atomic coordinates of biological molecules, such as proteins and nucleic acids, and are essential for structural visualization and analysis in Chimera X.

The Protein Data Bank provides relevant information for obtaining data on available crystallographic structures. To begin, use the search field where you can input the name of the target of interest or even the structure PDB code if you already know it. You can further refine the results using the provided filters on the left-hand side, such as the organism of origin (Figure 3).

Figure 3 - Obtaining .pdb Files: File Search


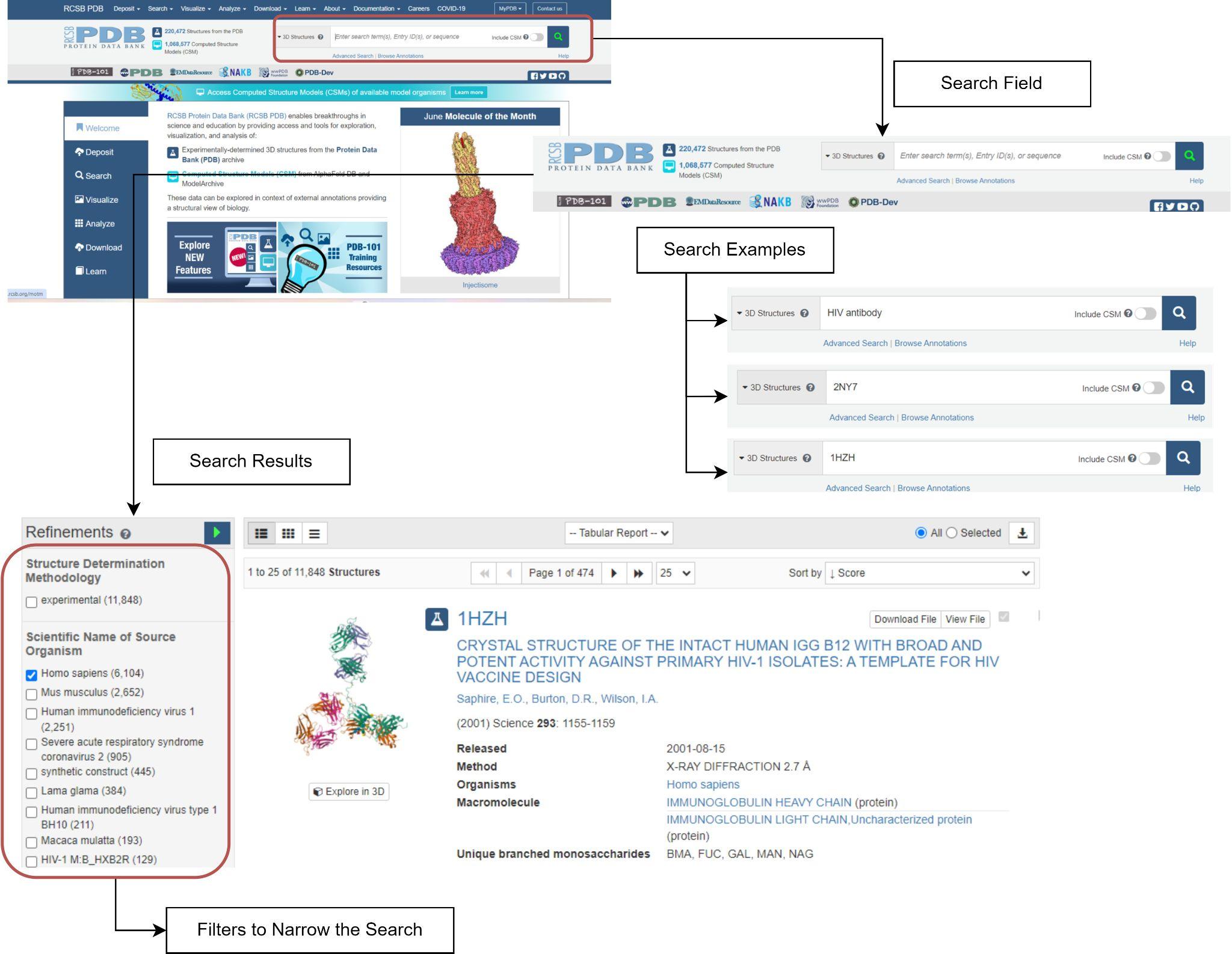


Source: Compiled by the authors

From the search result, you can select a specific structure. Clicking on it will open a page with information including molecule details, crystal acquisition method, publication date and reference, size in angstroms, and other details. If your search results in more than one PDB, choose the crystal with the smallest value in angstroms (Å), preferably opting for structures obtained through "X-RAY DIFFRACTION" method. These details are in the "Experimental Data Snapshot" section (Figure 4). Subsequently, you can download the structure in both .pdb format (atomic coordinates of the protein of interest) and FASTA format (nucleotide sequence) from the "Download Files" tab.

Figure 4 - Obtaining .pdb Files: Search Results


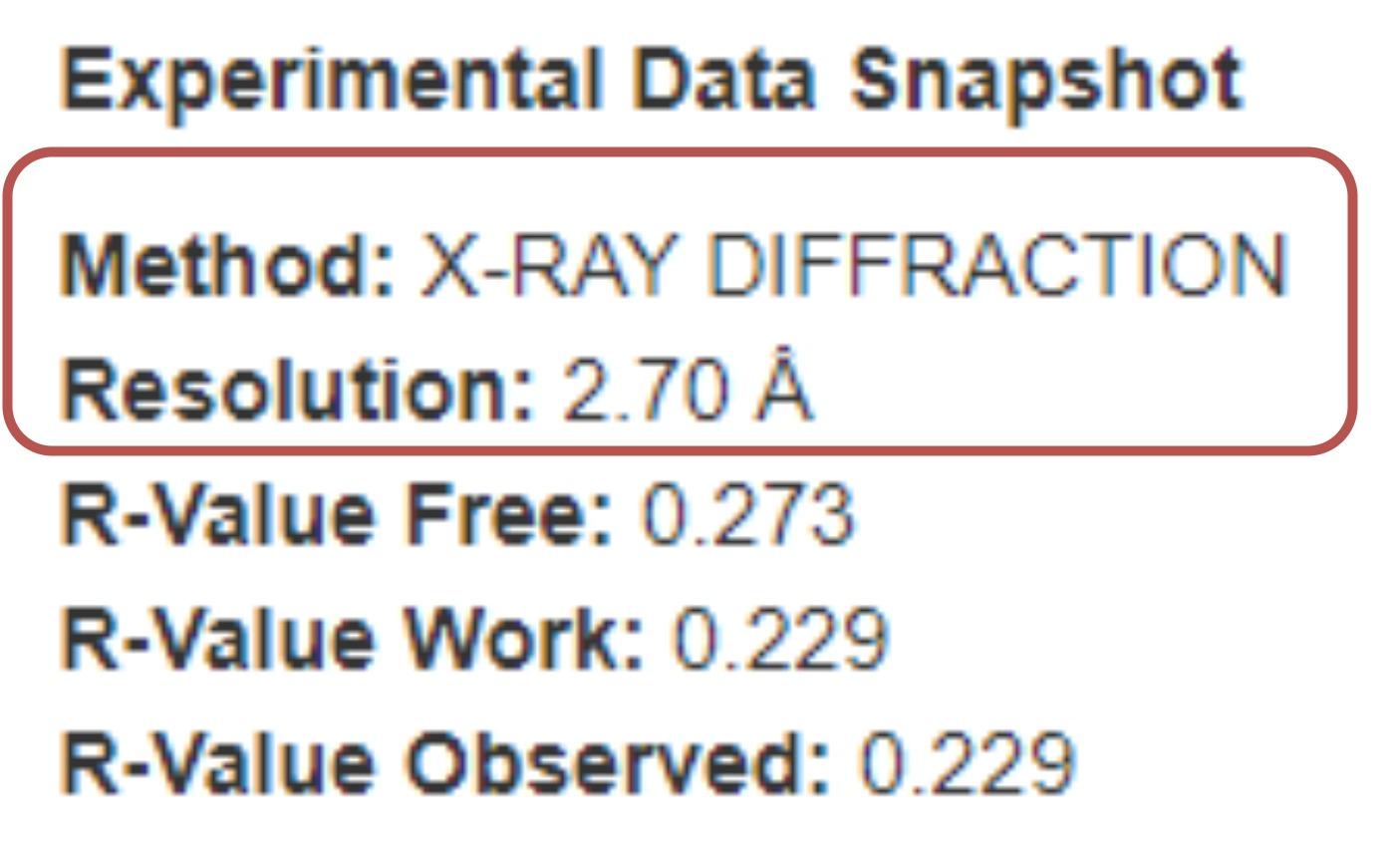


Source: Compiled by the authors

For a better understanding of how to search and utilize the Protein Data Bank, refer to Figure 5. The figure depicts a search based on the target of interest from the tutorials presented in the article, the B12 antibody, which binds to the gp120 glycoprotein of the HIV viral envelope. The figure demonstrates how to download the best .pdb file for your analysis.

Figure 5 - Obtaining .pdb Files: Practical Example


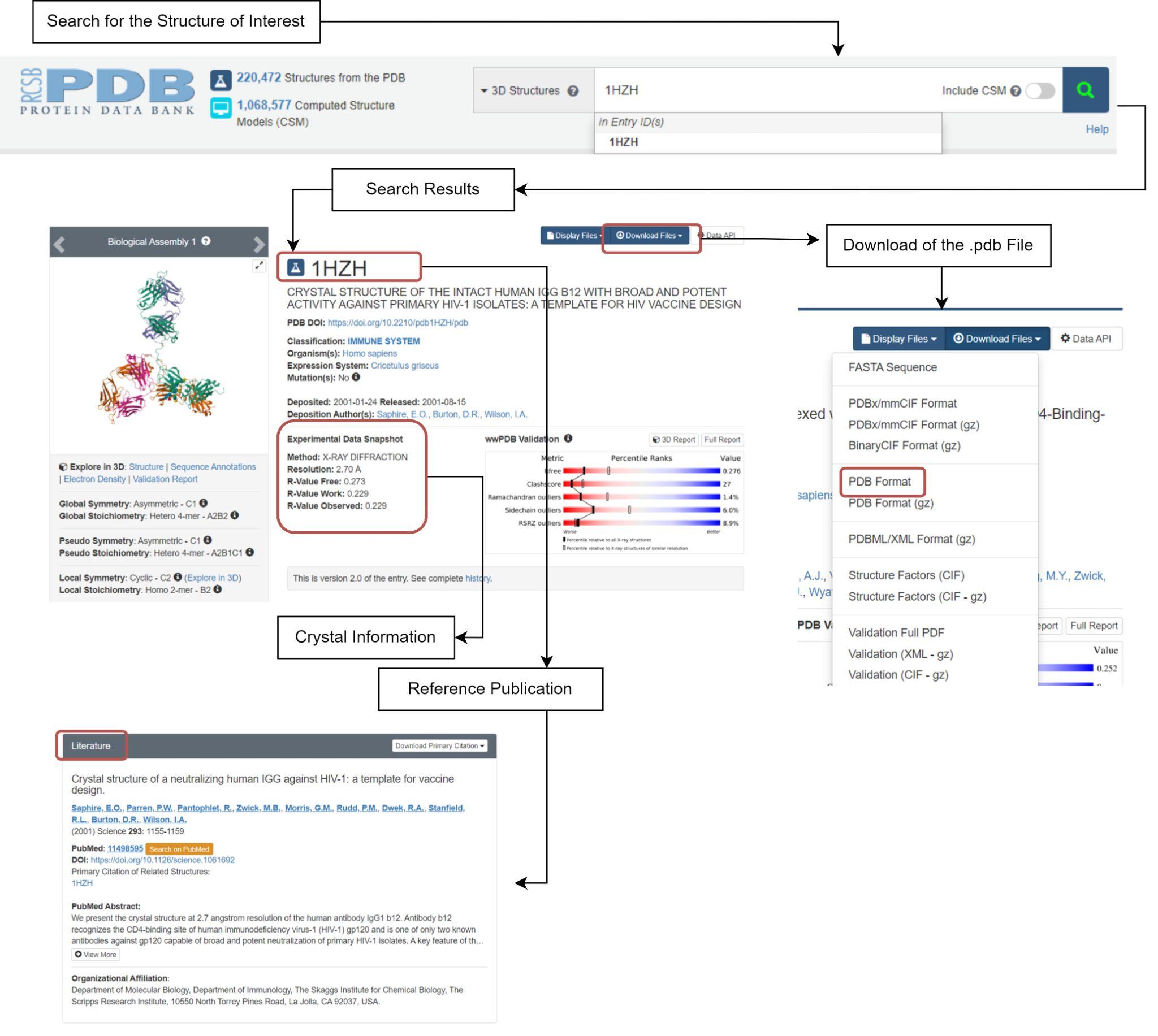


Source: Compiled by the authors

## **LOADING STRUCTURES INTO CHIMERA X**

To load PDB files into Chimera X after its installation, use the graphical interface to select them. Click on "File" in the top Menu, select the "Open" option, or click "Open" directly in the tool's header and navigate to where the PDB file is stored in your computer. Then, select the file of interest and click "Open". Once the .pdb file is loaded, you can visualize and manipulate the crystallographic structure directly within the tool. For a better understanding of this step-by-step process, refer to Figure 6.

Figure 6 - Loading Structures into Chimera X


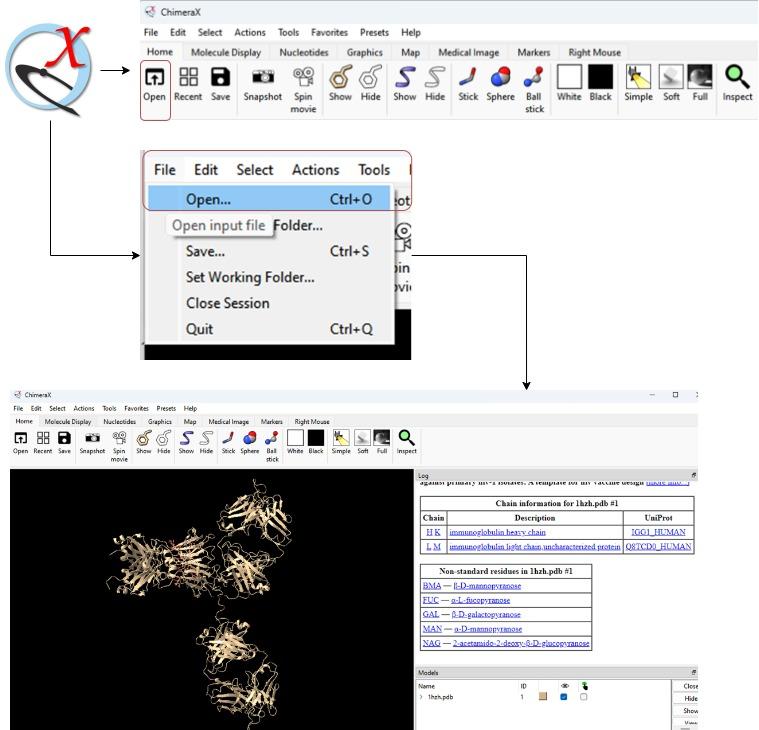


Source: Compiled by the authors

## **BASIC STRUCTURE VISUALIZATION**

Basic visualization of a structure in Chimera X is an intuitive process that allows exploring the complexities of the structures of interest. Upon loading the desired file, you can manipulate and visualize it in various ways. To view the structure from different angles, click the left mouse button and rotate it. To move the structure, use the right mouse button and drag, and to apply zoom, scroll the mouse wheel. An overview of these mouse commands is shown in Figure 7, where a schematic view of the header containing the “Right mouse” section is included. Another commonly used feature is to modify the colors of the structures, which can be done in the "Models" tab on the right side, where there is a color space called "select color" or through the Action > Colors option in the header menu (Figure 8).

Figure 7 - Structure Movement


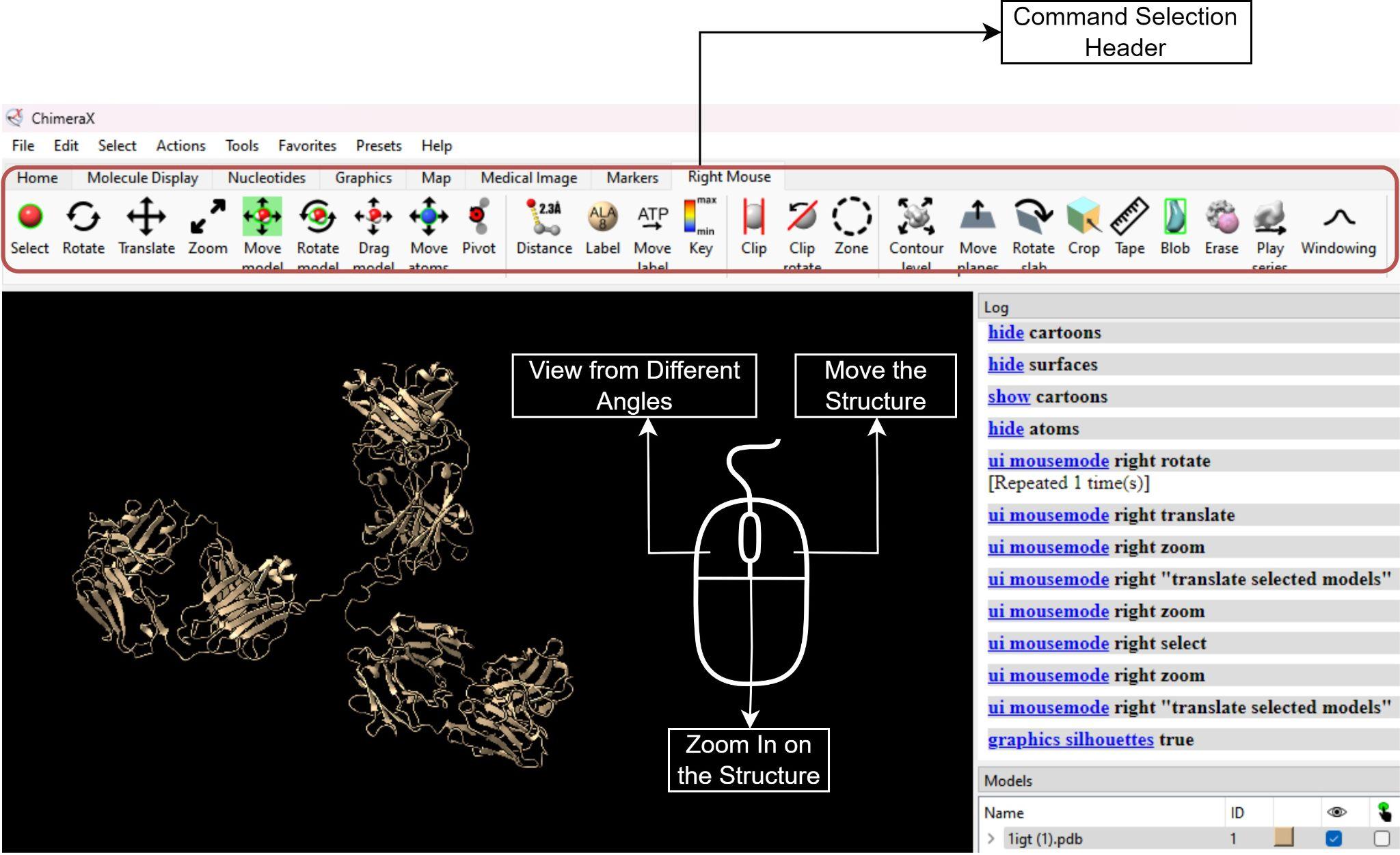


Source: Compiled by the authors

Figure 8 - Structure Color Modification


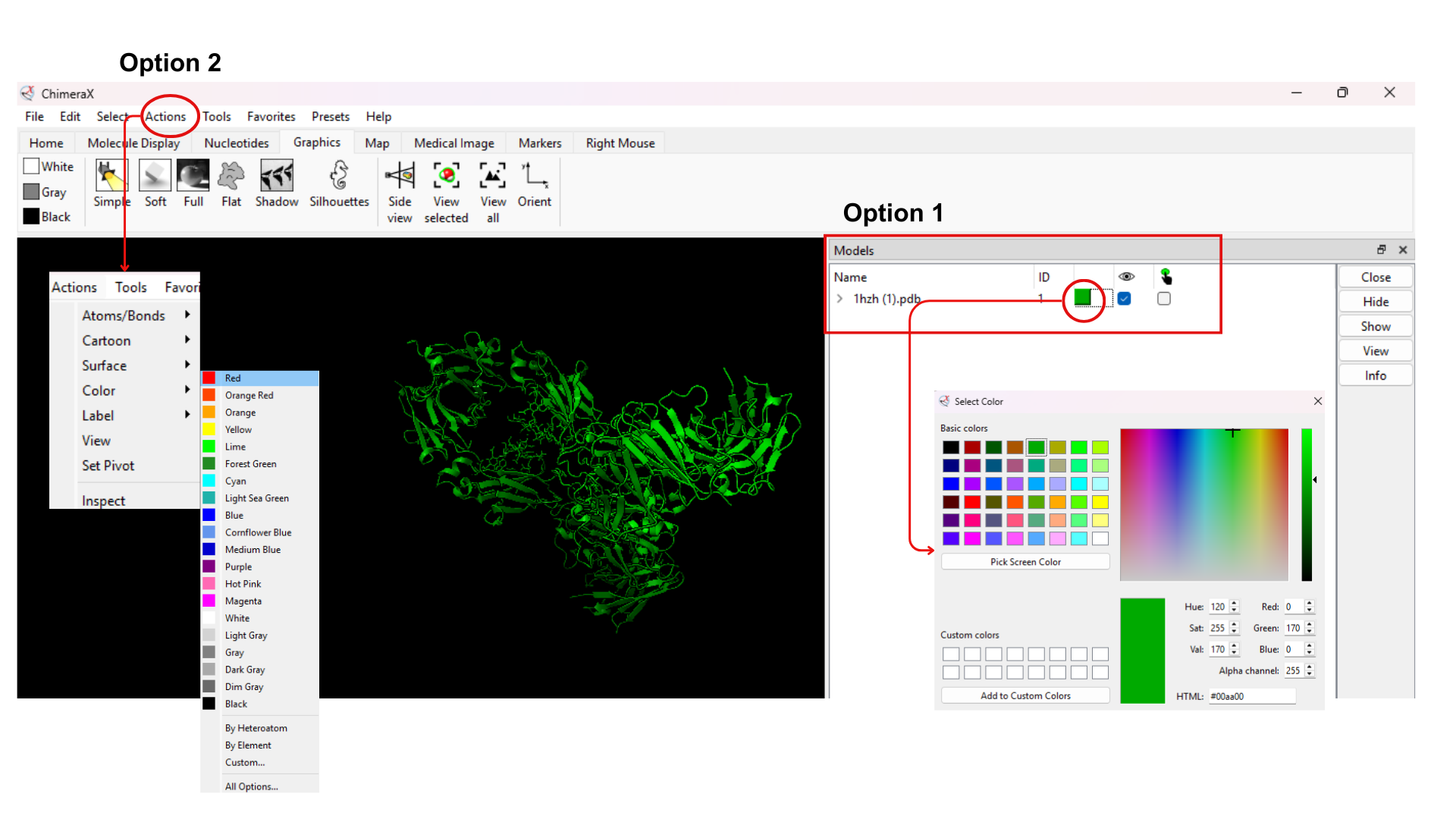


Source: Compiled by the authors

In the "Select" tab, it is possible to select atoms, residues, chains, or other parts of a structure, enabling specific manipulations and detailed analyses in a simplified manner. Figure 9 illustrates this feature and its functions.

Figure 9 - Functions of the "Select" Tab


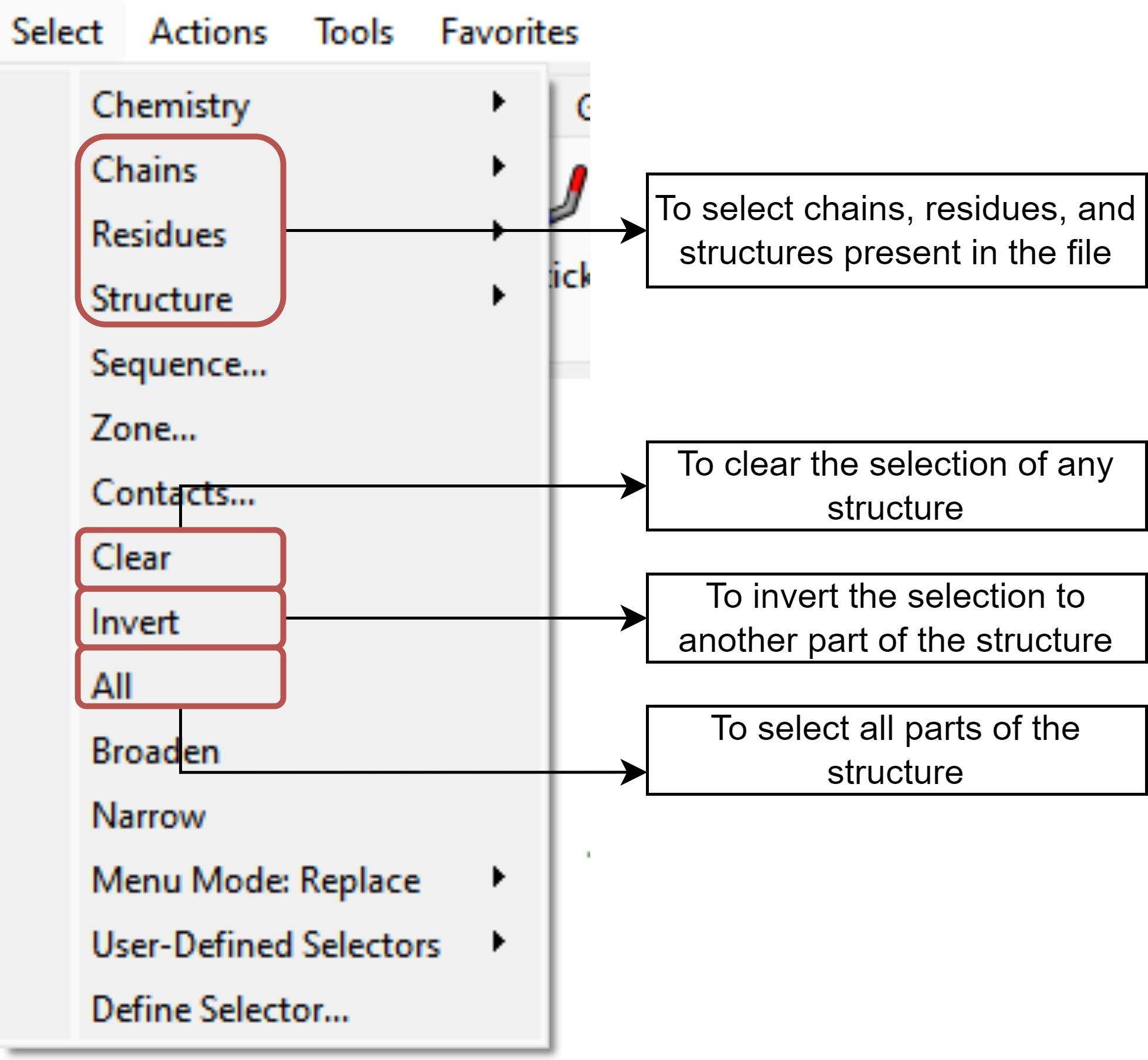


Source: Compiled by the authors

Another alternative for handling structures is through the "Model" tab, which allows selecting or hiding the structure present in ChimeraX. Additionally, users can utilize the command line to visualize and manipulate structures, enabling efficient and precise operations. The commands used are specific to each analysis and will be explained in detail when necessary in further tutorials. To use the "Models" and command line resources, it is necessary to activate them through the "Tools" tab, as shown in Figure 10.

Figure 10 - Activation of the "Models" and Command Line Functions


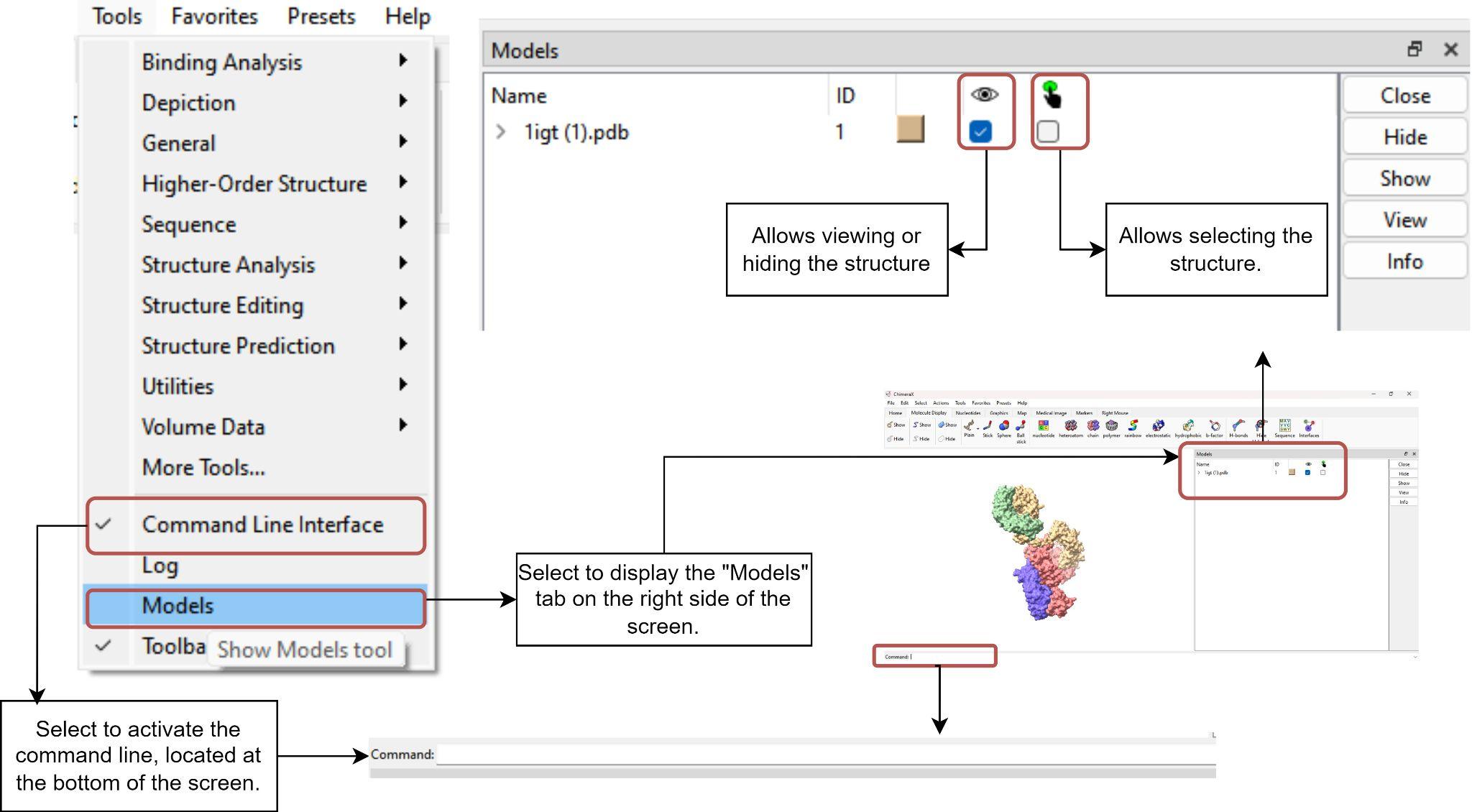
Source: Compiled by the authors

Additionally, it is possible to visualize different aspects of the structure. For example, in the "Molecule Display" tab, you can select options for surface representation of the structure, display of atoms, and representation of tertiary structure. Another important feature is located in the "Graphics" tab, where you can select the image's background in white, black, and gray colors, and you can also add a silhouette, which enhances visualization (Figure 11). Further analysis possibilities and resources will be covered in the upcoming tutorials.

Figure 11 - Structure Surface Modification and Graphic Resources


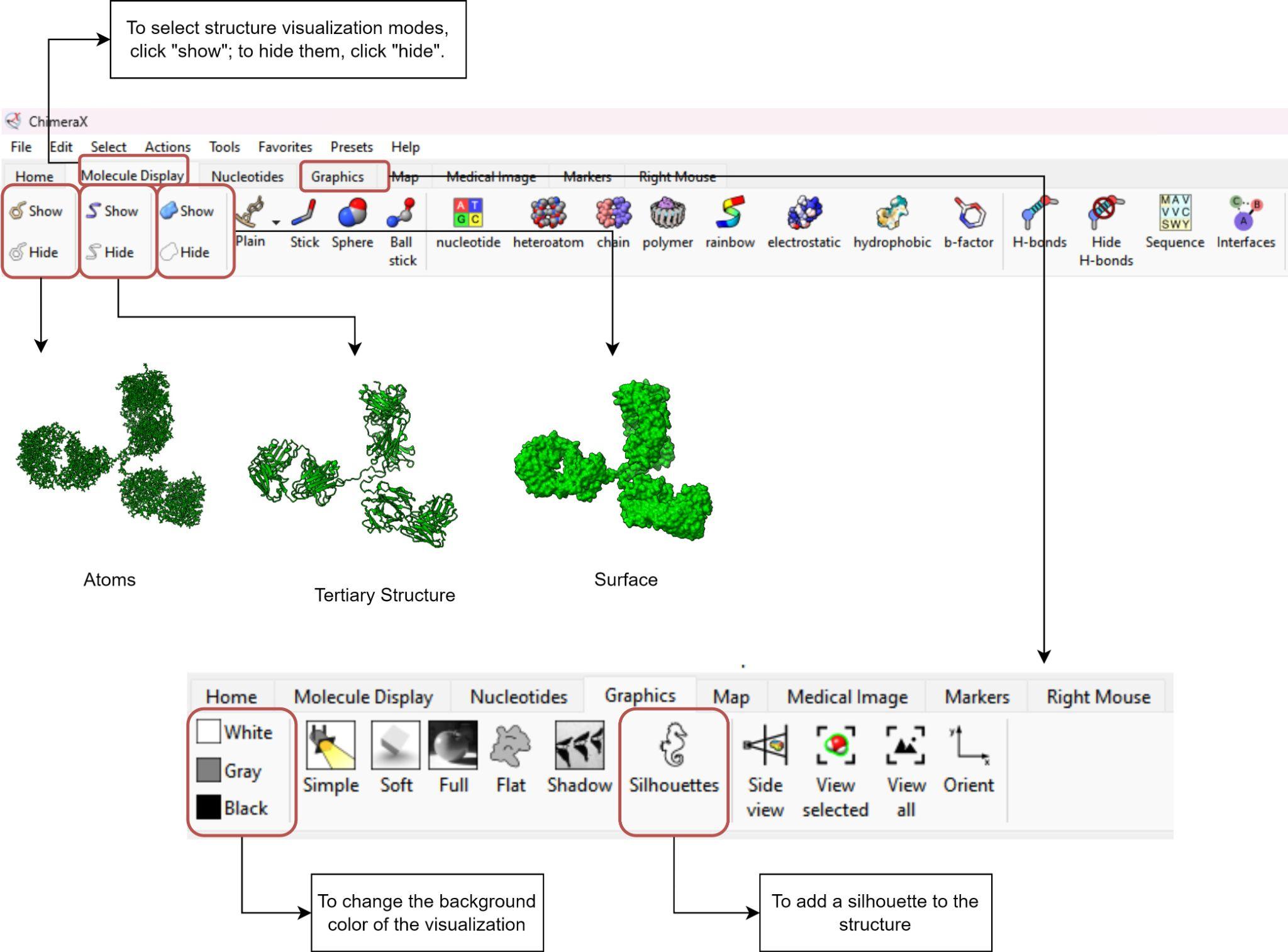


Source: Compiled by the authors

## **SAVING AND EXPORTING RESULTS**

To save analyses performed in Chimera X, it is possible to export images, videos, modified structures, and analysis data. To save images, click on "File" in the top menu and select "Save Image," then choose the desired format, such as PNG or JPEG, and click "Save." You can also save in .pdb format, which is important for analyses where structural modifications have occurred, allowing you to save all changes made during the analysis. To do this, click on "File" and select the "PDB" option, then choose the best location and name for the modified file (Figure 12). These features allow you to utilize the results of the tools directly in your research.

Figure 12 - Saving and Exporting Results


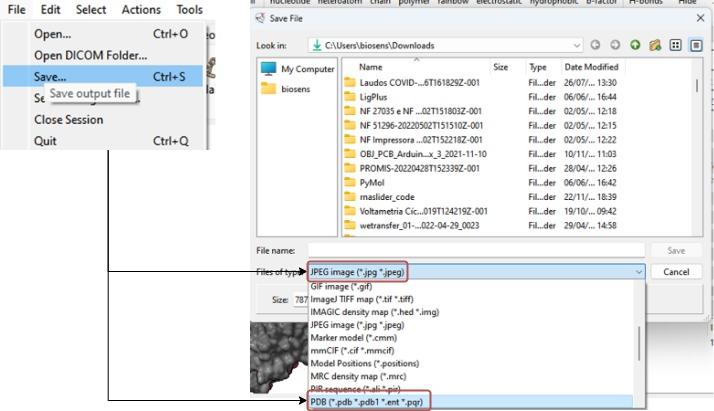


Source: Compiled by the authors

## **FINAL CONSIDERATIONS**

Based on the content covered in this initial tutorial, you can now access the Chimera X tool and utilize some of its available features. Subsequent tutorials will explore specific aspects more deeply, making it essential to complete this first tutorial as it serves as the foundation for the others.

According to the specific study theme, the presented tutorials are defined as:

- T2 - Tutorial 2: Structural aspects in immunological Antigen-antibody reaction (Supplemental Material 2)
- Tutorial 3 Chimera X: Structural aspects in biochemistry (Supplemental Material 3)

## **REFERENCES**

1. Goddard TD, Huang CC, Meng EC, Pettersen EF, Couch GS, Morris JH, et al. UCSF ChimeraX: Meeting modern challenges in visualization and analysis. Protein Sci [Internet]. 2018;27(1):14–25. Available from: http://dx.doi.org/10.1002/pro.3235

2. Pettersen EF, Goddard TD, Huang CC, Meng EC, Couch GS, Croll TI, et al. UCSF ChimeraX: Structure visualization for researchers, educators, and developers. Protein Sci [Internet]. 2021 [cited 2024 Jun 4];30(1):70–82. Available from: http://dx.doi.org/10.1002/pro.3943

3. Pettersen EF, Goddard TD, Huang CC, Couch GS, Greenblatt DM, Meng EC, et al. UCSF Chimera—A visualization system for exploratory research and analysis. J Comput Chem [Internet]. 2004;25(13):1605–12. Available from: http://dx.doi.org/10.1002/jcc.20084

4. Zhao J, Cao Y, Zhang L. Exploring the computational methods for protein-ligand binding site prediction. Comput Struct Biotechnol J [Internet]. 2020 [cited 2024 Jun 4];18:417–26. Available from: http://dx.doi.org/10.1016/j.csbj.2020.02.008

5. Cohen YZ, Dolin R. Novel HIV vaccine strategies: overview and perspective. Ther Adv Vaccines [Internet]. 2013 [cited 2024 Jun 5];1(3):99–112. Available from: http://dx.doi.org/10.1177/2051013613494535

6. Li X, Grant OC, Ito K, Wallace A, Wang S, Zhao P, et al. Structural analysis of the glycosylated intact HIV-1 gp120–b12 antibody complex using hydroxyl radical protein footprinting. Biochemistry [Internet]. 2017;56(7):957–70. Available from: http://dx.doi.org/10.1021/acs.biochem.6b00888

7. Saphire EO, Burton DR, Wilson IA. Crystal structure of the intact human igg b12 with broad and potent activity against primary HIV-1 isolates: A template for HIV vaccine design [Internet]. Worldwide Protein Data Bank; 2001. Available from: http://dx.doi.org/10.2210/pdb1hzh/pdb

8. Zhou T, Xu L, Dey B, Hessell AJ, Van Ryk D, Xiang SH, et al. HIV-1 gp120 Envelope Glycoprotein Complexed with the Broadly Neutralizing CD4-Binding-Site Antibody b12 [Internet]. Worldwide Protein Data Bank; 2007. Available from: http://dx.doi.org/10.2210/pdb2ny7/pdb
