# Supplementary material for: Chimera X Interface to Enhance Understanding in Biochemistry and Immunology
Source: Biochem Mol Biol Educ. 2025 Nov 14;54(1):92–102. doi: 10.1002/bmb.70025 (PMC12877970; doi:10.1002/bmb.70025)
Supplement: Supplementary file 2 — Supporting Information 2. Tutorial 2—“Chimera X: Structural aspects in immunological antigen‐antibody interactions.” [file BMB-54-92-s003.docx]

**TUTORIAL 2 CHIMERA X: STRUCTURAL ASPECTS IN IMMUNOLOGICAL ANTIGEN-ANTIBODY INTERACTIONS**

DALPIAZ, GIOVANA.¹,

KROHN, MURIEL SCHILING.¹,

ANJOS, ANDRÉ DA SILVA.¹,

MEIRELES, MARIANA R.

¹Universidade do Vale do Rio dos Sinos

**INDEX**

[**1. INTRODUCTION 1**](#_bf12ugixbud4)

[**2. OBJECTIVES 1**](#_1d853caxytwt)

[**3. THEORETICAL BASIS 2**](#_cfzrdrc28o1u)

[**4. METHODOLOGICAL PROCEDURES 2**](#_lk4kiduoilbk)

[**4.1. Concept 1: Visualization of antibody and antigen chains 2**](#_dc59owiuvevr)

[**4.2. Concept 2: Interaction between the antigen-antibody complex 8**](#_oilui2xxtqeh)

[**4.3. Concept 3: Antigen-antibody binding analyses 10**](#_5swo5ua3v01e)

[**5. QUESTIONNAIRE 14**](#_xvx26vi0hyk5)

[**6. CONCLUSION 14**](#_jmkpbkgc1dbw)

[**REFERENCES 15**](#_bo8xxhwc89hh)

## **INTRODUCTION**

This tutorial will demonstrate approaches to structural bioinformatics techniques in Chimera X (version 1.7.1), relating them to immunology concepts. For this purpose, a case of a monoclonal antibody B12 against HIV and the glycoprotein gp120 antigen will be applied. For a better understanding of the procedures, the related concepts will be addressed before each step of the process. However, later, as a theoretical basis, the role of gp120 and the antibody in the immune response to infection caused by HIV will be elucidated. Thus, by executing all the steps and analyzing the results provided in the tutorial, a broader understanding of the immunological theoretical concepts is expected.

## **OBJECTIVES**

This structural bioinformatics tutorial, focusing on immunology using Chimera X, aims to:

1. Provide an understanding of the main concepts related to immunology, especially the antigen-antibody complex.
2. Guide students in opening structures in Chimera X and customizing the views, including assigning colors and configuring the background and contours.
3. Instruct structural alignment analyses and identification of the interaction regions of the antigen-antibody complex.
4. Demonstrate and identify the intermolecular forces that stabilize the antigen and antibody interaction.

## **THEORETICAL BASIS**

The HIV immune response is complex and involves several steps throughout the infection, including interacting viral proteins with cellular receptors. Upon contact with the virus, host cell proteins are incorporated into the lipid layer of the viral envelope, which contains an anchored glycoprotein (gp120). Thus, an interaction occurs between HIV and target cells through some receptors and gp120 (1).

The defense role of neutralizing antibodies is highlighted in the immune response to viral infections, such as HIV. Their action includes binding to specific epitopes on the virus's surface, often on the gp120 glycoproteins, causing a blockage of interactions and preventing the infection of new cells (2). In this sense, a detailed understanding of these interactions and developing effective neutralizing antibodies are essential for preventing and treating HIV infection.

## **METHODOLOGICAL PROCEDURES**

The following topic will cover three different concepts, which will be used to develop procedures for performing analyses provided by Chimera X and their relationship with Immunology. It is also important not to translate the pages so that the names and steps of the procedures are not changed.

### **4.1. Concept 1: Visualization of antibody and antigen chains**

Antibodies, or immunoglobulins (Ig), are glycoproteins produced by the immune system in response to the presence of antigens. Structurally, they are composed of a quaternary structure consisting of four interconnected polypeptide chains: two heavy chains (H) and two light chains (L), linked by disulfide bridges (covalent bond between two sulfur atoms) in the shape of a Y (3).

Antibodies possess a variable region (Fab) composed of the antigen-binding domains of the heavy and light chains, which is specific for a particular antigen. On the opposite side, it contains the constant region (Fc), which determines the class and biological function of the antibody. In this sense, the connection between the Fab and Fc regions is essential for the structure and function of antibodies (4).

Antigens do not have a specific chain like antibodies, but they are fundamental in triggering an adaptive immune response. The structure of antigens can vary depending on the type of molecule, such as proteins, polysaccharides, lipids, and other substances recognized by the immune system as foreign. It is worth noting that the antigen-binding region in antibodies is specific to form the antigen-antibody complex (5).

Figure 1 shows a graphical summary of the concepts explained, which are important to execute the tutorial procedures. In this first part, steps 1 and 2 will be performed, allowing the visualization, handling, and alignment of the three-dimensional structures. All approaches presented in this tutorial will apply the previously defined HIV target: the monoclonal antibody B12 and the glycoprotein gp120 and their .pdb files 1HZH and 2NY7, respectively.


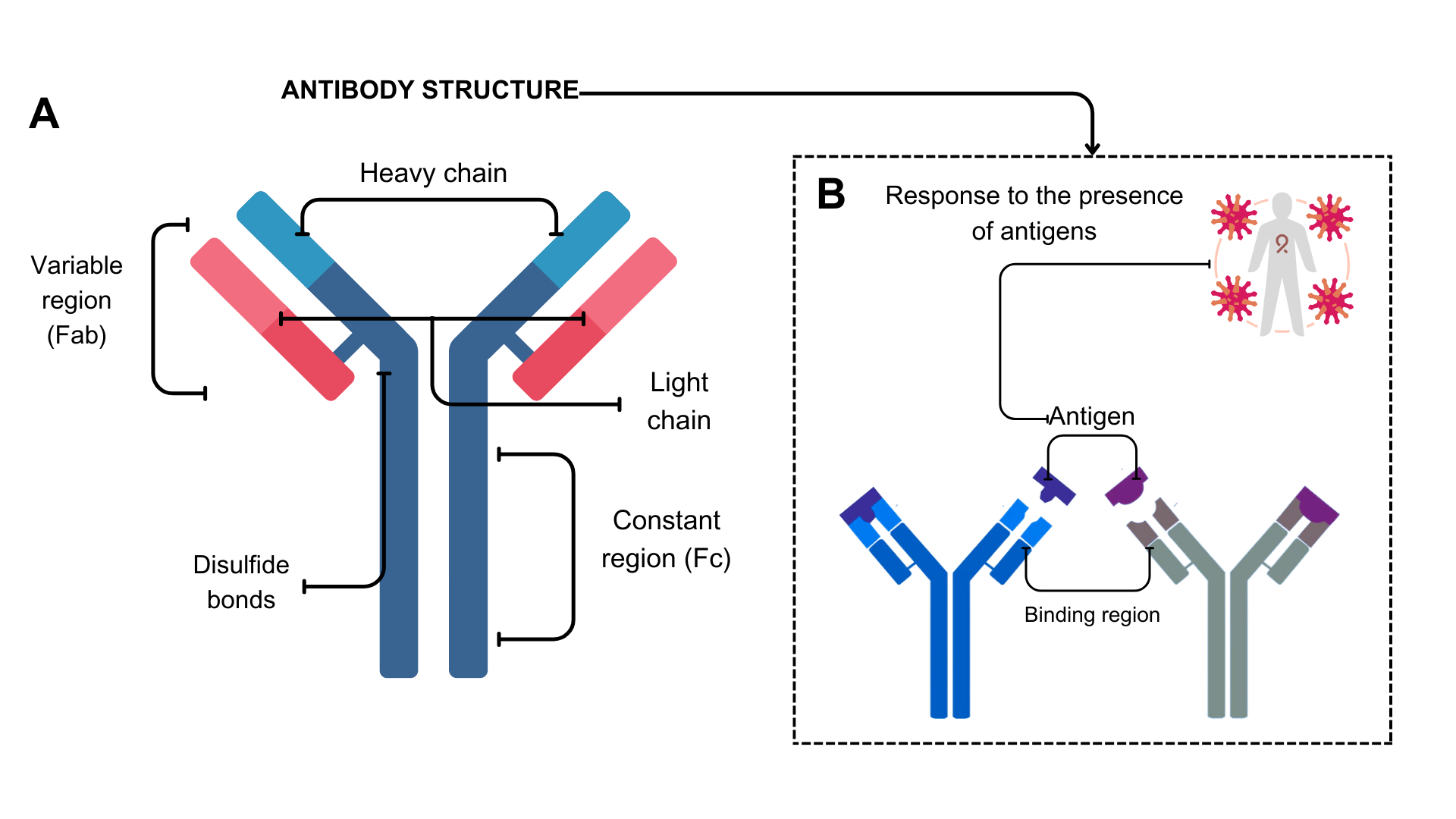


Figure 1. In representation A, the antibody structure comprises two heavy chains (blue) and two light chains (pink) joined by a disulfide bond. In addition, the variable (Fab) and constant (Fc) regions are shown, which are responsible, respectively, for direct and specific binding to the antigen and determination of the biological function of the antibody. In Figure B, the response to the presence of antigen is represented, with antibodies having binding sites specific to the target antigen. Source: Adapted from Janeway et al., 2001 (3).

Source: Compiled by the authors

- Step 1:

| 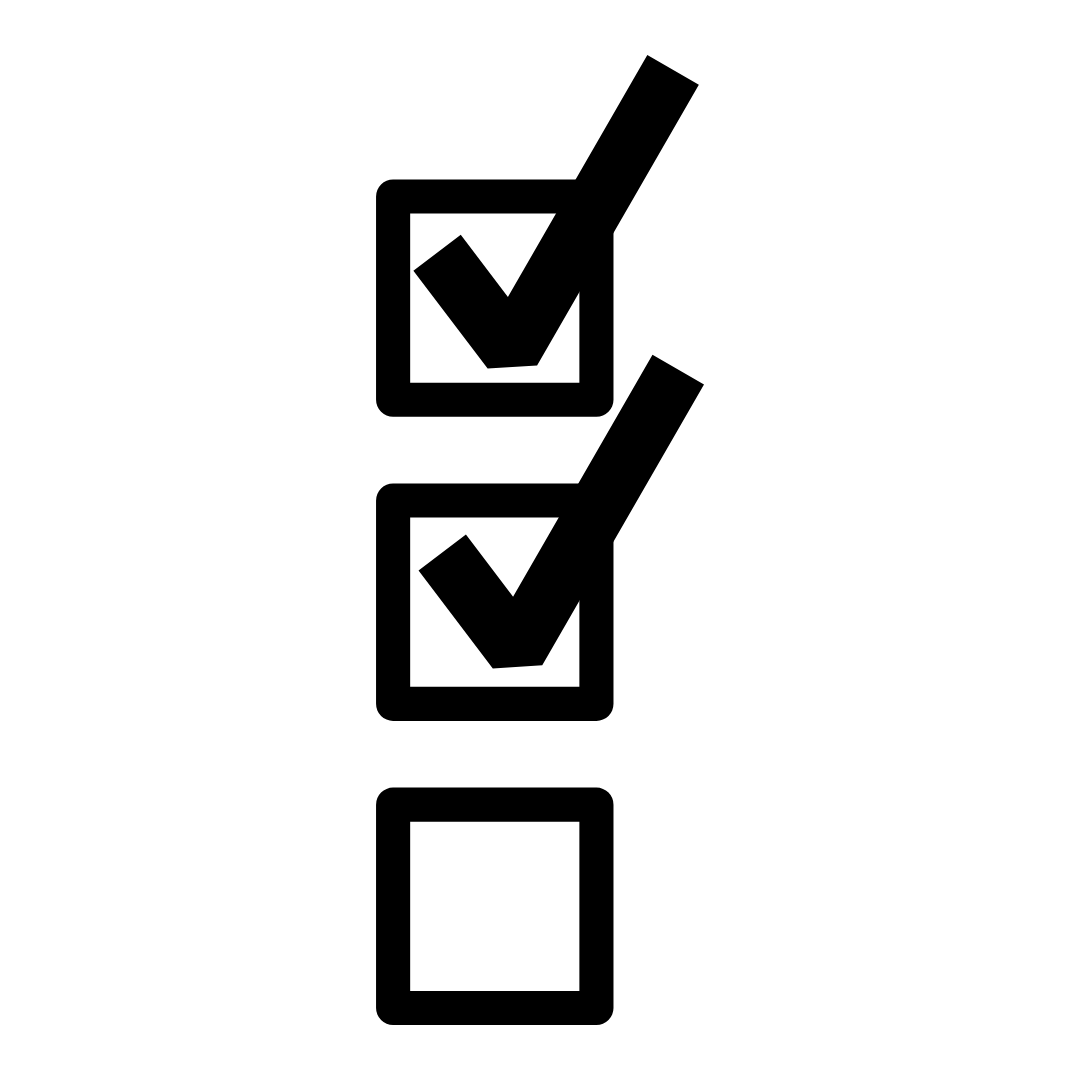 | 1. Download the 1HZH and 2NY7 structures in .pdb format from the Protein Data Bank (https://www.rcsb.org/) according to the following path:  \| *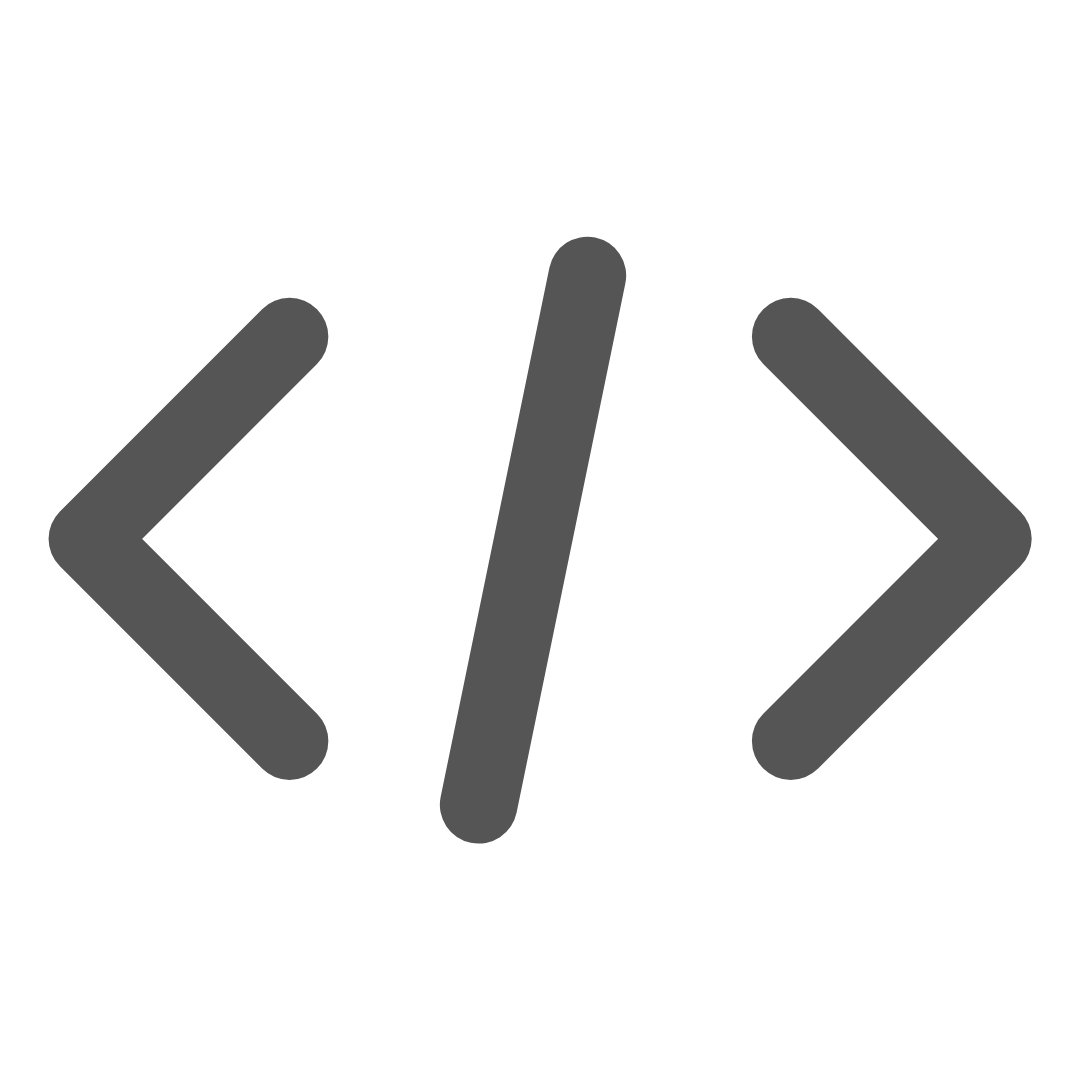* \| *3D structure search “code” > Download Files > PDB Format** \| \| --- \| --- \|   *See tutorial 1 if in doubt.   1. Open the Chimera interface from the shortcut generated on the desktop 2. Click on the “Open” menu in the “Home” tab, and select the folder where the 1HZH.pdb (antibody) file was saved, and double-click to open the file.  \| 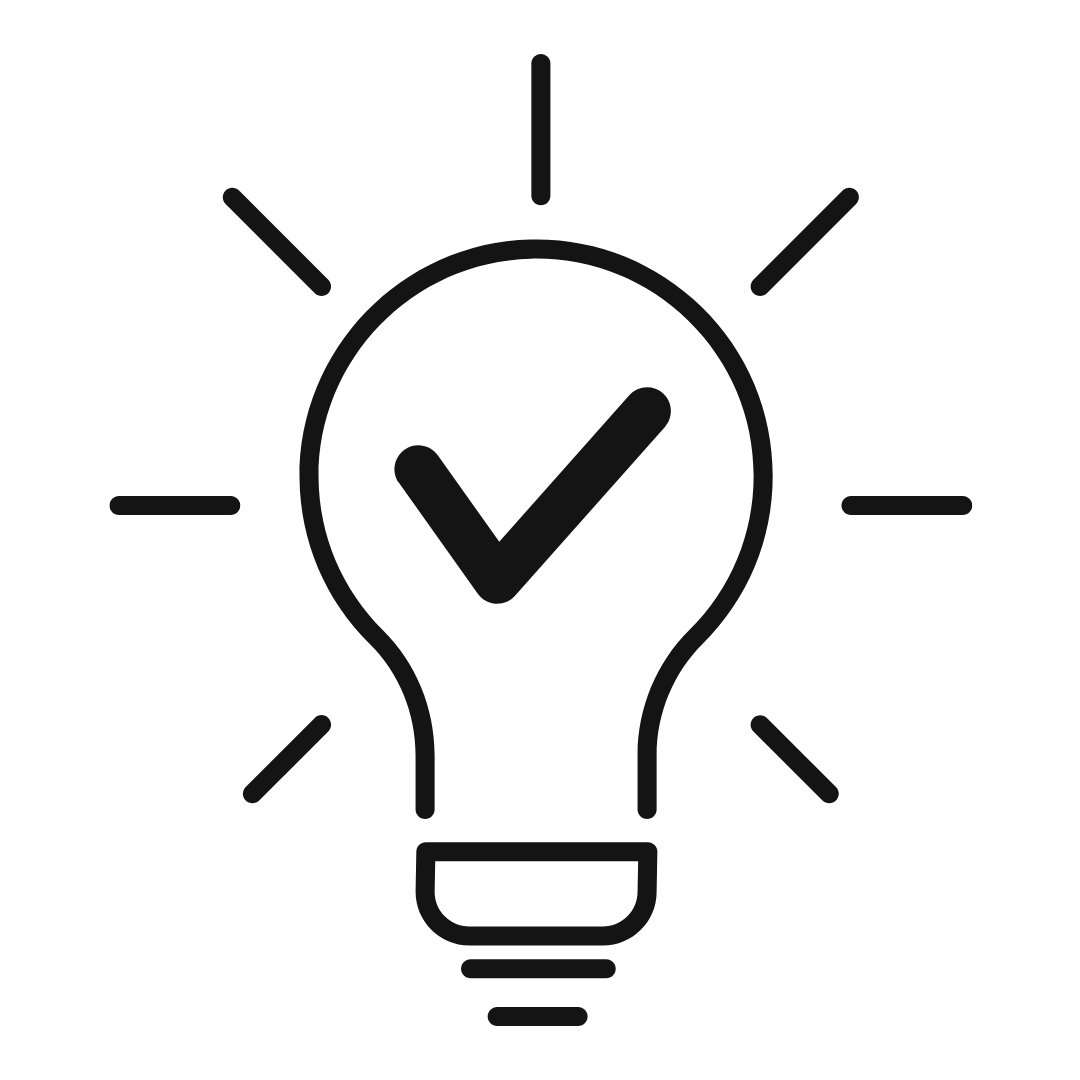 \| From this process, the antibody can be visualized in the Chimera X interface \| \| --- \| --- \|  1. When opening the structure in Chimera X, a tab will be generated on the right side of the interface. This tab contains a table with information about the chains present in the 1HZH structure, representative of the B12 antibody.   4.1. Selecting the H chain (representing one of the antibody's heavy chains) by clicking on the table will also highlight the selection in the structure. The same occurs for the K chain, corresponding to the antibody's second heavy chain, and for the L and M chains, which refer to the light chains.  4.2. To highlight the different chains, we can assign specific colors to them. For example, selecting chain H assigns the color purple through the following path:   \| *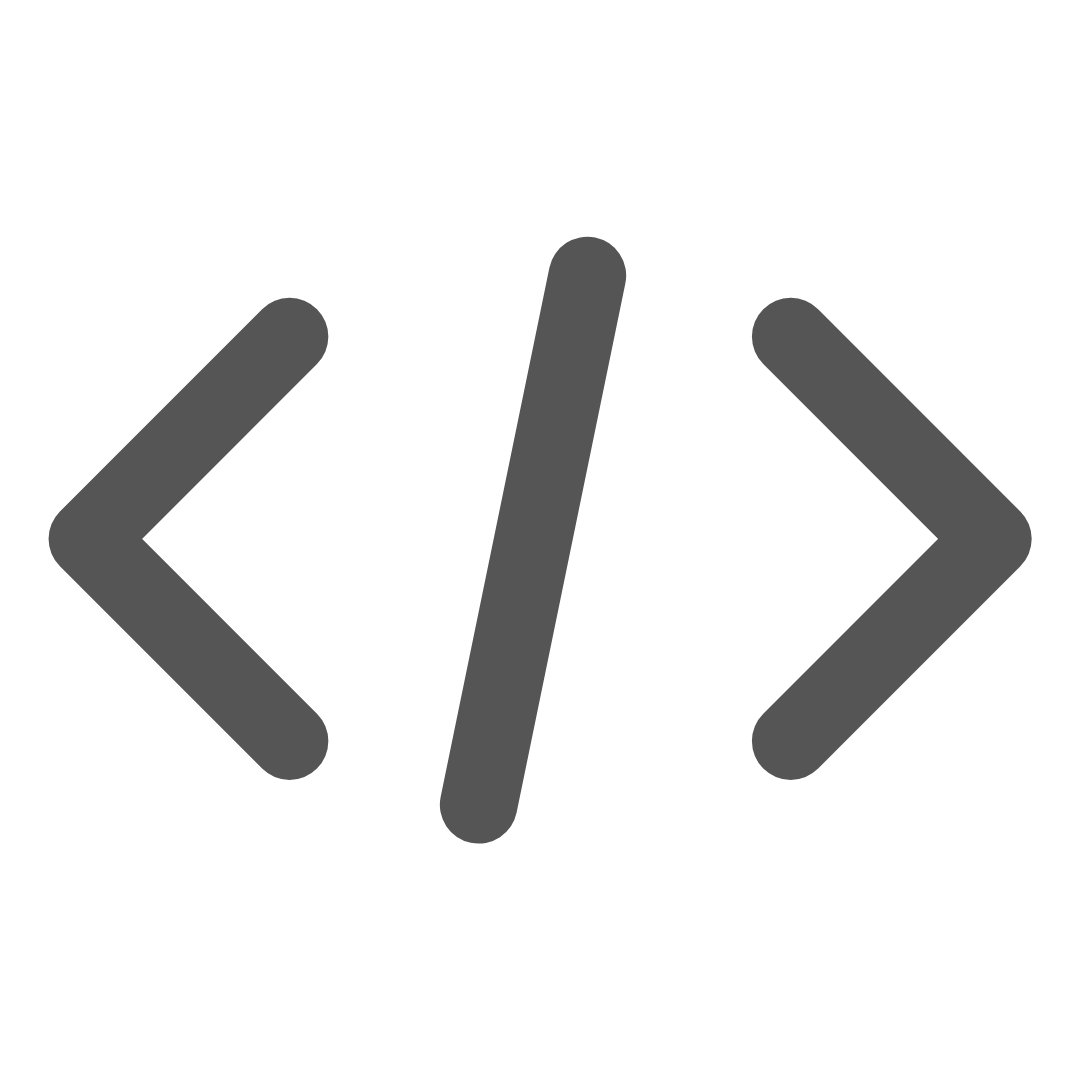* \| Right panel selection “H” > Taskbar > “Action” > “Color” > “Purple” (or user preferred color)* \| \| --- \| --- \|   *The same can be done for other chains.   1. To better visualize the structures, the “white background” and the graphic outline were configured as “silhouettes,” which can be done through the command below or as instructed in tutorial 1.  \| *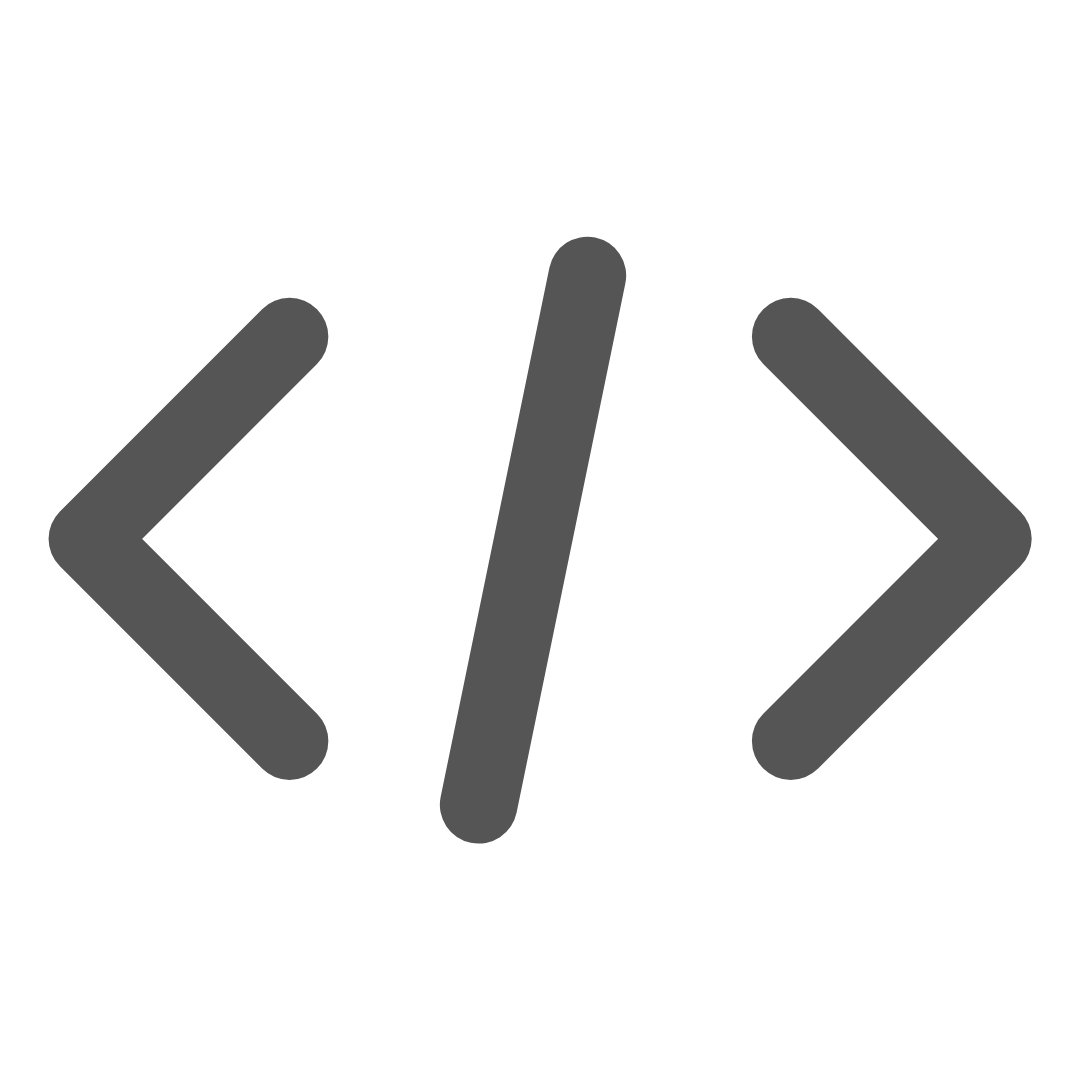* \| In the header, select “Graphics” > Background “White” > Lighting and Effects “Silhouettes” \| \| --- \| --- \| |
| --- | --- | --- | --- | --- | --- | --- | --- | --- | --- |

At the end of Step 1, the different antibody chains and their definitions within the structure can be verified according to the concepts initially discussed. Figure 2 represents the result of this initial analysis.


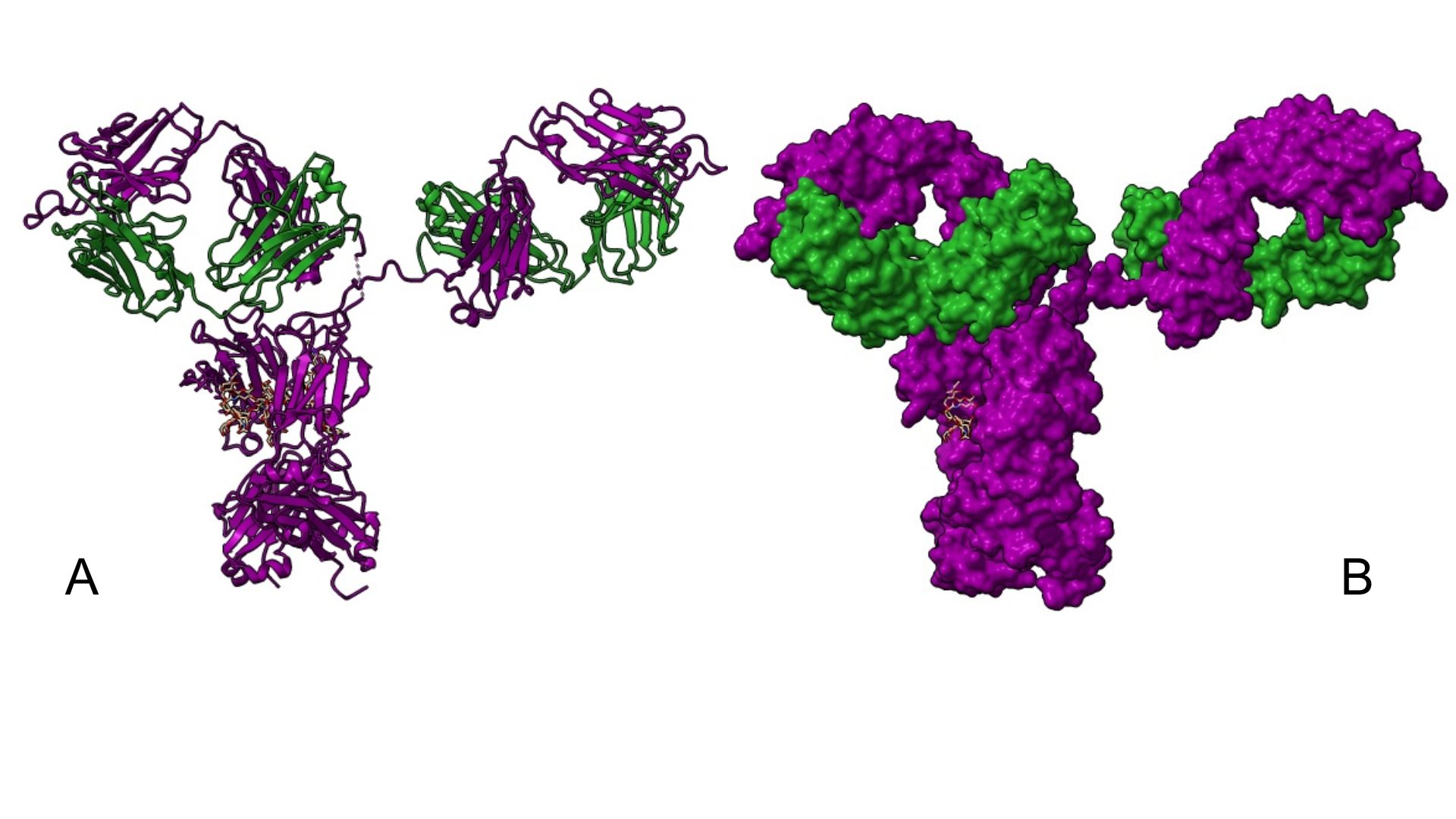


Figure 2. Representation of the heavy (purple) and light (green) chains of the 1HZH structure obtained from the Protein Data Bank, corresponding to the B12 antibody. In A, the structure is represented in the “Cartoon” configuration, and in B, in the “Surface” configuration (Header > Molecule Display > Surface: “Show” - see Tutorial 1).

Source: Compiled by the authors

- Step 2:

| 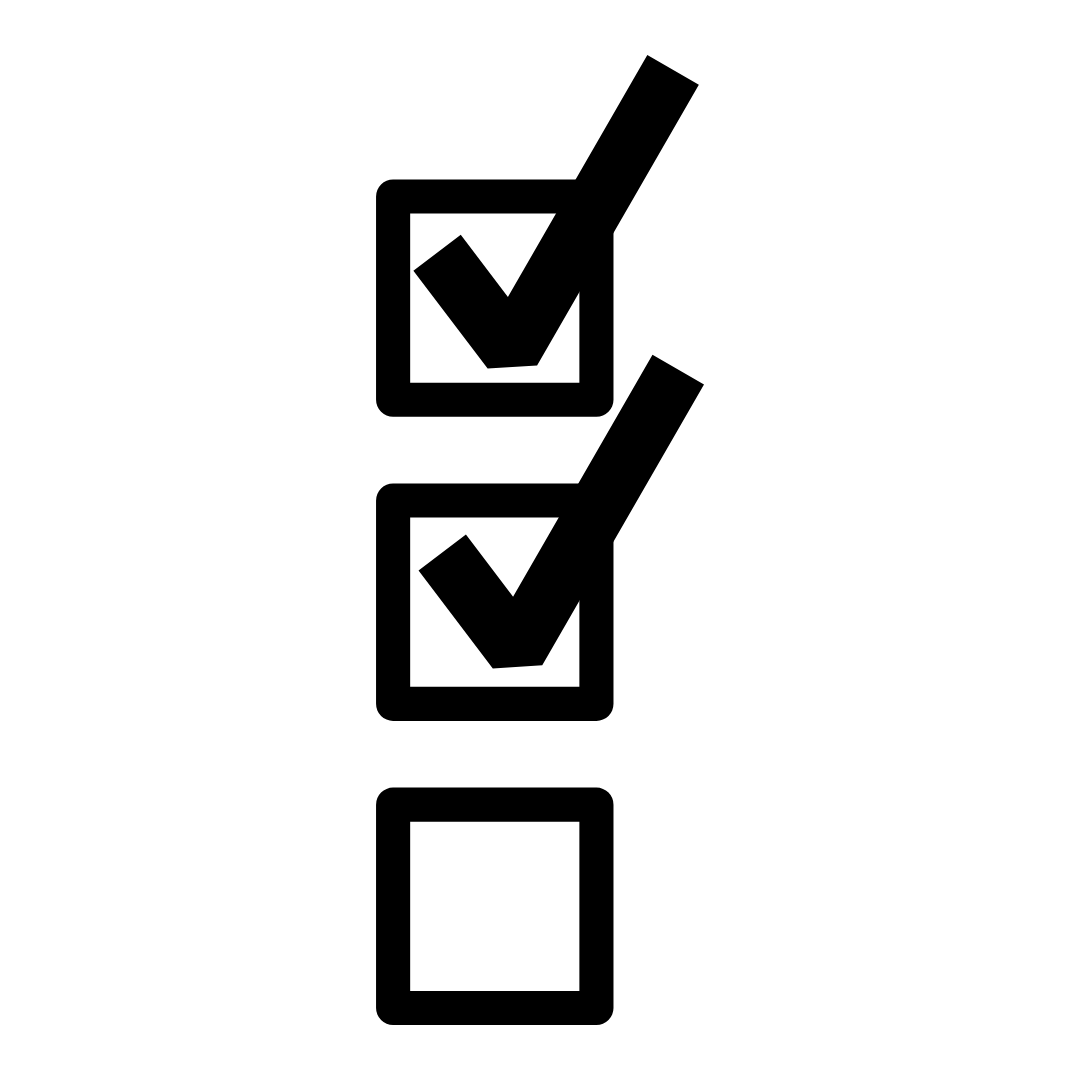 | 1. To begin understanding and evaluating the interaction between the antigen and the B12 antibody, the PDB: 2NY7 structure must be opened in the Chimera X interface through the path:  \| *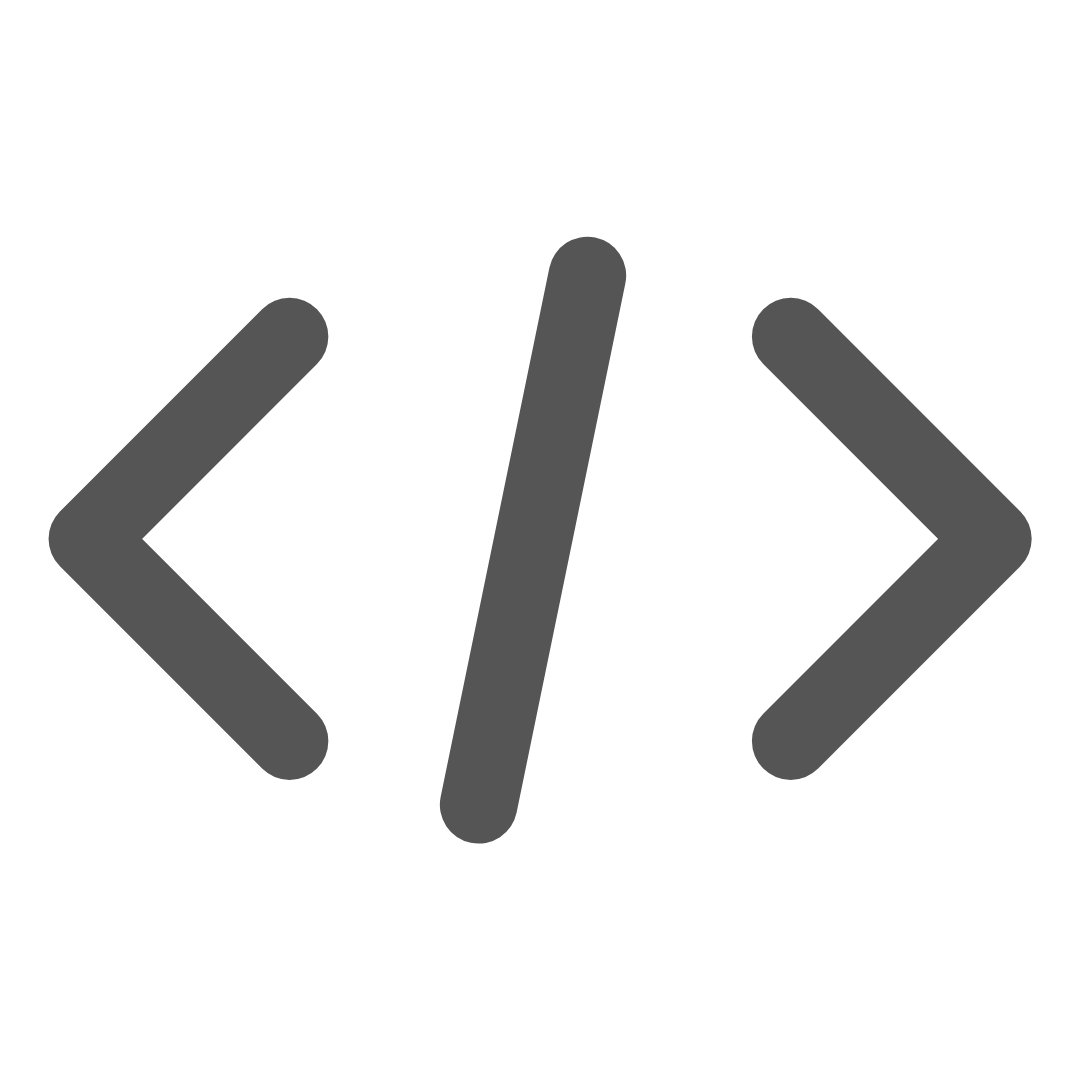* \| *Click on the “Open” menu in the “Home” tab. Select the folder where the 2NY7.pdb (antigen) file was saved and double-click to open it.* \| \| --- \| --- \|  \| 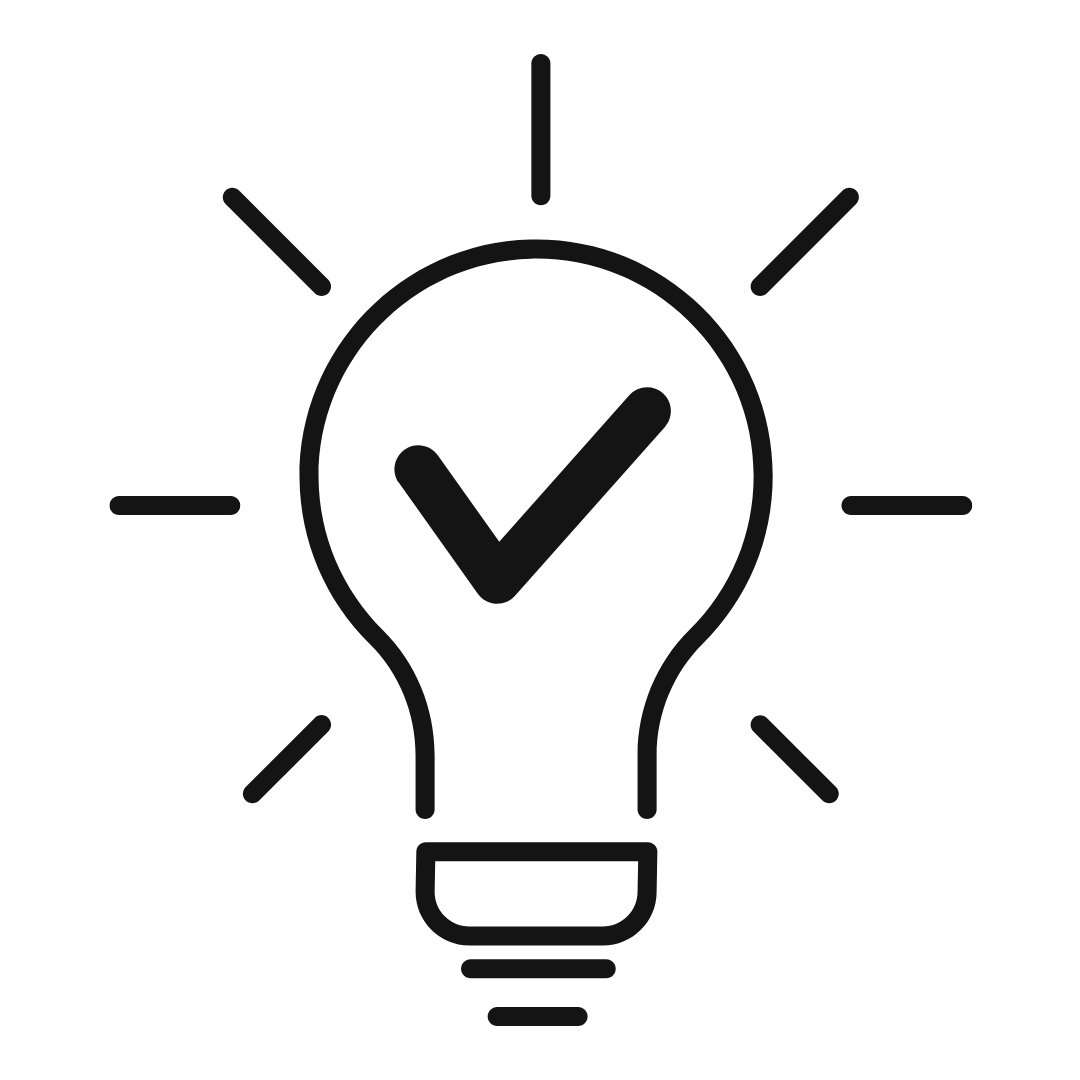 \| This structure corresponds to the binding region of the gp120 protein with the antigen-binding fragment (Fab) of the B12 antibody. \| \| --- \| --- \|  1. Perform the superimposition (matchmaker) of the structure containing the Fab interacting with the gp120 antigen (PDB code: 2NY7) on the structure of the complete antibody (PDB code: 1HZH), using the following path:  \| *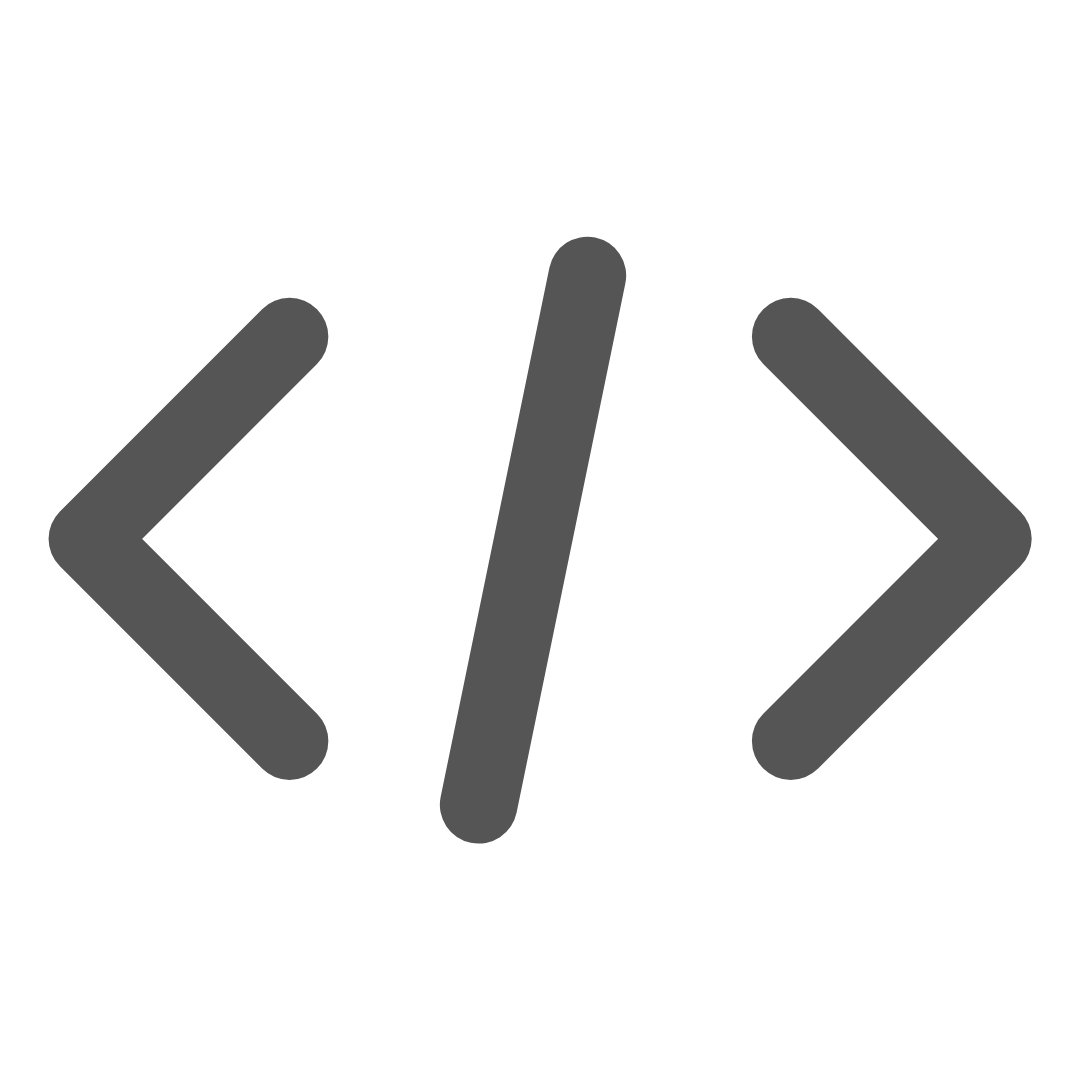* \| Click on “Tools” on the taskbar > “Structure Analysis” > “Matchmaker”  A dialog box will open. In it, select the 1HZH structure as the reference and the 2NY7 structure as the one to be aligned. No other settings are necessary; just click “OK.” \| \| --- \| --- \|  \| 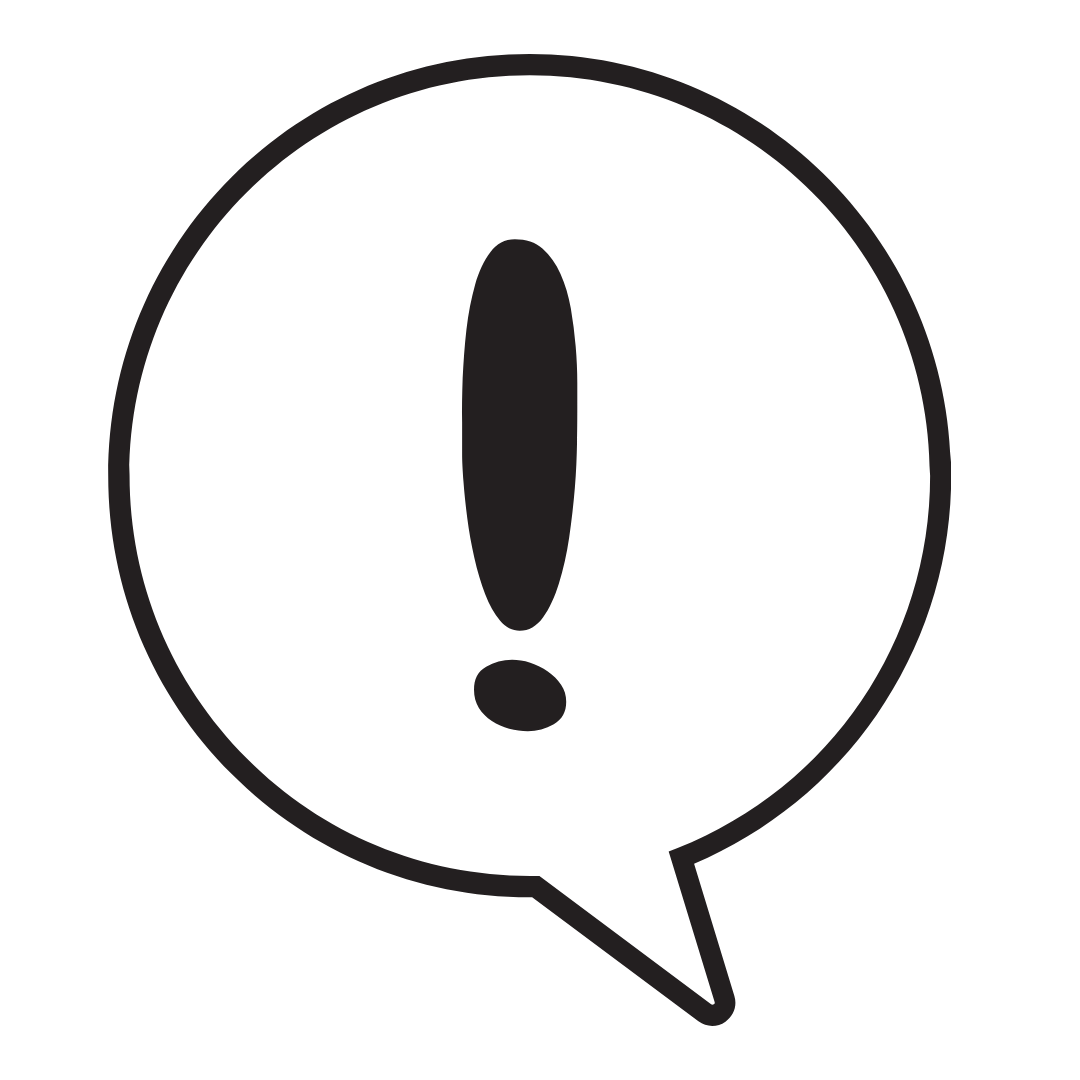 \| The Matchmaker function performs a structural alignment, considering a structure as a reference. That is, the structure will remain positioned in the initial configuration, while the structure to be superimposed will align with the region of greatest similarity in the reference structure, obeying its configuration. \| \| --- \| --- \| |
| --- | --- | --- | --- | --- | --- | --- | --- | --- | --- |

In Step 2, you can practice an important resource provided by Chimera X, the Matchmaker. This approach is essential for different bioinformatics analyses, as it allows for comparing structures and identifying similarities and differences. Therefore, it is extremely important to know how to use it. Figure 3 shows how the analysis looks after using this resource and the others covered in step 2.


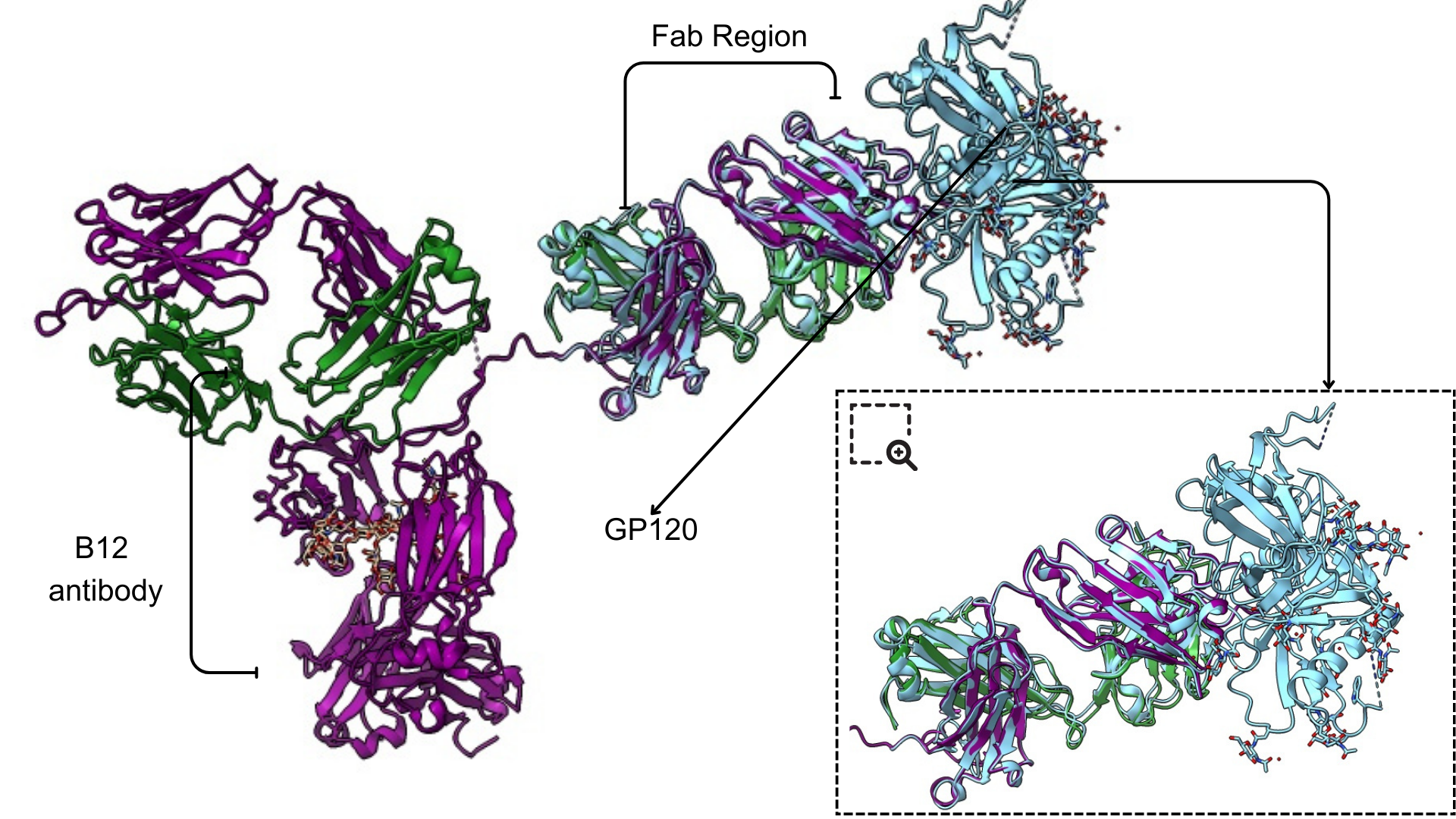


Figure 3. Representation of the structure of the B12 antibody from the PDB code 1HZH, with the structure of the 2NY7 code superimposed on it (in blue). The 2NY7 structure has the Fab fragment and the gp120 antigen, thus identifying the region of the Fab fragment of 2NY7 superimposed on the complete antibody (1HZH) and observing the positioning of the antigen region that binds to the Fab fragment.

Source: Compiled by the authors

### **4.2. Concept 2: Interaction between the antigen-antibody complex**

Antigen-antibody interaction occurs when an antigen, such as a protein from a pathogen, enters the body and is recognized by the variable regions of the antibody. These regions, located at the ends of the heavy and light chains of the antibody (variable region), form a complementary binding site for the antigen, similar to a key that fits into a lock. Once the antibody binds to the antigen, the antigen-antibody complex is formed, which can trigger various immune responses (6).

In this sense, the binding sites between the antibody and the antigen are specific sites of interaction between them. In the antibody, there is the paratope, which is the region that binds directly to the antigen and is located in the variable regions. The antigens' surface region that binds to antibodies is the epitope, a specific region that determines the selectivity in this identification process (3).

From the procedures performed in Concept 1 (Steps 1 and 2), it is possible to analyze the region of interaction between the antigen and the antibody. In this sense, follow the step-by-step developed for the execution of analyses of the binding regions of the monoclonal antibody B12 and gp120

- Step 3:

| 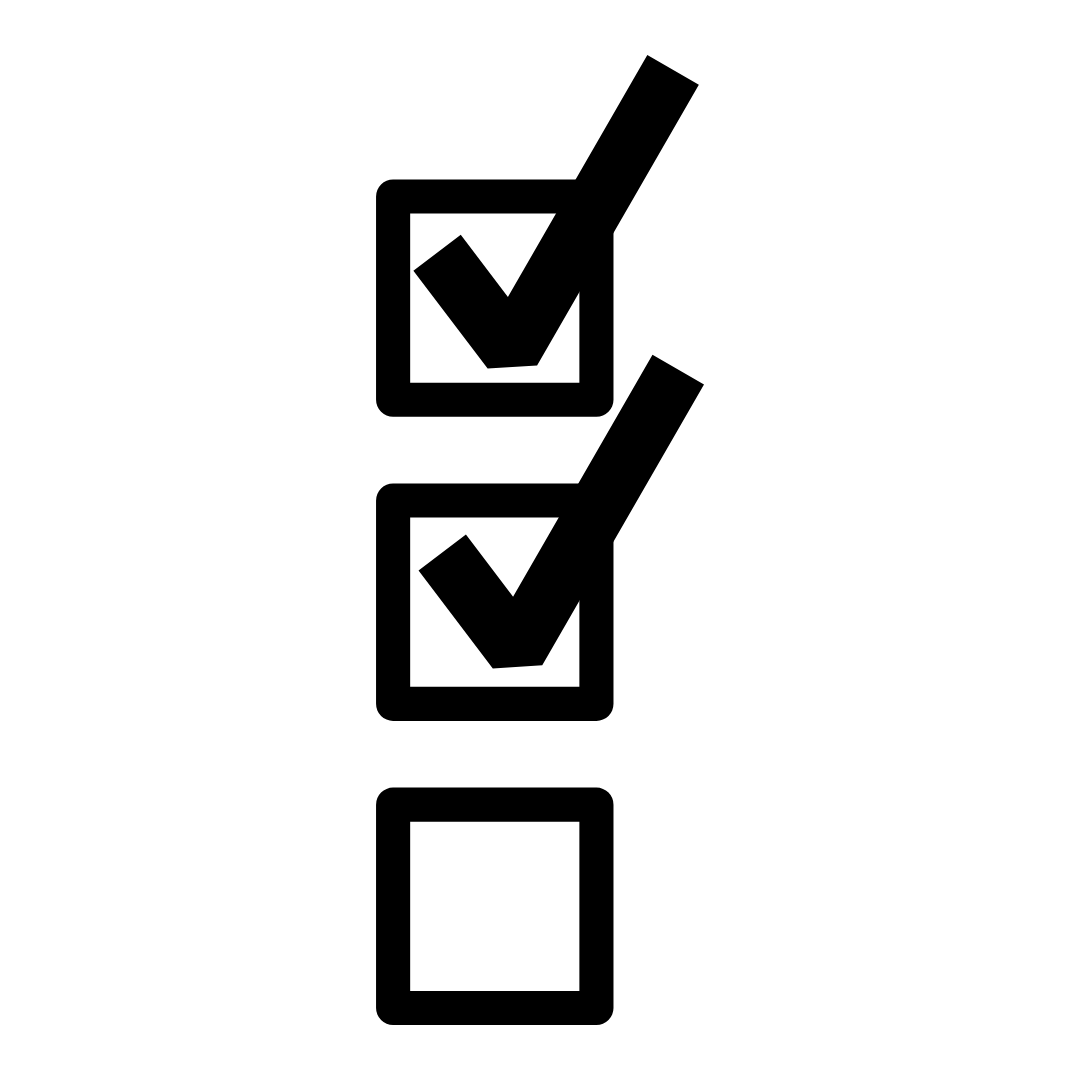 | 1. To generate new insights into the interaction between the antigen and the antibody, the antigen-binding region (Fab) will be isolated using the “Model Panel” selection menu, leaving only the 2NY7 structure visible. In addition, according to the steps previously performed for the complete antibody, different colors will be assigned to the Fab fragment and the antigen to better understand them. 2. The information on the 2NY7 pdb chains can be viewed in the tab on the right. The Fab light and heavy chains are named L and H, respectively, and the chain corresponding to the glycoprotein GP120 is named G. These can be selected by clicking on each chain. Thus, different colors can be determined for them. To open the selection menu:  \| *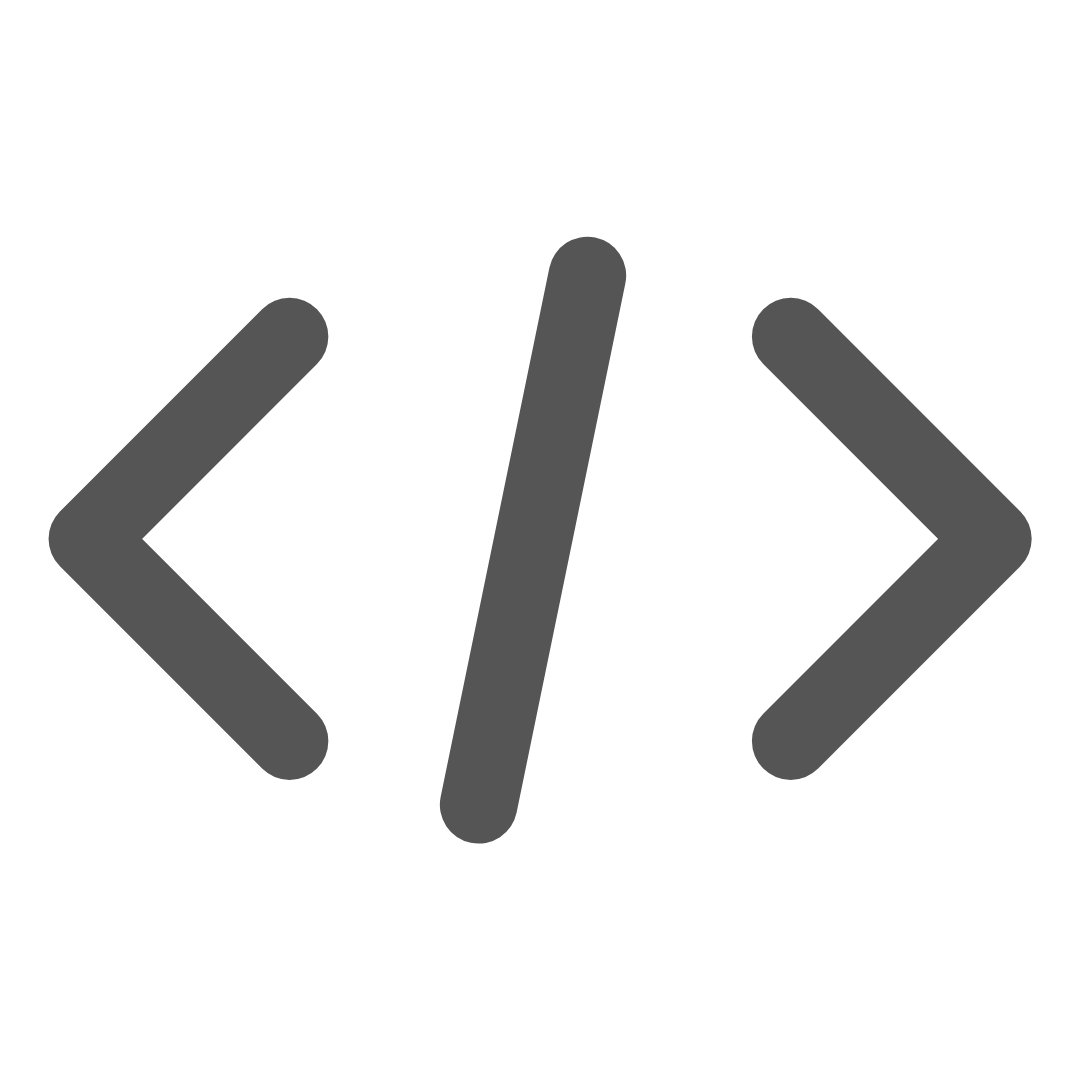* \| In the taskbar “Tools” > Select “Model” > Deselect the 1HZH view \| \| --- \| --- \| |
| --- | --- | --- | --- |

The procedures covered in Step 3 allow clear visualization of the specific interactions between the antigen and the antibody Fab fragment, facilitating the analysis of binding regions and the detailed understanding of the conformation of the antigen-antibody complex, as seen in Figure 4.


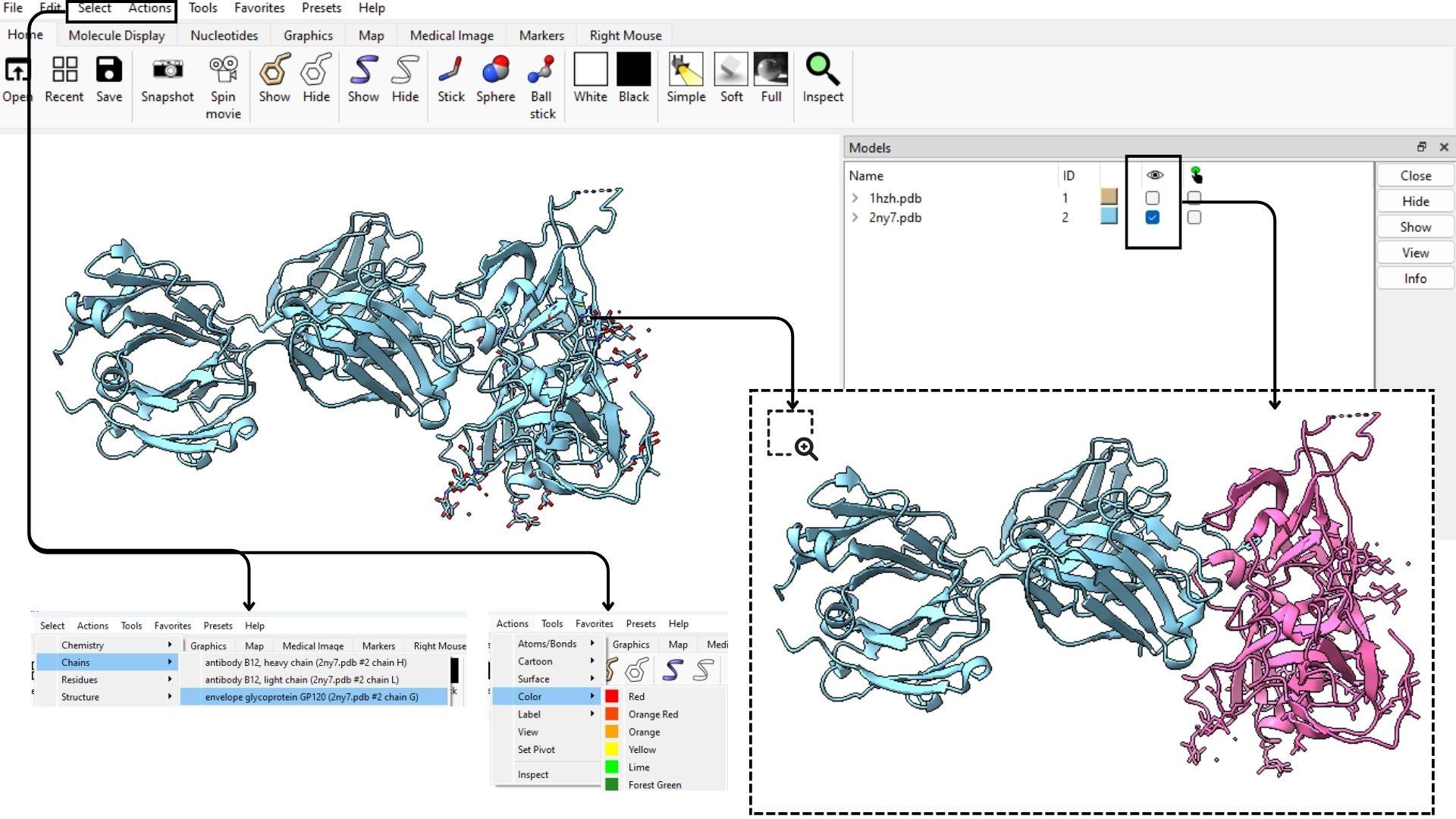


Figure 4. Representation of the 2ny7 structure containing the Fab fragment and part of the gp120 antigen, showing the selection panel that allowed only the structure of interest to be displayed. Highlighted, the glycoprotein's color was changed to understand the interaction better. Performed through the command (Taskbar: Select > Chains > envelope glycoprotein GP120 (Chain G)/ Taskbar: Action > Color > Hot pink/ Taskbar: Select > clear)

Source: Compiled by the authors

### **4.3. Concept 3: Antigen-antibody binding analyses**

The binding regions between the antibody and the antigen are important for the specificity and efficacy of the immune response. These interactions occur through specific amino acid residues in the antibodies' paratopes and the antigens' epitopes, forming a molecular binding region (7).

The antigen-antibody complex is formed through different interactions, including hydrogen bonds between a positive hydrogen atom and an electronegative atom, such as oxygen and nitrogen, stabilizing the interaction between complex members (8). In this sense, the previously introduced concepts allow an understanding of the importance of a detailed analysis of how recognition and binding between antibodies and antigens occur. Thus, the final stages of the tutorial will focus on undercovering and visualizing the interactions of the antigen-antibody complex.

- Step 4

| 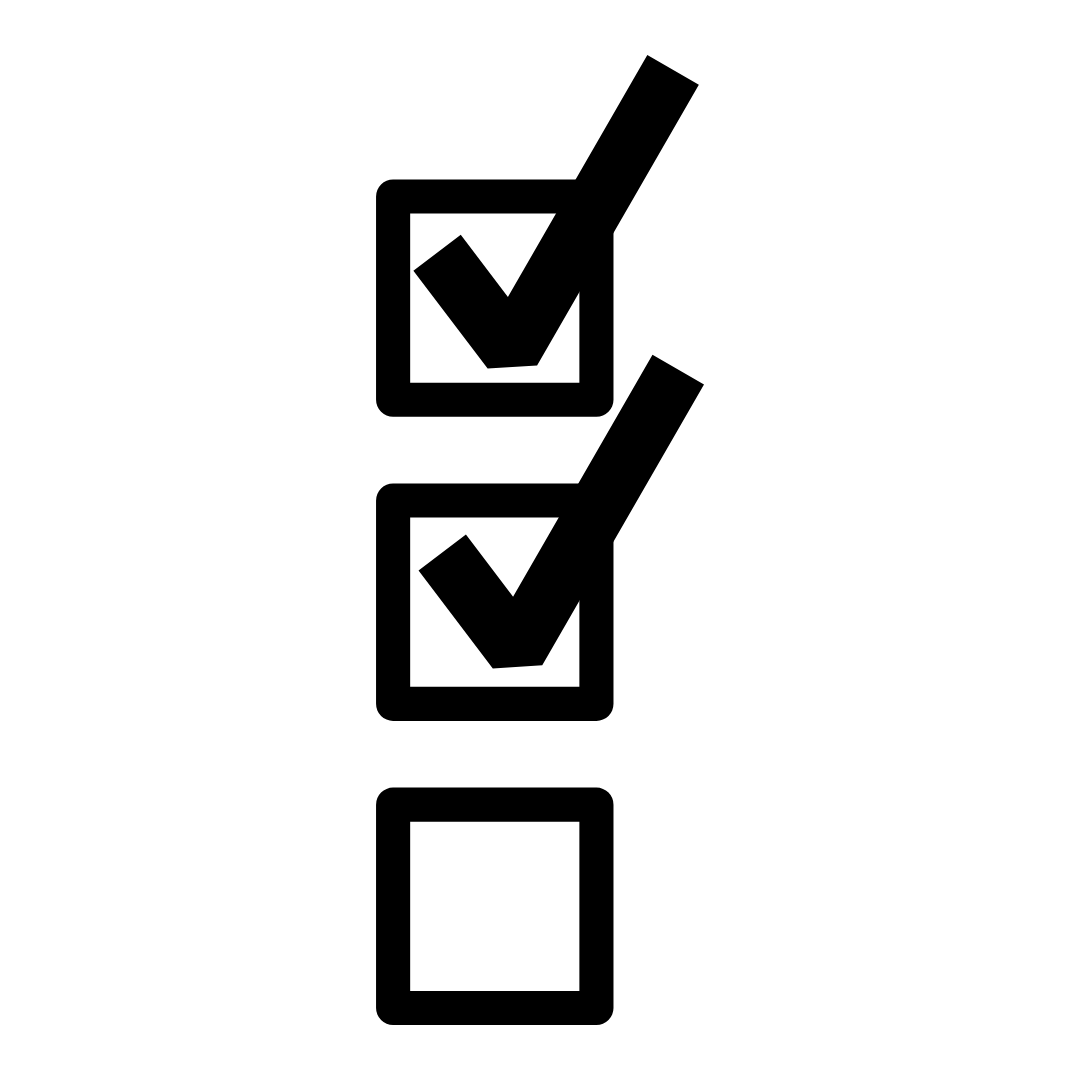 | 1. To better visualize the binding regions between the antibody and the antigen, the way the antigen is displayed has been modified, assigning a surface to it. To do this, it must be selected using the command:  \| *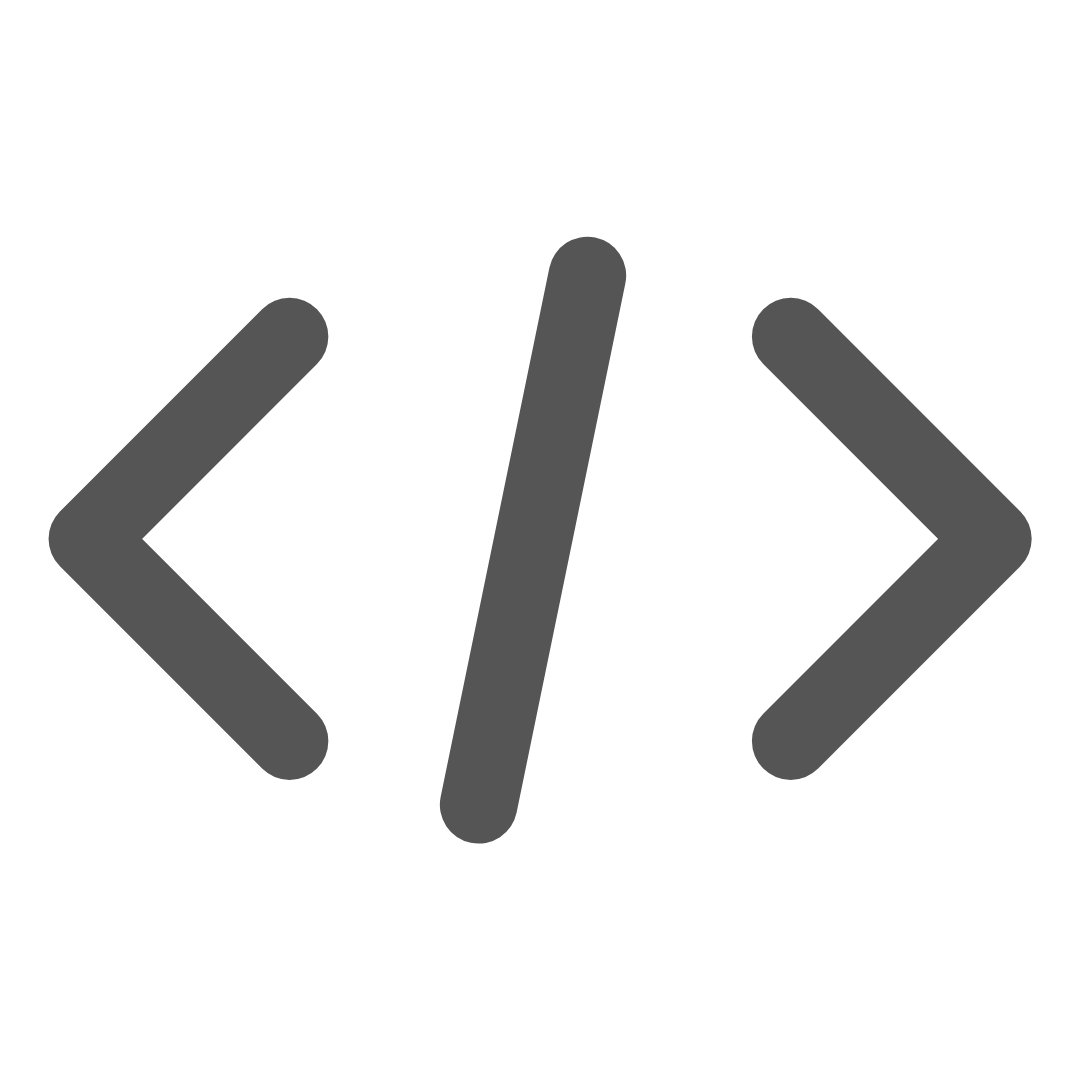* \| *Taskbar: Select > Chains > envelope glycoprotein GP120 (Chain G). Then the surface is assigned by clicking on Molecule display in the header and selecting “show” for “Surfaces”.* \| \| --- \| --- \|  1. This will give us different views on GP120 and the anti-HIV antibody. The region where the antibody interacts with the antigen can be highlighted using the command line through the following script:  \| *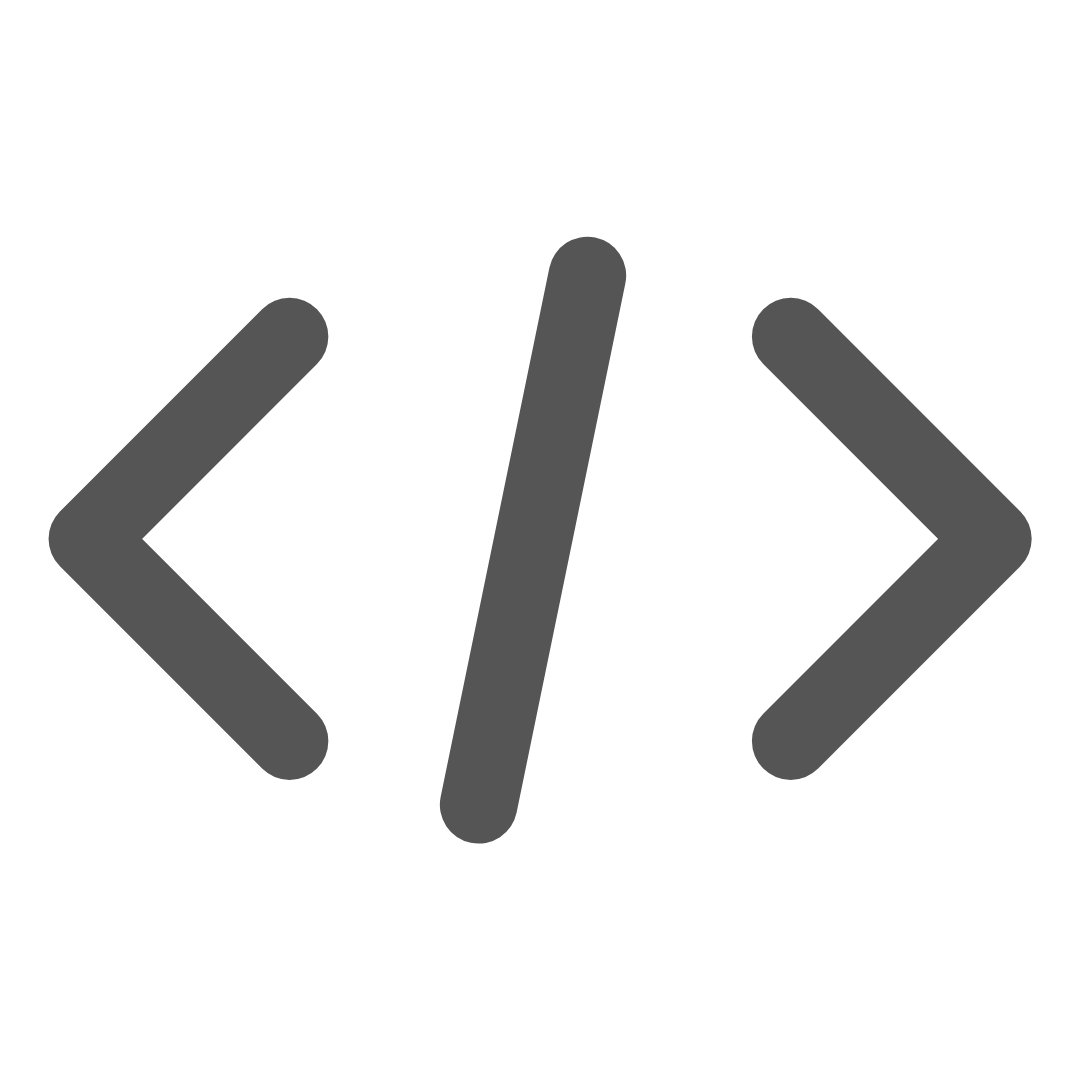* \| *“ select / H: 25-34, 51-57, 94-102”* \| \| --- \| --- \|   *this numbers were estimated for the specific example target in this tutorial     1. This way, we will have the region of the Fab fragment that interacts with the selected antigen. That is, the paratope of this antibody has been selected. From this selection, a different color can be assigned to highlight this region using the commands on the taskbar. |
| --- | --- | --- | --- | --- | --- |

Concept 3 brings a depth of knowledge acquired in Concept 2, initially focusing on the binding region that interacts with the antigen (Figure 5). This approach, in addition to allowing the identification of the paratope, serves as a preparation for advancing in more complex structural bioinformatics studies.

*
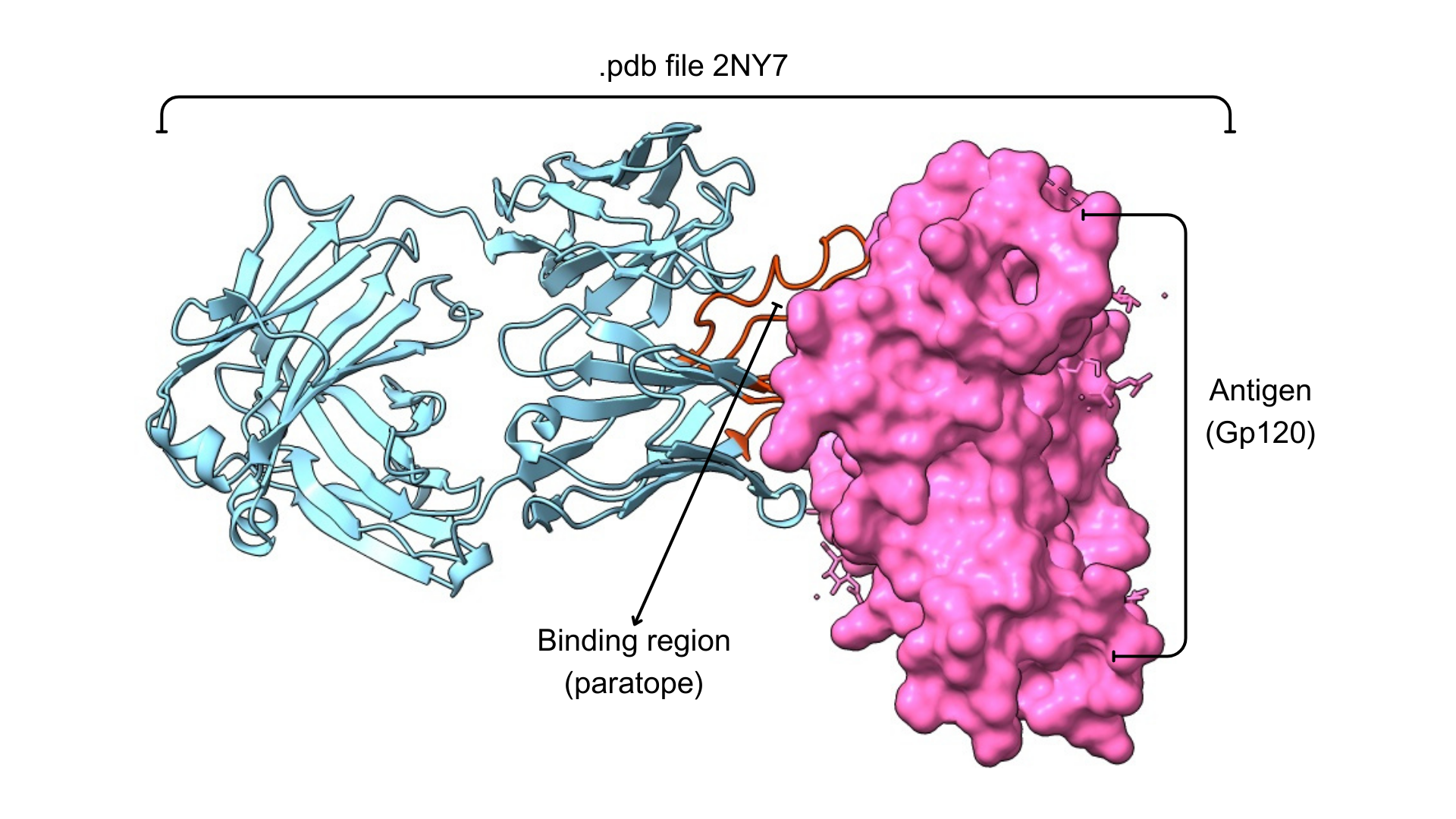
*

*Figure 5. From Step 4, a representation of the chimera interface with the Fab fragment interacting with the GP120 antigen was made. The antigen was assigned pink, and a surface representation was given. The antibody's interaction region with the antigen (paratope) is highlighted in orange.*

Source: Compiled by the authors

- Step 5

| 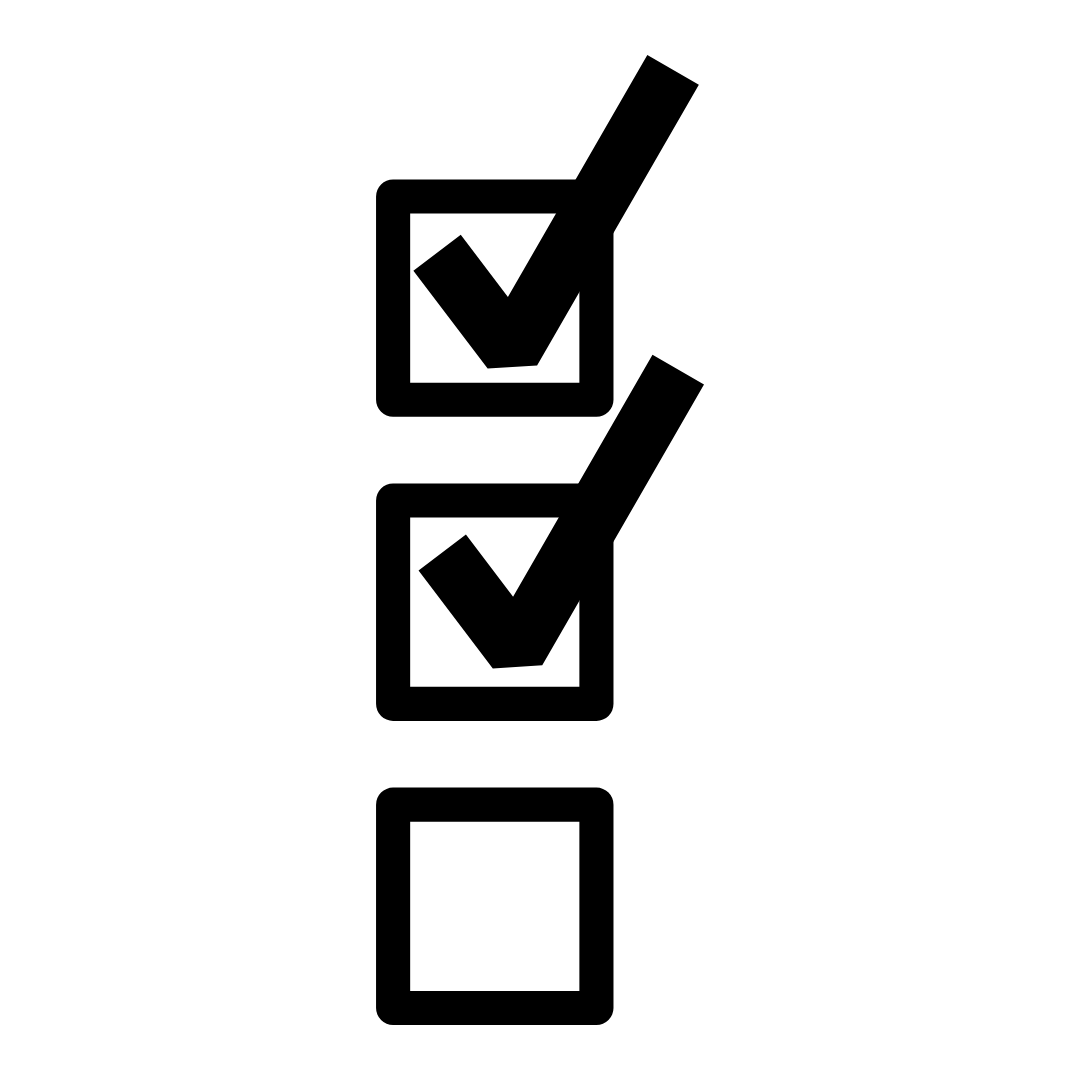 | 1. To better understand how interactions between antigen and antibody occur, we will analyze the formation of hydrogen bonds from the Chimera X interface. This feature can be performed through the following path:  \| *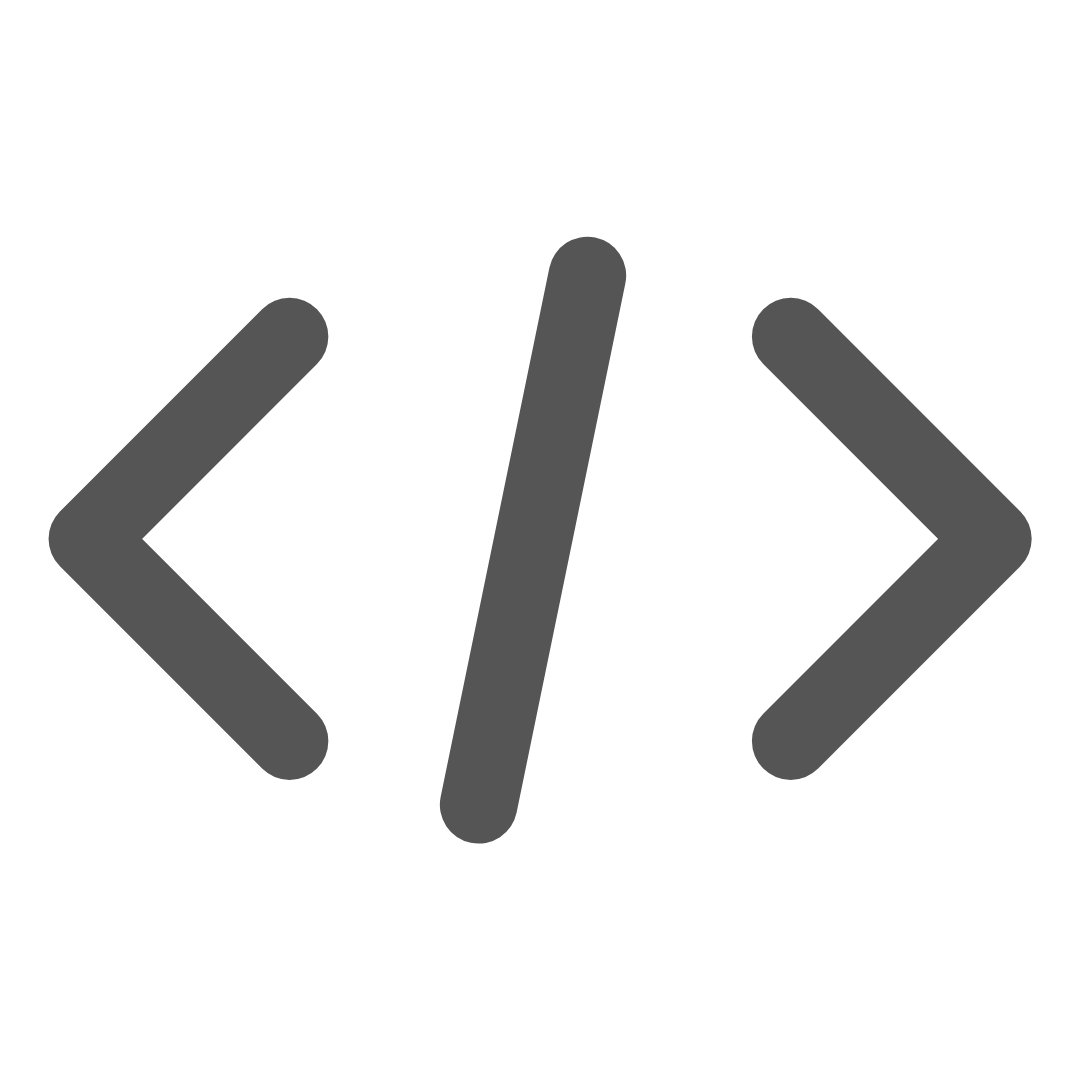* \| *Repeat the command: select / H: 25-34, 51-57, 94-102 for paratope selection. In the Molecule display tab of the header, select “H-bonds” within the “Analysis” group.* \| \| --- \| --- \|  1. From this command, the hydrogen bonds formed between the paratope and the antigen will appear as blue dotted lines. When you zoom in on the image and click on a line with the mouse, information about the bond, such as which amino acids are involved, will appear.  \| 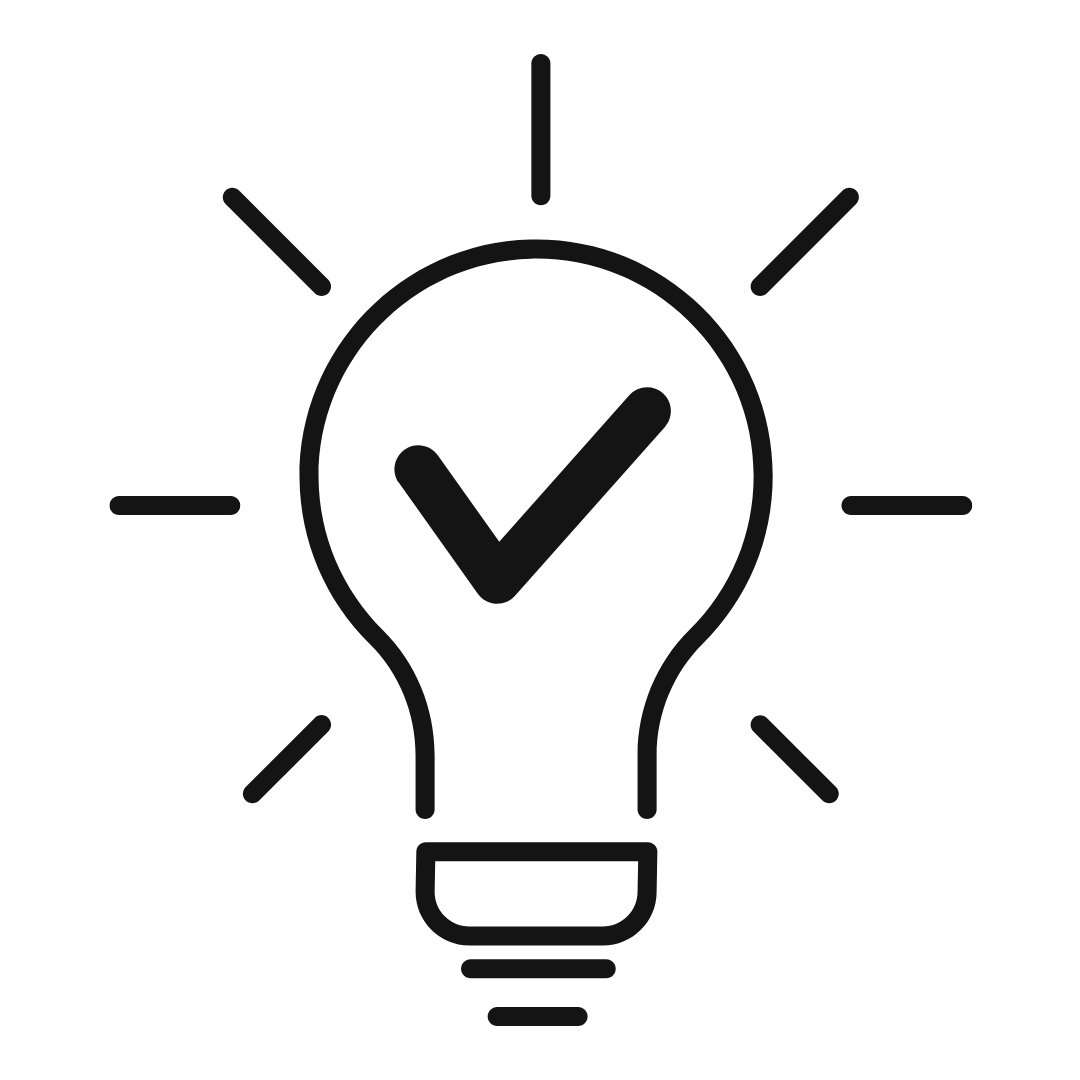 \| It is noted that the antibody has bonds with the antigen (intermolecular) but also with itself (intramolecular). Intramolecular bonds are also important to conformation maintenance, especially considering the paratope region. \| \| --- \| --- \|  1. You can finish your analysis by saving the generated images, as demonstrated in Tutorial 1. |
| --- | --- | --- | --- | --- | --- |

The tutorial culminates in Step 5, where we finalize the analysis of antigen-antibody interactions by examining hydrogen bond formation using the Chimera X interface (Figure 6). Through its detailed steps, this tutorial provides an in-depth understanding of antigen-antibody interactions, highlighting the importance of binding regions, specific interactions, and the intermolecular forces that stabilize them. With this foundation, it is possible to advance in immunological analyses of biomolecules and improve the practice of structural bioinformatics techniques.

*
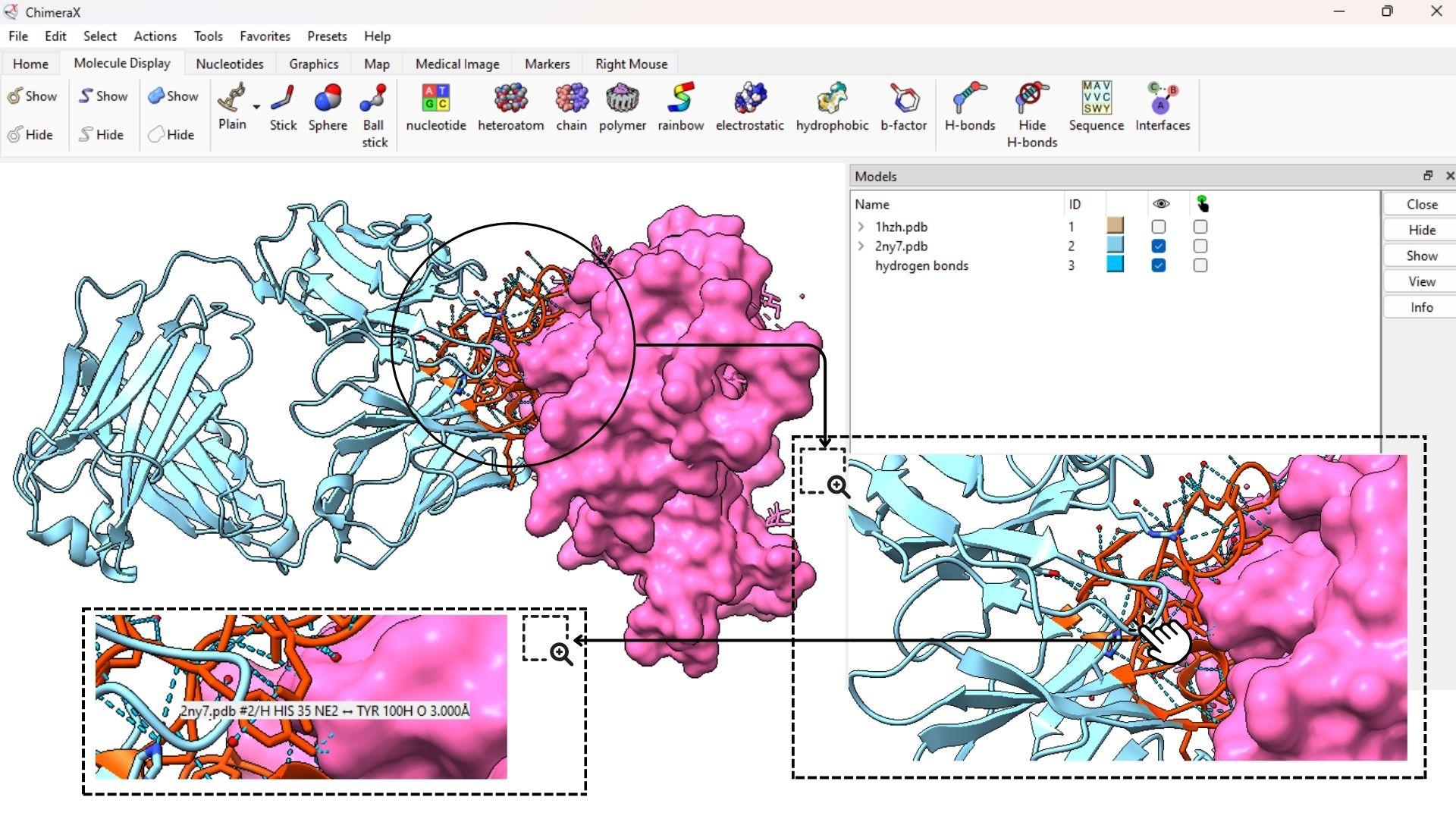
*

*Figure 6. Representation of the hydrogen bonds that occur in the region of interaction between the antibody and the antigen, generated by the “H-bonds” resource in “Analysis” in the header. Blue dotted lines highlight the bonds, and the specification of one of these bonds can be seen by clicking the mouse. This specification shows the amino acids involved and the chains and distance. The figure details the interaction between a Histidine (HIS) and a Tyrosine (TYR).*

Source: Compiled by the authors

## **QUESTIONNAIRE**

A questionnaire was developed to evaluate the learning process with this tutorial. Therefore, it is suggested that it be applied before and after the Tutorial, with the objective of the student and teacher being to perceive the knowledge acquired through it and adapt it for the class.

1. What is the structure of a monoclonal antibody?
2. Explain the concept of antigen-antibody interaction and give examples of types of binding?
3. What is the function of the glycoprotein gp120 in the process of HIV infection?
4. The structures 1HZH and 2NY7 are representations of which biomolecules in the context of the tutorial and what are their main chains?
5. Explain the function of the Matchmaker in Chimera X and its usefulness.
6. What is the importance of the Protein Data Bank?
7. What is the name of the region of the antibody that interacts with the antigen? And vice versa
8. Suggest a reason for studying interactions between antigen and antibody
9. Name a feature of the Chimera interface that allows the study of interactions;
10. Why do you think visualization of structures and their interactions is important within immunology? What applications do you see for this?

## **CONCLUSION**

Upon completing this tutorial on structural bioinformatics in immunology using Chimera X, students are expected to have acquired essential practical and theoretical skills to analyze interactions between antigens and antibodies. This knowledge includes the ability to visualize and manipulate three-dimensional structures of proteins, identify binding regions, and understand specific molecular interactions, such as hydrogen bonds.

Furthermore, it is worth noting that the knowledge acquired in the tutorial provides a starting point for studies on mastering bioinformatics techniques. Understanding these techniques not only contributes to the understanding of immunological mechanisms but also provides insights for advancing research in immunology.

## **REFERENCES**

1. Schwartz SA, Nair MPN. Current concepts in human immunodeficiency virus infection and AIDS. Clin Diagn Lab Immunol [Internet]. 1999 [citado 13 de junho de 2024];6(3):295–305. Disponível em: https://pubmed.ncbi.nlm.nih.gov/10225826/

2. Timofeeva A, Sedykh S, Nevinsky G. Post-immune antibodies in HIV-1 infection in the context of vaccine development: A variety of biological functions and catalytic activities. Vaccines (Basel) [Internet]. 2022 [citado 13 de junho de 2024];10(3):384. Disponível em: http://dx.doi.org/10.3390/vaccines10030384

3. Janeway CA Jr, Travers P, Walport M, Shlomchik MJ. The structure of a typical antibody molecule. Londres, England: Garland Science; 2001.

4. Schroeder HW Jr, Cavacini L. Structure and function of immunoglobulins. J Allergy Clin Immunol [Internet]. 2010;125(2):S41–52. Disponível em: http://dx.doi.org/10.1016/j.jaci.2009.09.046

5. Chaplin DD. Overview of the immune response. J Allergy Clin Immunol [Internet]. 2010;125(2):S3–23. Disponível em: http://dx.doi.org/10.1016/j.jaci.2009.12.980

6. Janeway CA Jr, Travers P, Walport M, Shlomchik MJ. The interaction of the antibody molecule with specific antigen. Londres, England: Garland Science; 2001.

7. Peng H-P, Lee KH, Jian J-W, Yang A-S. Origins of specificity and affinity in antibody–protein interactions. Proc Natl Acad Sci U S A [Internet]. 2014 [citado 13 de junho de 2024];111(26):E2656. Disponível em: http://dx.doi.org/10.1073/pnas.1401131111

8. Ramaraj T, Angel T, Dratz EA, Jesaitis AJ, Mumey B. Antigen–antibody interface properties: Composition, residue interactions, and features of 53 non-redundant structures. Biochim Biophys Acta Proteins Proteom [Internet]. 2012;1824(3):520–32. Disponível em: http://dx.doi.org/10.1016/j.bbapap.2011.12.007
