# Supplementary material for: Chimera X Interface to Enhance Understanding in Biochemistry and Immunology
Source: Biochem Mol Biol Educ. 2025 Nov 14;54(1):92–102. doi: 10.1002/bmb.70025 (PMC12877970; doi:10.1002/bmb.70025)
Supplement: Supplementary file 3 — Supporting Information 3. Tutorial 3—“Chimera X: Structural aspects in biochemistry.” [file BMB-54-92-s001.docx]

**TUTORIAL 3 CHIMERA X: STRUCTURAL ASPECTS IN BIOCHEMISTRY**

DALPIAZ, GIOVANA.¹,

KROHN, MURIEL SCHILING.¹,

ANJOS, ANDRÉ DA SILVA.¹,

MEIRELES, MARIANA R.

¹Universidade do Vale do Rio dos Sinos

**INDEX**

[**1. INTRODUCTION 1**](#_bcjo4sugefup)

[**1. OBJECTIVES 1**](#_kwhp8oq3uke6)

[**3. THEORETICAL BASIS 2**](#_cfzrdrc28o1u)

[**4. METHODOLOGICAL PROCEDURES 2**](#_htk8ax8aw7rj)

[**4.1. Concept 1: Electrostatic potential 3**](#_dc59owiuvevr)

[**4.2. Concept 2: Hydrophobicity 6**](#_oilui2xxtqeh)

[**5. QUESTIONNAIRE 8**](#_qznyxd7fpejt)

[**6. CONCLUSION 8**](#_jmkpbkgc1dbw)

[**REFERENCES 9**](#_bo8xxhwc89hh)

## **INTRODUCTION**

This tutorial will demonstrate approaches to structural bioinformatics techniques in Chimera X (version 1.7.1), relating them to immunology concepts. For this purpose, a case of a monoclonal antibody B12 against HIV and the glycoprotein gp120 antigen will be applied. The concepts will be addressed before each step to understand the procedures better. Later, as a theoretical basis, the role of gp120 and the antibody in the immune response to infection caused by HIV will be elucidated. Thus, executing the steps provided in the tutorial will be possible based on the theoretical understanding and established instructions.

## **OBJECTIVES**

This structural bioinformatics tutorial will apply biochemistry concepts using Chimera X and aims to:

1. Understand the effect of the amino acid side chain on the physicochemical properties, differences between amino acids, and the impacts generated.
2. Understand the main physicochemical properties used in these in silico analyses.
3. Guide students in visualizing and interpreting the following physicochemical properties using Chimera X: Electrostatic potential and hydrophobicity.
4. Explain how these properties can influence interactions between different molecules.

## **3. THEORETICAL BASIS**

The HIV immune response is complex and involves several steps throughout the infection, including interacting viral proteins with cellular receptors. Upon contact with the virus, host cell proteins are incorporated into the lipid layer of the viral envelope, which contains an anchored glycoprotein (gp120). Thus, an interaction occurs between HIV and target cells through some receptors and also gp120 (1).

The role of neutralizing antibodies in the immune response to viral infections, such as those caused by HIV, is particularly important. Their action includes binding to specific epitopes on the virus's surface, often on glycoproteins such as gp120, causing a blockage and preventing the infection of new cells (2). In this sense, a detailed understanding of these interactions and developing effective neutralizing antibodies are essential for preventing and treating HIV infection.

For antigen-antibody interactions to occur, different properties of these components must be considered. This binding often occurs through Van der Waals forces, electrostatic affinity, exposure of regions according to hydrophobicity, and formation of hydrogen bonds, disulfide bonds, and salt bridges. The side chain of amino acids plays a crucial role in the folding of the protein structure and the interaction between the antigen and the antibody by generating properties that determine the interaction in specific regions. Thus, a protein's set of amino acids provides a protein's hydrophobicity profile and electrostatic potential. Knowing how each amino acid interferes with these profiles, it is possible to propose changes in the sequence that lead to a better interaction with a target of interest (3).

## **4. METHODOLOGICAL PROCEDURES**

The following topic will cover three different concepts within the area of ​​Biochemistry, which will be applied to develop different approaches to physical-chemical properties using Chimera X. It is also important to emphasize the importance of not translating the pages so that the procedures' names and steps are not changed.

### **4.1. Concept 1: Electrostatic potential**

This first stage of procedures aims to establish the following knowledge: what electrostatic potential is, how it influences interactions between proteins, and how it varies between different proteins.

In the context of molecular interactions, electrostatic potential refers to the energy of a charge in an electric field. This parameter is crucial for analyzing biomolecules to understand how molecules interact with their environment and may influence bonds. Furthermore, electrostatic interactions play a role in the stability of molecules since these interactions directly depend on the charges on molecular surfaces and the polarity of solvents (4).

The electrostatic potential can be assessed through heat maps, which show the distribution of the potential around a molecule, which can be done through Chimera X. These maps allow the study of interactions between molecules, such as the binding of targets to proteins since they allow the observation of areas of positive, negative and neutral charge on the molecular surface.

The analysis can be interpreted using a color scale. Red indicates regions of negative potential with a higher density of electrons, which are more attractive to positive charges. Blue indicates positive potential, with a lower density of electrons and, consequently, a greater attraction to negative charges. In addition, there is a white color that denotes areas of neutral potential (Figure 1).

- Step 1:

| 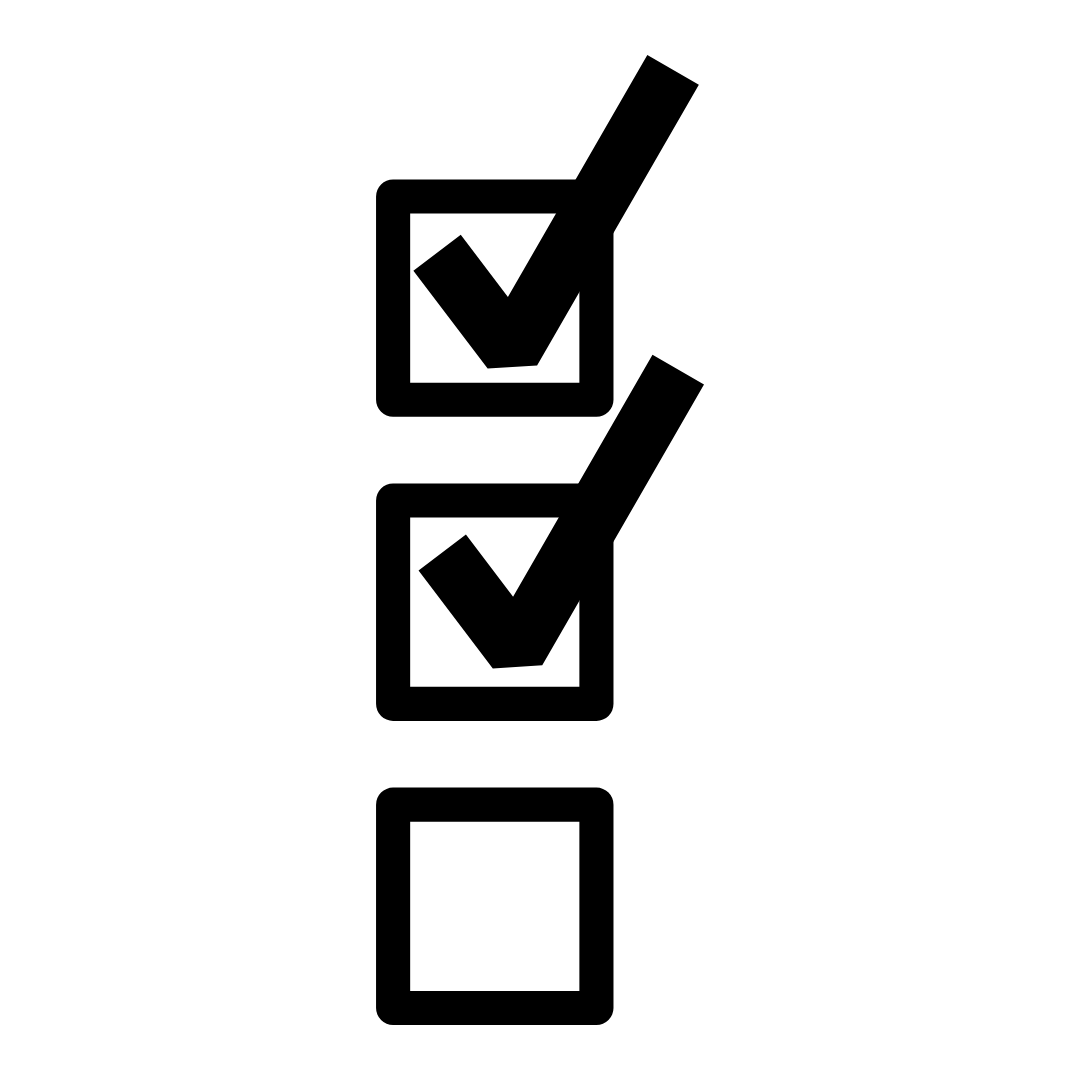 | 1. Select two or more similar structures. At this point, we suggest using the PDB: 2NY7 and PDB: 7RDW files. These analyses apply when comparing multiple similar proteins, such as variants or proteins from the same family.  \| 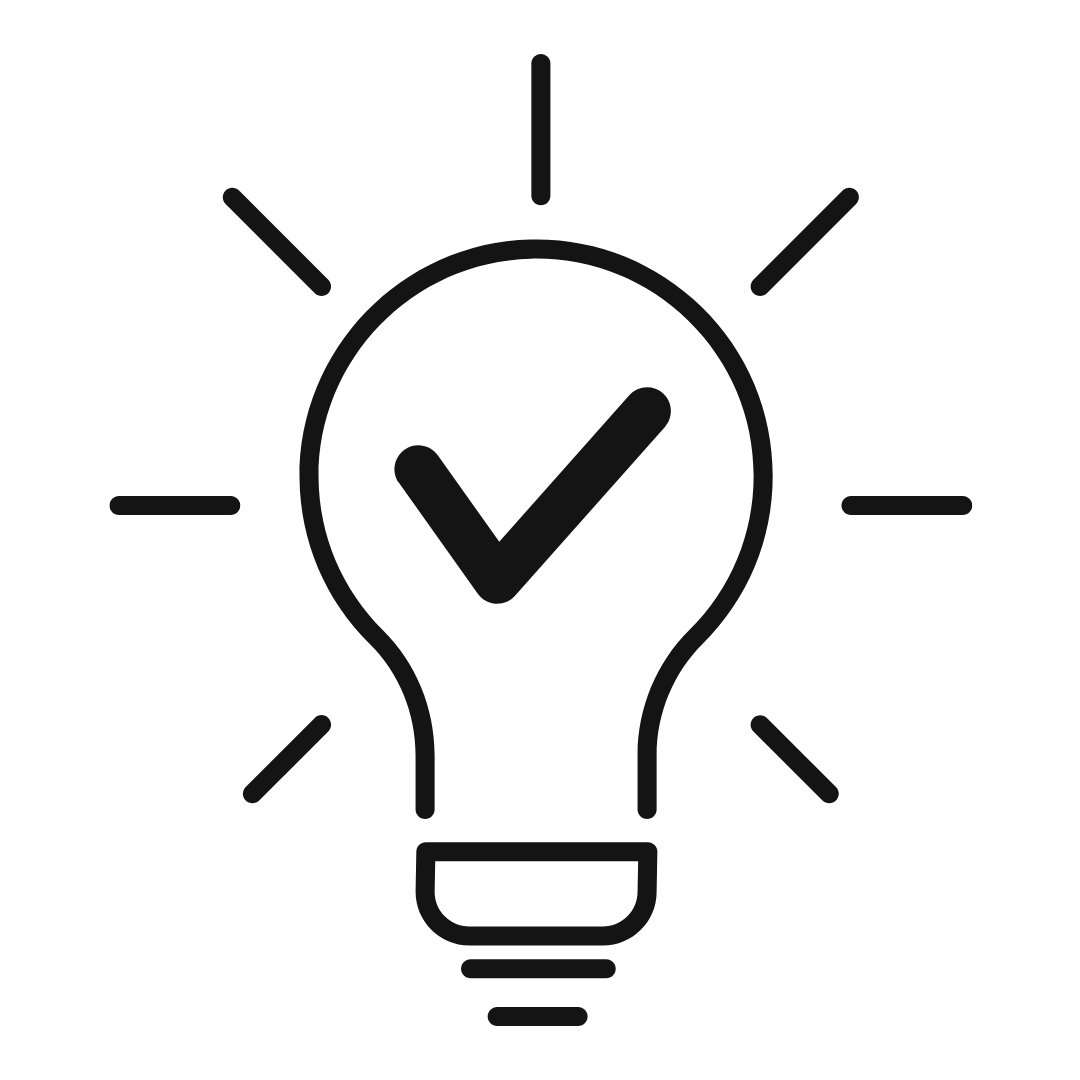 \| To identify similar proteins, it is suggested to use the BLASP tool (https://blast.ncbi.nlm.nih.gov/Blast.cgi) \| \| --- \| --- \|  1. The download must be in .pdb format from the Protein Data Bank (https://www.rcsb.org/), according to the following path:  \| *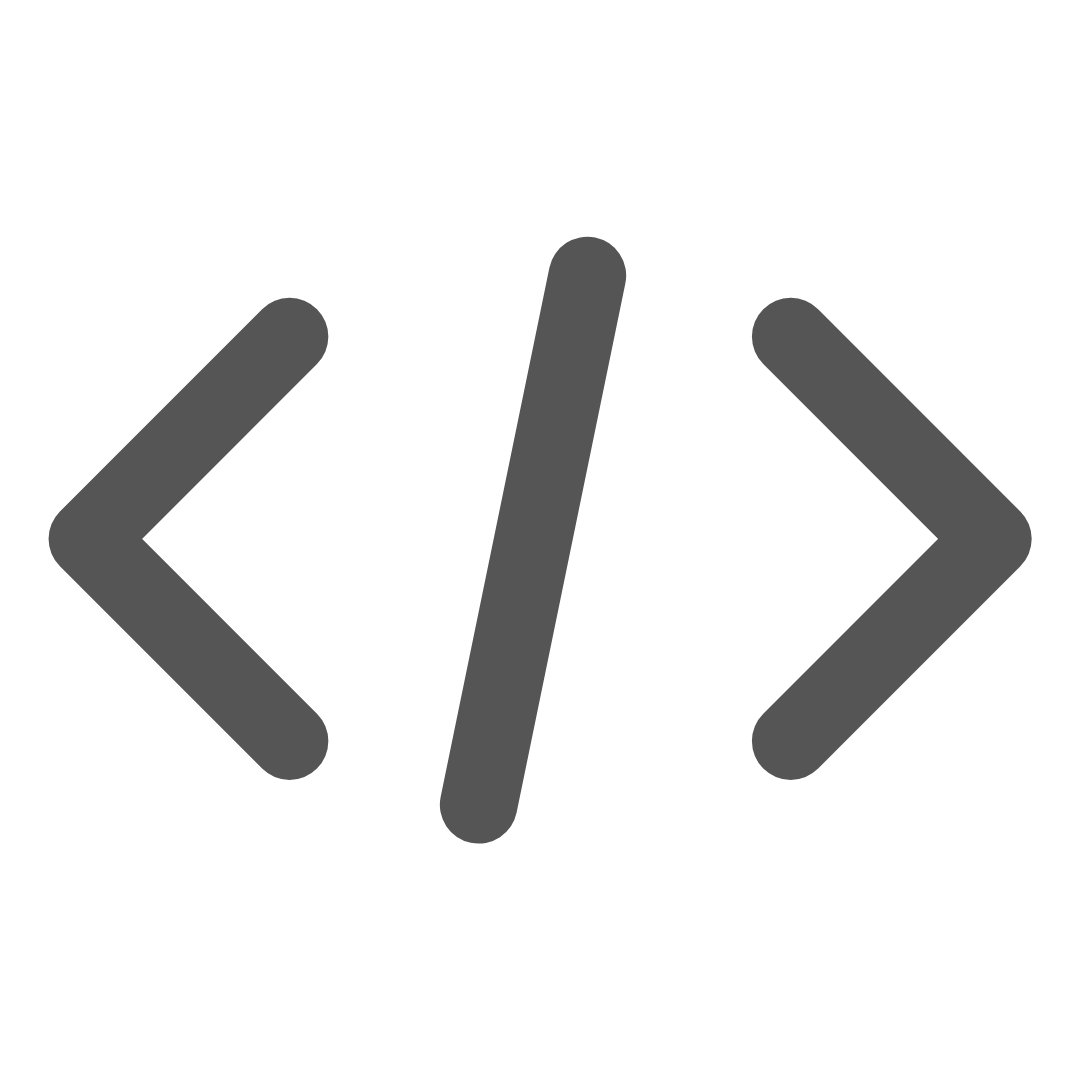* \| *3D structure search “code” > Download Files > PDB Format** \| \| --- \| --- \|   *See tutorial 1 if in doubt.   1. Open the Chimera interface using the shortcut generated on the desktop. Click on the “Open” menu available in the “Home” tab, select the folder where the files were saved, and select simultaneously (keeping the Ctrl key pressed) the proteins you want to compare, in this case, 2NY7 and 7RDW.  \| 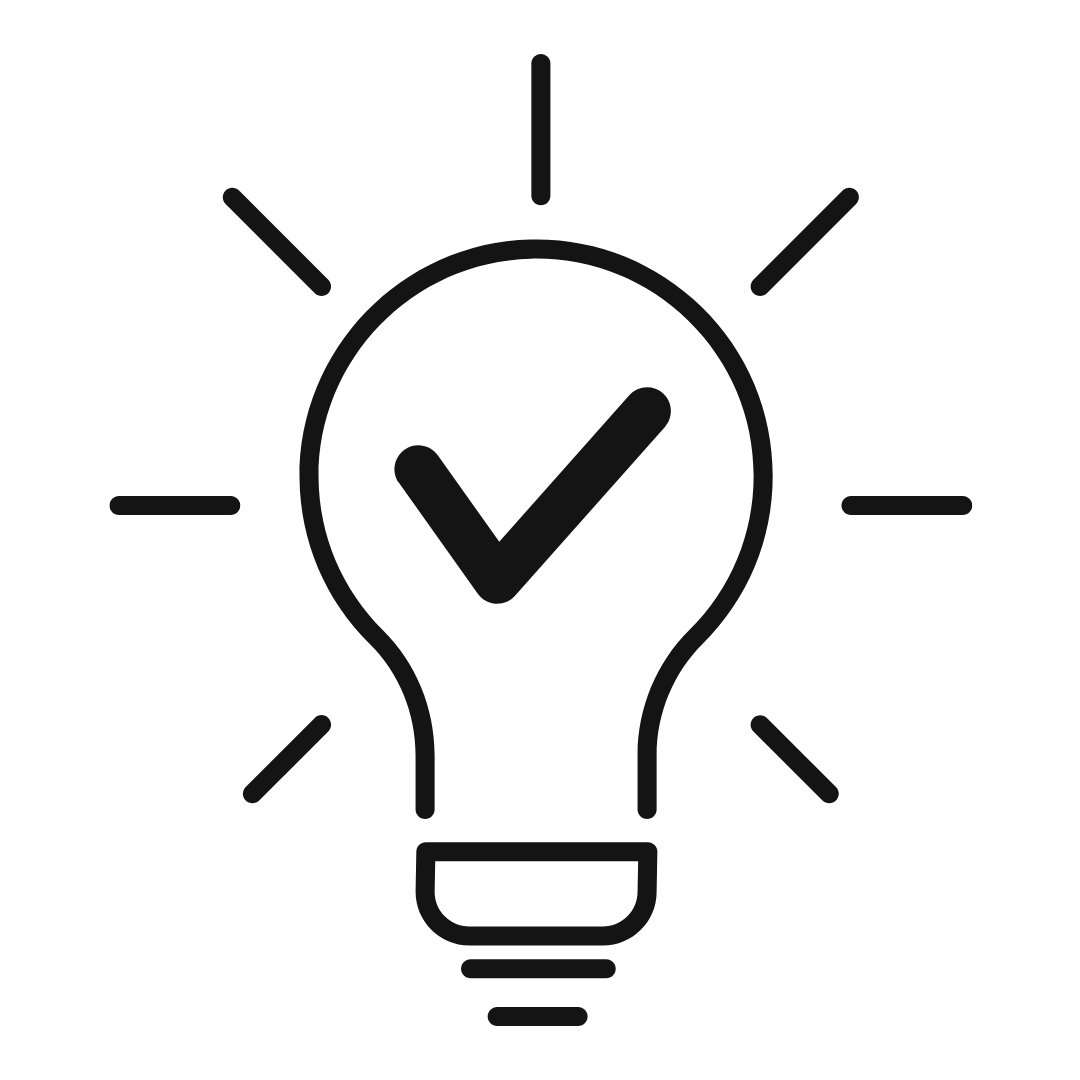 \| The proteins can be visualized from this process in the Chimera X interface. \| \| --- \| --- \|  1. When opening the structures in Chimera X, select the “Molecule Display” tab and select the “Electrostatic” feature, and then in the command line, type: “coulombic range -3.3”  \| 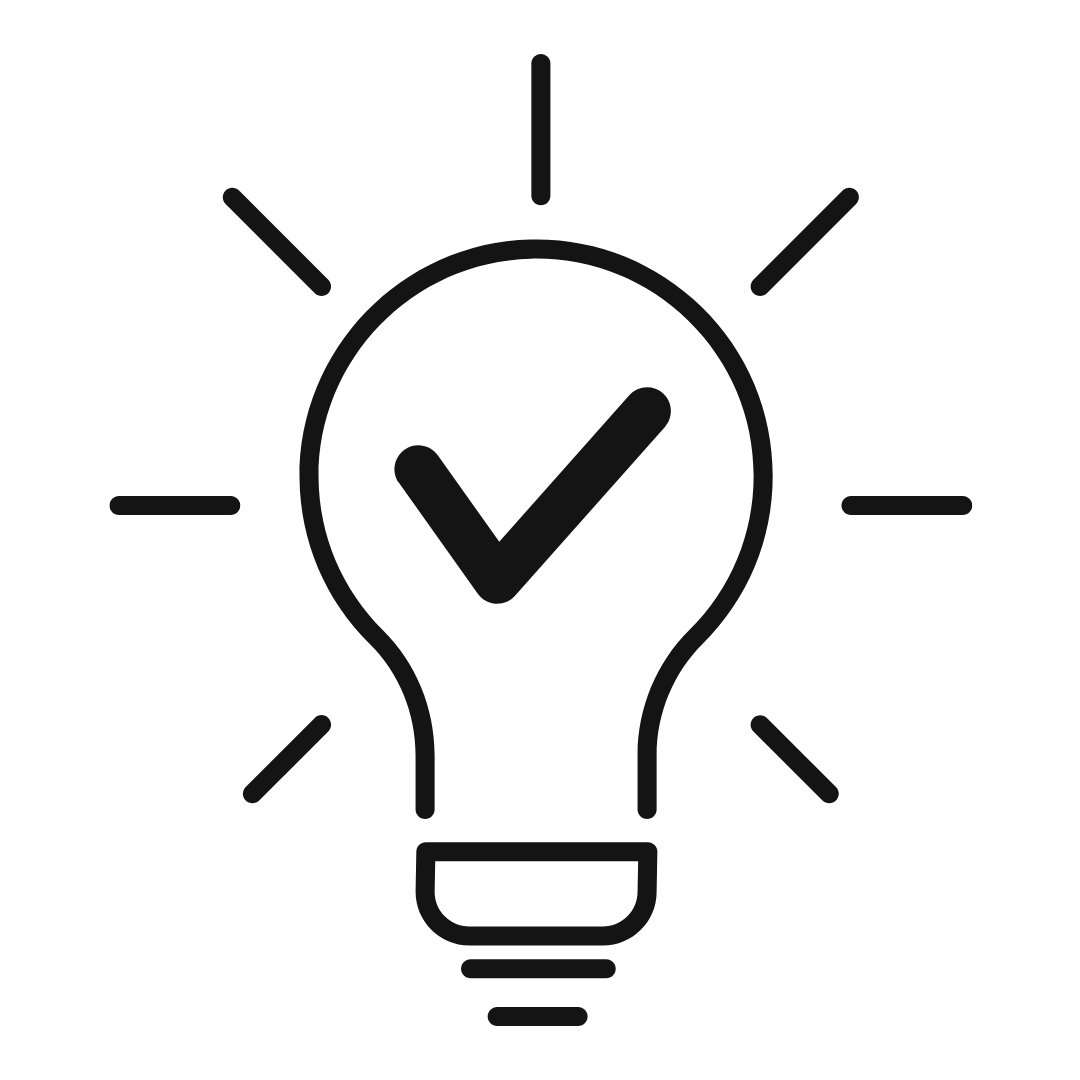 \| The range parameter specifies the electrostatic potential values ​​between -3 and +3 kcal/(mol e) that will be colored on the molecule's surface. \| \| --- \| --- \|  1. After generating the electrostatic potential maps, use the Matchmaker command to align and compare the two structures. Matchmaker is a tool that aligns two proteins based on the similarity of their sequences and three-dimensional structures, facilitating direct comparison. To run Matchmaker, follow these steps:  \| *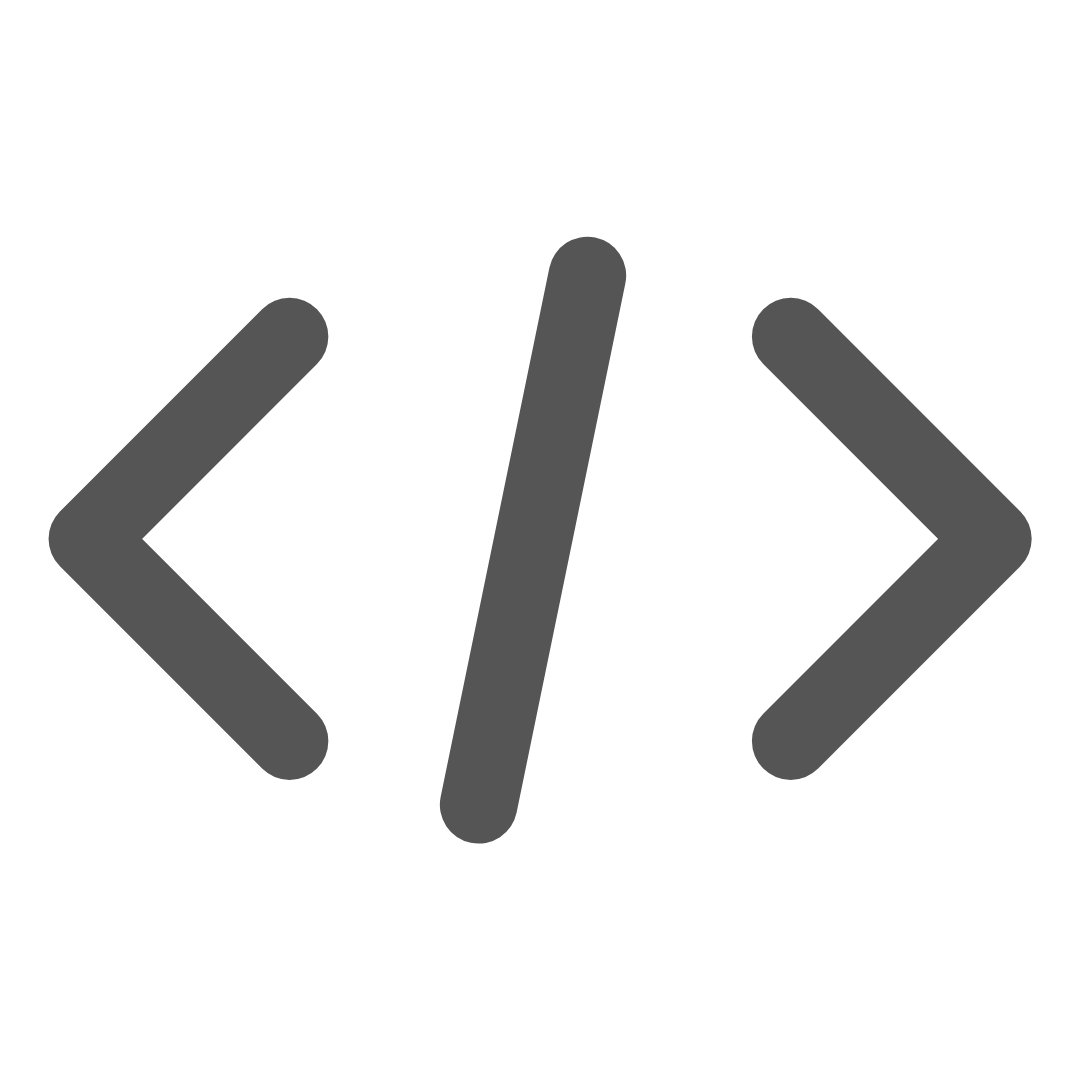* \| Click on “Tools” on the taskbar > “Structure Analysis” > “Matchmaker” \| \| --- \| --- \|   *See tutorial 2 if in doubt.   1. After alignment, use the Models tab to switch between structures and perform a detailed analysis of each protein individually. In the tab, deselect the visibility of one protein to focus on the analysis of the other, and thus, perform a detailed interpretation of the generated electrostatic potential map. |
| --- | --- | --- | --- | --- | --- | --- | --- | --- | --- | --- | --- |

At the end of Step 1, it will be possible to compare areas of interest, such as regions of high negative or positive charge, to interpret potential functional differences between the molecules of interest, in this case, 7RDW and 2NY7 (Figure 1). This comparative analysis is useful in protein-ligand interaction studies or understanding structural changes related to biological function.


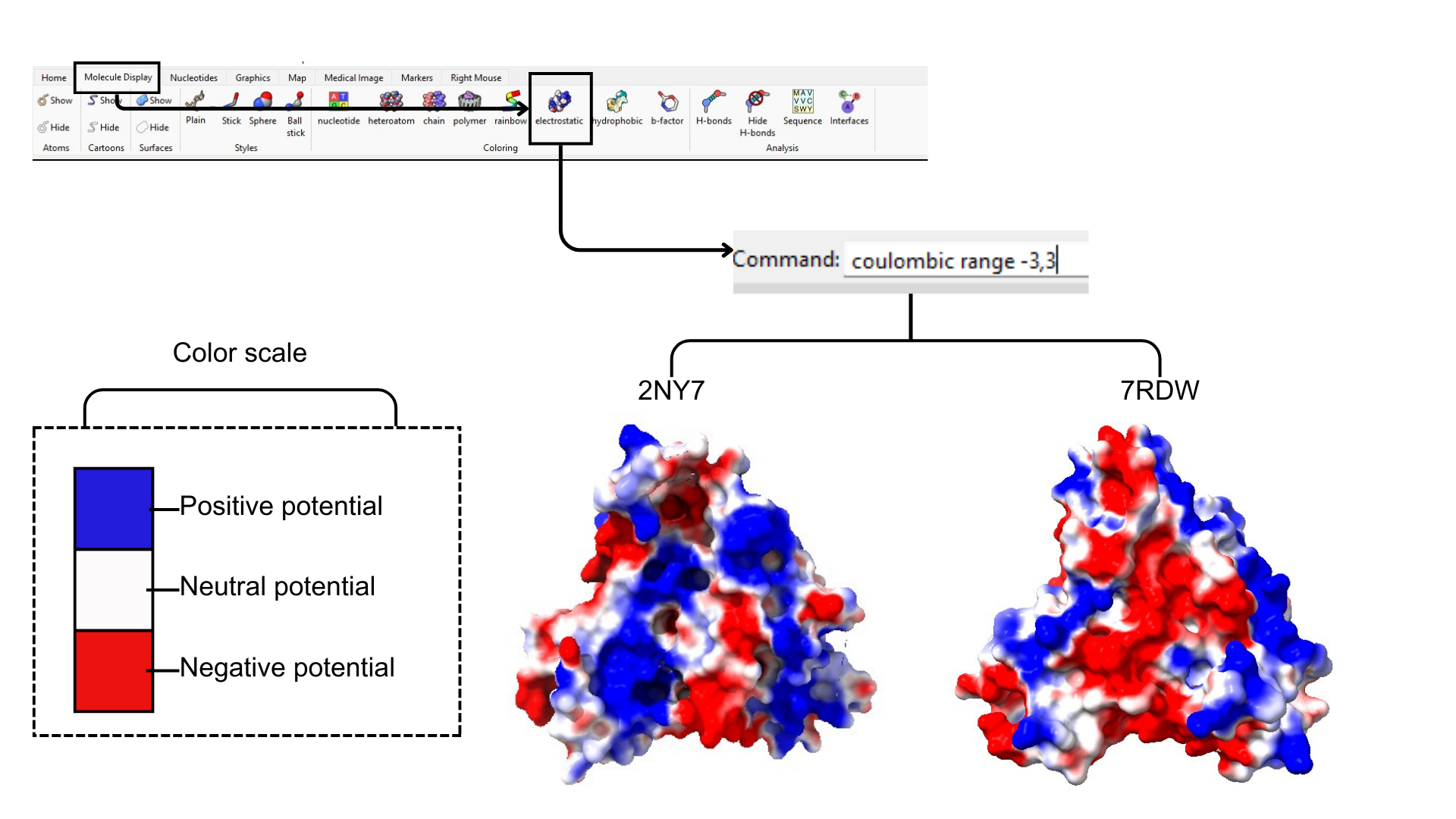


Figure 1. Comparison of the electrostatic potential between two similar proteins. A color scale is used to interpret the electrostatic profile, where blue indicates a positive potential, while red and white suggest a negative and neutral potential, respectively.

Source: Compiled by the authors

### **4.2. Concept 2: Hydrophobicity**

This second stage of procedures aims to establish the following knowledge: hydrophobicity, how it influences interactions between proteins, and how it varies between different proteins.

Hydrophobicity is a physicochemical property in molecular interactions related to the tendency to repel or attract water. In this sense, hydrophobic portions tend to repel water (polar molecules), while hydrophilic ones attract it. In this sense, the hydrophobic effect facilitates the aggregation of nonpolar substances, increasing the interaction between ligands with this characteristic. Hydrophilic regions, on the other hand, tend to bind more easily with more polar regions (5).

This parameter is crucial for analyzing biomolecules to understand how molecules interact with the environment and may influence bonds. Furthermore, hydrophobicity plays an important role in the stability of molecules since the folding of a protein is directly related to its hydrophobicity profile. More hydrophobic regions tend to be located more in the protein core (central region). In contrast, hydrophilic residues are on the protein's surface, interacting with the medium, usually aqueous, and with the ligands (6).

Hydrophobicity on a protein surface can be represented using a color scale, which shows the distribution of hydrophobicity around a molecule. This can be done using Chimera X. These scales allow the study of interactions between molecules, such as the binding of targets to proteins, since they allow observing hydrophobic, hydrophilic, and neutral areas on the molecular surface.

In interpreting the color scale, blue indicates more hydrophilic regions, which tend to interact with polar regions. Yellow indicates more hydrophobic regions, which tend to interact with nonpolar regions. In addition, white denotes areas of neutral potential (Figure 1).

- Step 2:

| 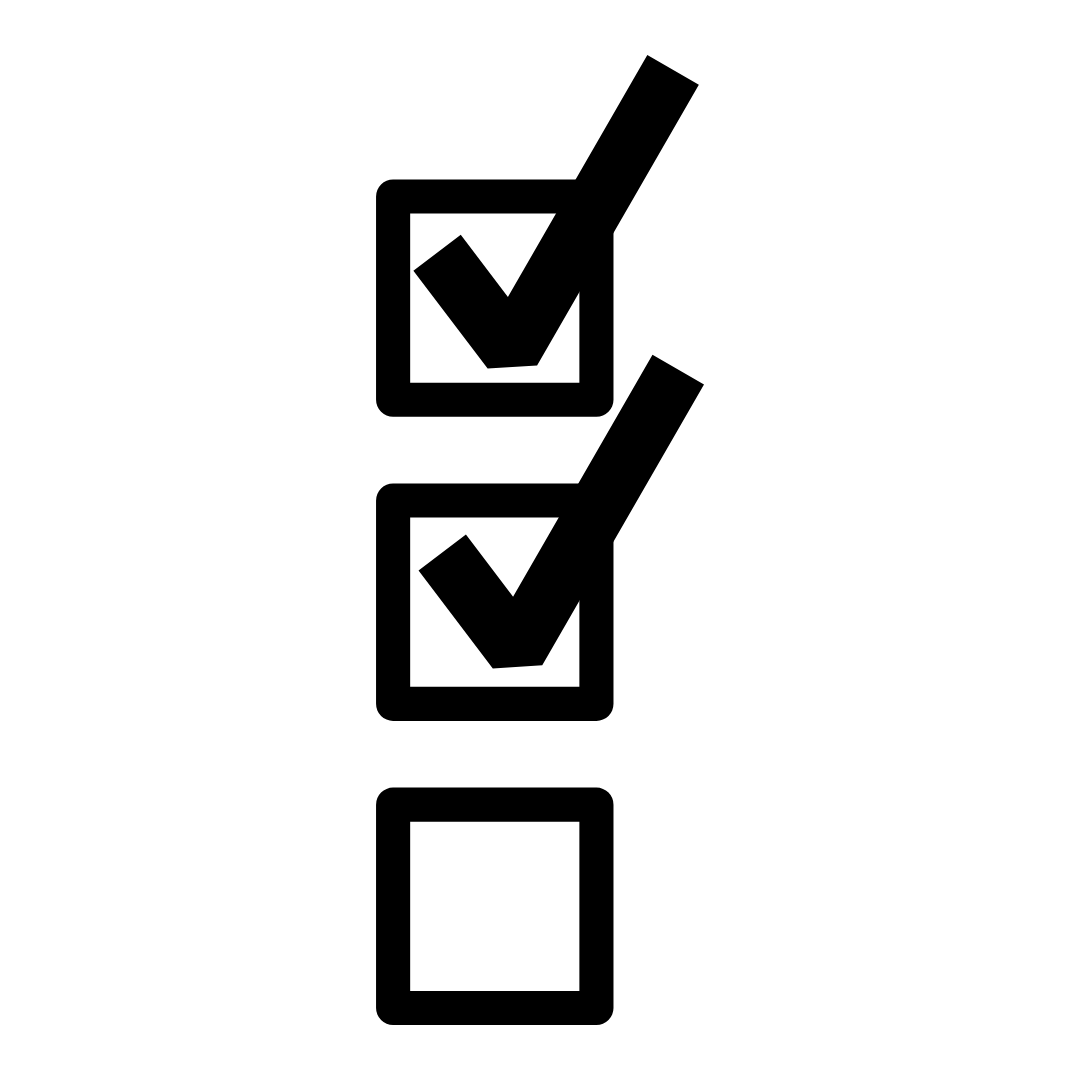 | 1. To analyze other properties, such as hydrophobicity, you must proceed to item 4 of step 1 of this tutorial. However, you must click on hydrophobicity instead of selecting the electrostatic potential.  \| 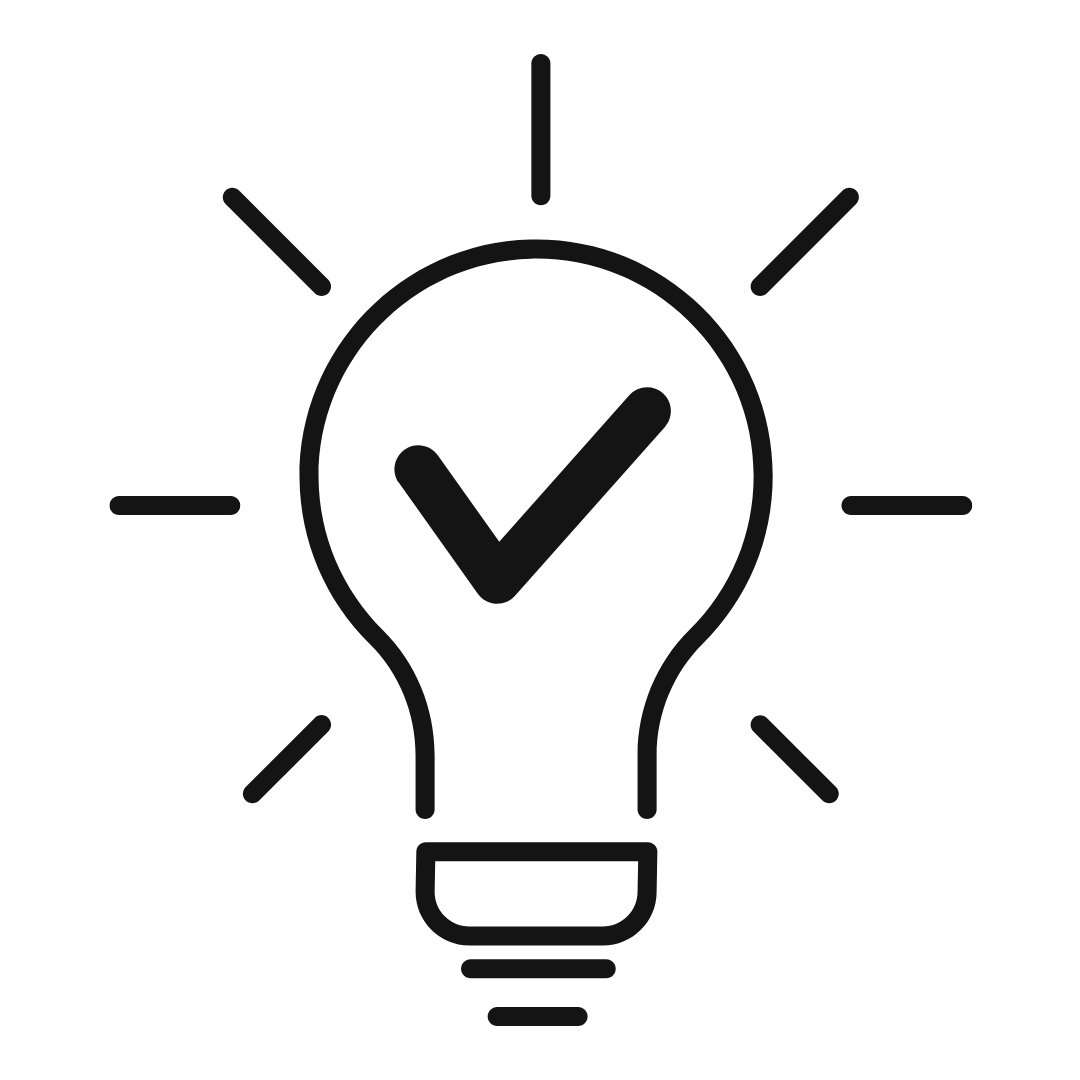 \| For this parameter, it is not necessary to configure the range via the command line. \| \| --- \| --- \|  1. Items 5 and 6 of this tutorial must be followed to compare proteins of interest.  \| 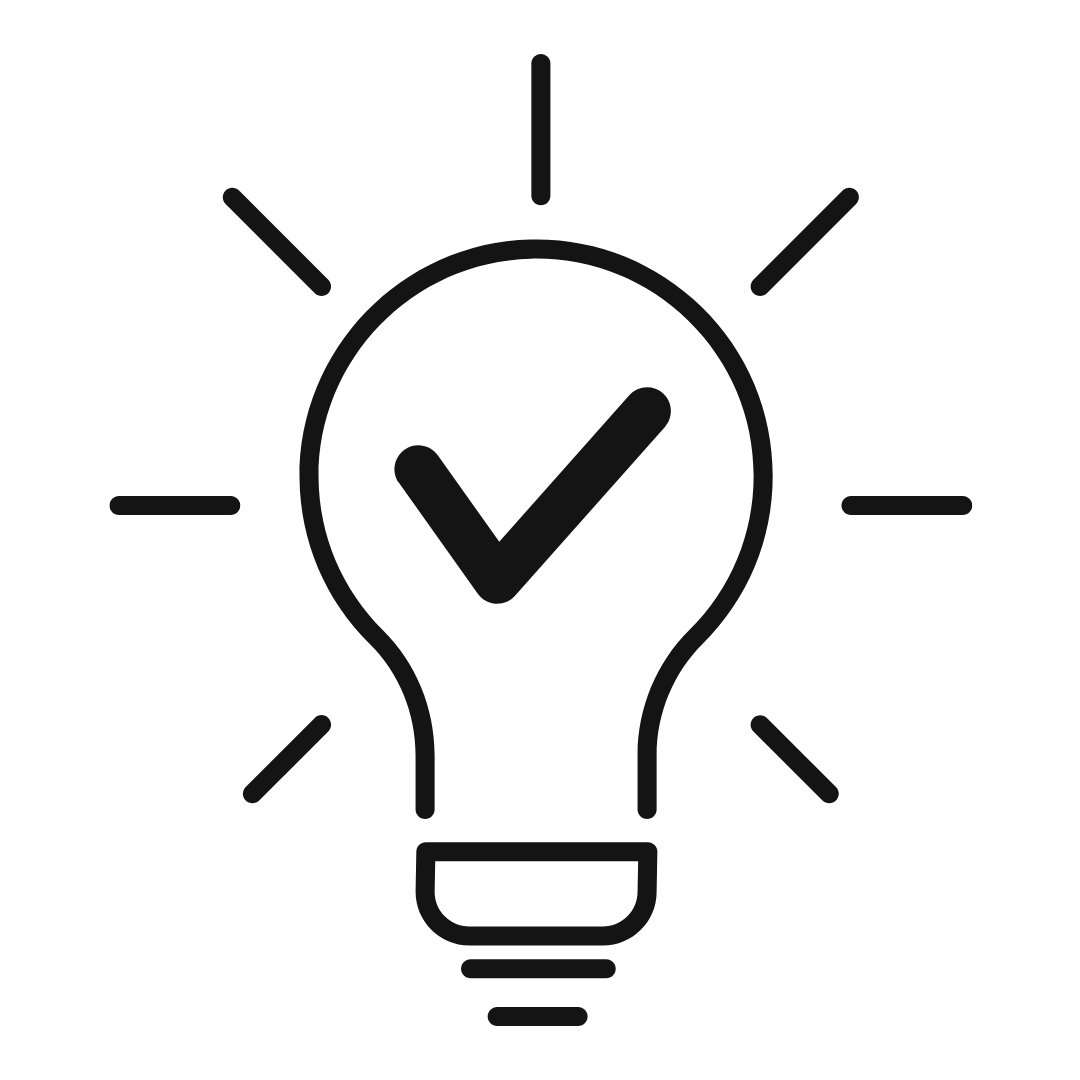 \| On the color scale, hydrophilic regions are represented by blue, and neutral and hydrophobic regions by white and red, respectively. \| \| --- \| --- \| |
| --- | --- | --- | --- | --- | --- |

The procedures covered in Step 2 allow a clear visualization of the hydrophilic, hydrophobic, and neutral regions of one or more proteins (Figure 2). This analysis is essential to understanding the properties of the molecules of interest, such as structure, stability, and prediction of active sites.


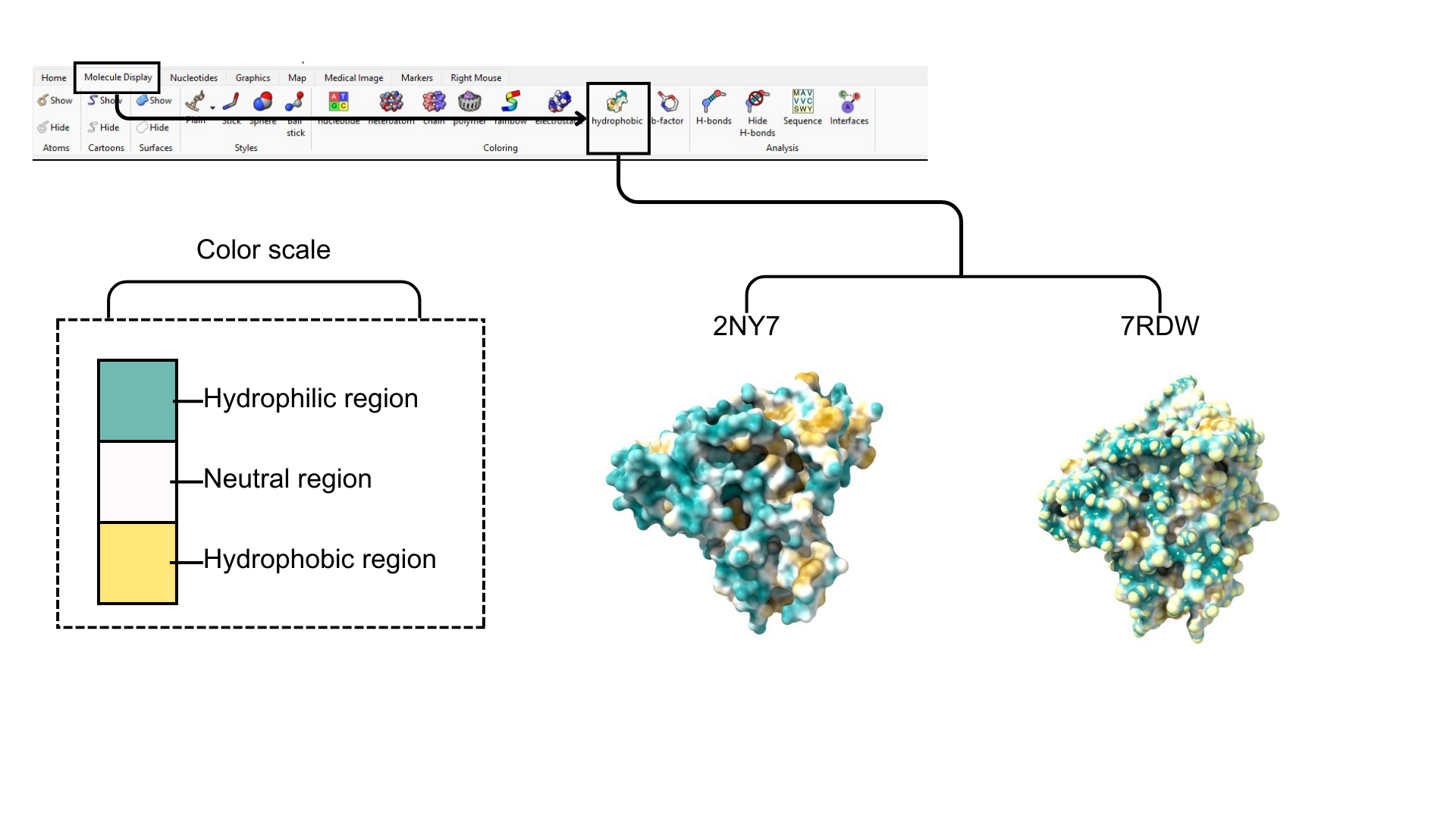


Figure 2. Comparison of hydrophobicity between two similar proteins. A color scale is used to interpret the electrostatic profile where blue indicates a hydrophilic region, while red and white suggest hydrophobic and neutral regions, respectively.

Source: Compiled by the authors

## **5. QUESTIONNAIRE**

A questionnaire was developed to evaluate the learning process with this tutorial. Therefore, it is suggested that it be applied before and after the Tutorial, with the objective of the student and teacher being to perceive the knowledge acquired through it and adapt it for the class.

1. How important is comparing two structurally similar proteins but with some altered amino acids?
2. How does the electrostatic potential interfere with interactions with ligands?
3. How does hydrophobicity interfere with interactions with ligands?
4. What does the range applied to the electrostatic potential mean?
5. Why use the matchmaker resource in the analysis of physicochemical properties?
6. How does hydrophobicity interfere with protein folding?

## **6. CONCLUSION**

Upon completing this structural bioinformatics tutorial on biochemistry concepts through Chimera X, the student is expected to have acquired essential practical and theoretical skills to compare more than one protein and understand the implications of physicochemical properties in interactions with ligands. This knowledge includes the ability to visualize and manipulate three-dimensional structures of proteins and identify the electrostatic potential and hydrophobicity of different regions of the same protein.

Furthermore, it is worth noting that the knowledge acquired in the tutorial provides a starting point for studies on mastering bioinformatics techniques. By understanding these techniques, abstract concepts can be made more visible in biochemistry and advances in research in any area involving proteins.

## **REFERENCES**

1. Schwartz SA, Nair MPN. Current concepts in human immunodeficiency virus infection and AIDS. Clin Diagn Lab Immunol [Internet]. 1999 [citado 13 de junho de 2024];6(3):295–305. Disponível em: https://pubmed.ncbi.nlm.nih.gov/10225826/

2. Timofeeva A, Sedykh S, Nevinsky G. Post-immune antibodies in HIV-1 infection in the context of vaccine development: A variety of biological functions and catalytic activities. Vaccines (Basel) [Internet]. 2022 [citado 13 de junho de 2024];10(3):384. Disponível em: http://dx.doi.org/10.3390/vaccines10030384

3. Farafonov VS, Lebed AV, Nerukh DA, Mchedlov-Petrossyan NO. Estimation of nanoparticle’s surface electrostatic potential in solution using acid–base molecular probes I: in silico implementation for surfactant micelles. J Phys Chem B [Internet]. 2023;127(4):1022–30. Disponível em: http://dx.doi.org/10.1021/acs.jpcb.2c07012

4. Reverberi R, Reverberi L. Factors affecting the antigen-antibody reaction. Blood Transfus [Internet]. 2007;5(4):227–40. Disponível em: http://dx.doi.org/10.2450/2007.0047-07

5. Biela A, Nasief NN, Betz M, Heine A, Hangauer D, Klebe G. Dissecting the hydrophobic effect on the molecular level: the role of water, enthalpy, and entropy in ligand binding to thermolysin. Angew Chem Int Ed Engl [Internet]. 2013;52(6):1822–8. Disponível em: http://dx.doi.org/10.1002/anie.201208561.

6. Tang S. Predicting protein surface property with its surface hydrophobicity. Protein Pept Lett [Internet]. 2021;28(8). Disponível em: http://dx.doi.org/10.2174/09298665mte0tndqb4
