# Supplementary material for: Chimera X Interface to Enhance Understanding in Biochemistry and Immunology
Source: Biochem Mol Biol Educ. 2025 Nov 14;54(1):92–102. doi: 10.1002/bmb.70025 (PMC12877970; doi:10.1002/bmb.70025)
Supplement: Supplementary file 4 — Supporting Information 4. Table S1. Structure of the student questionnaire with respective blocks and items. Supporting Information 5. Table S5. Raw data and Content Validity Index (CVI) for each questionnaire item. [file BMB-54-92-s004.docx]

**Supplemental Material 4. Structure and items of the student questionnaire**

Table S1. Structure of the student questionnaire with respective blocks and items

| **Blocks** | **Questions and items** |
| --- | --- |
| Part I: Identification | 1. What is your full name? |
|  | 2. What course are you enrolled in and what is your current semester? |
|  | 3. Have you had any previous experience with the Chimera X tool? |
| Part II: Assessing Tutorial Understanding | 4. What is the structure of a monoclonal antibody? |
|  | 5. Explain the concept of antigen-antibody interaction and give examples of binding types. |
|  | 6. What is the role of the gp120 glycoprotein in the HIV infection process? |
|  | 7. The structures 1HZH and 2NY7 represent which biomolecules in the context of the tutorial, and what are their leading chains? |
|  | 8. Explain the function of Matchmaker in Chimera X and its usefulness. |
|  | 9. What is the importance of the Protein Data Bank? |
|  | 10. What is the name of the region of the antibody that interacts with the antigen? And vice versa. |
|  | 11. Suggest a reason for studying antigen-antibody interactions. |
|  | 12. Name a feature of the Chimera interface that enables the study of interactions. |
|  | 13. Why do you think visualizing structures and their interactions is important in microbiology? What applications do you see for this? |
| Part III: Assessment of the Applicability of the Tutorial and the Tool | 14. The tutorial contributed to my understanding of the concepts covered in the class. |
|  | 15. The language used in the tutorial was clear and accessible. |
|  | 16. The tutorial content was relevant to the course. |
|  | 17. The tutorial facilitated the practical application of theoretical concepts. |
|  | 18. I felt more confident answering questions or completing activities after the tutorial. |
|  | 19. The tutorial format (e.g., visual, interactive, text) was appropriate for my learning. |
|  | 20. I would recommend using this type of tutorial in other classes. |
|  | 21. What was the most positive aspect of the tutorial? |
|  | 22. Did you encounter any difficulties while using the tutorial? If so, what? |
|  | 23. Suggestions for improvement for future tutorials: |

**Supplemental Material 5. Raw data and Content Validity Index (CVI) per questionnaire item**

Table S5. Raw data and Content Validity Index (CVI) for each questionnaire item

| **Student** | **Question 1** | **Question 2** | **Question 3** | **Question 4** | **Question 5** | **Question 6** | **Question 7** |
| --- | --- | --- | --- | --- | --- | --- | --- |
| 1 | 4 | 4 | 5 | 5 | 4 | 5 | 4 |
| 2 | 5 | 5 | 5 | 5 | 5 | 5 | 5 |
| 3 | 5 | 5 | 4 | 5 | 4 | 5 | 5 |
| 4 | 5 | 5 | 5 | 5 | 4 | 5 | 5 |
| 5 | 5 | 5 | 5 | 5 | 5 | 5 | 5 |
| 6 | 5 | 5 | 4 | 5 | 5 | 5 | 5 |
| 7 | 5 | 5 | 5 | 5 | 5 | 5 | 5 |
| 8 | 4 | 4 | 5 | 5 | 4 | 5 | 5 |
| 9 | 5 | 4 | 5 | 5 | 5 | 5 | 5 |
| 10 | 5 | 5 | 5 | 5 | 5 | 5 | 5 |
| 11 | 5 | 5 | 5 | 5 | 5 | 5 | 5 |
| 12 | 5 | 5 | 5 | 5 | 4 | 5 | 5 |
| 13 | 3 | 4 | 3 | 3 | 2 | 4 | 3 |
| 14 | 5 | 5 | 5 | 5 | 5 | 5 | 5 |
| 15 | 5 | 5 | 5 | 5 | 5 | 5 | 5 |
| 16 | 3 | 4 | 3 | 5 | 5 | 5 | 5 |
| 17 | 4 | 4 | 4 | 4 | 4 | 4 | 4 |
| 18 | 5 | 5 | 5 | 5 | 5 | 5 | 5 |
| CVI | 0.889 | 1.000 | 0.889 | 0.944 | 0.944 | 1.000 | 0.944 |
